# Isolation and reactivity of an alkyl-substituted germanium(I) radical anion

**DOI:** 10.1038/s41467-026-74699-1

**Published:** 2026-06-19

**Authors:** Chenting Yan, Yanxue Bi, Nanfeng Zhou, Yingying Xi, Chanyu Li, Gaofeng Bian, Ru-De Lin, Yihan Zhou, Hanjiao Chen, Zhifang Li, Zhaowen Dong

**Affiliations:** 1 Key Laboratory of Organosilicon Chemistry and Material Technology, Ministry of Education, Zhejiang Key Laboratory of Organosilicon Material Technology, College of Material, Chemistry and Chemical Engineering, Hangzhou Normal University, Hangzhou, China; 2 Key Laboratory of Green Chemistry and Technology, Ministry of Education, College of Chemistry, Sichuan University, Chengdu, PR China; 3 Analytical & Testing Center, Sichuan University, Chengdu, PR China

**Keywords:** Ligands, Synthetic chemistry methodology, Chemical bonding

## Abstract

The isolation of low-valent main group radical species remains a fundamental challenge in synthetic chemistry due to their intrinsic instability. Herein, we report the successful isolation and full characterization of an alkyl-substituted germanium(I) radical anion predominantly stabilized by steric protection with minimal electronic perturbation. This radical anion exhibits thermal stability in both the solid state and in solution at ambient temperature, enabling thorough structural, spectroscopic and reactivity investigations. EPR spectroscopy and DFT calculations consistently verify predominant localization of the unpaired electron at the germanium center. Furthermore, the Ge(I) radical anion presents unique radical reactivity patterns demonstrating by its reactivity investigation. This work represents a rare example of an isolable low-valent group 14 radical species.

## Abstract (web summary)

Low-valent main group radicals are difficult to isolate because they are highly unstable. Here, the authors report a stable germanium radical anion and reveal its structure, bonding and distinctive reactivity.

## Introduction

Radicals are fundamental intermediates in synthetic chemistry, catalysis, polymer science, and energy related processes^1–18^. Over the past century, advances in the design of stable organic radicals have significantly advanced radical chemistry and functional materials, enabling diverse applications such as controlled radical polymerization and molecular spin-based systems^19–26^. While carbon-centered radicals have been extensively studied^27–30^, extending radical chemistry to heavier main group elements remains a considerable challenge, largely due to the tendency of these open-shell species to dimerize or decompose rapidly^31–39^. However, recent progress in steric shielding and electronic delocalization of the spin density has facilitated the isolation of a growing family of stable p-block radicals^40–49^. These species exhibit unique electronic configurations and reactivity profiles that differ markedly from those of traditional organic radicals.

**Fig. 1:**
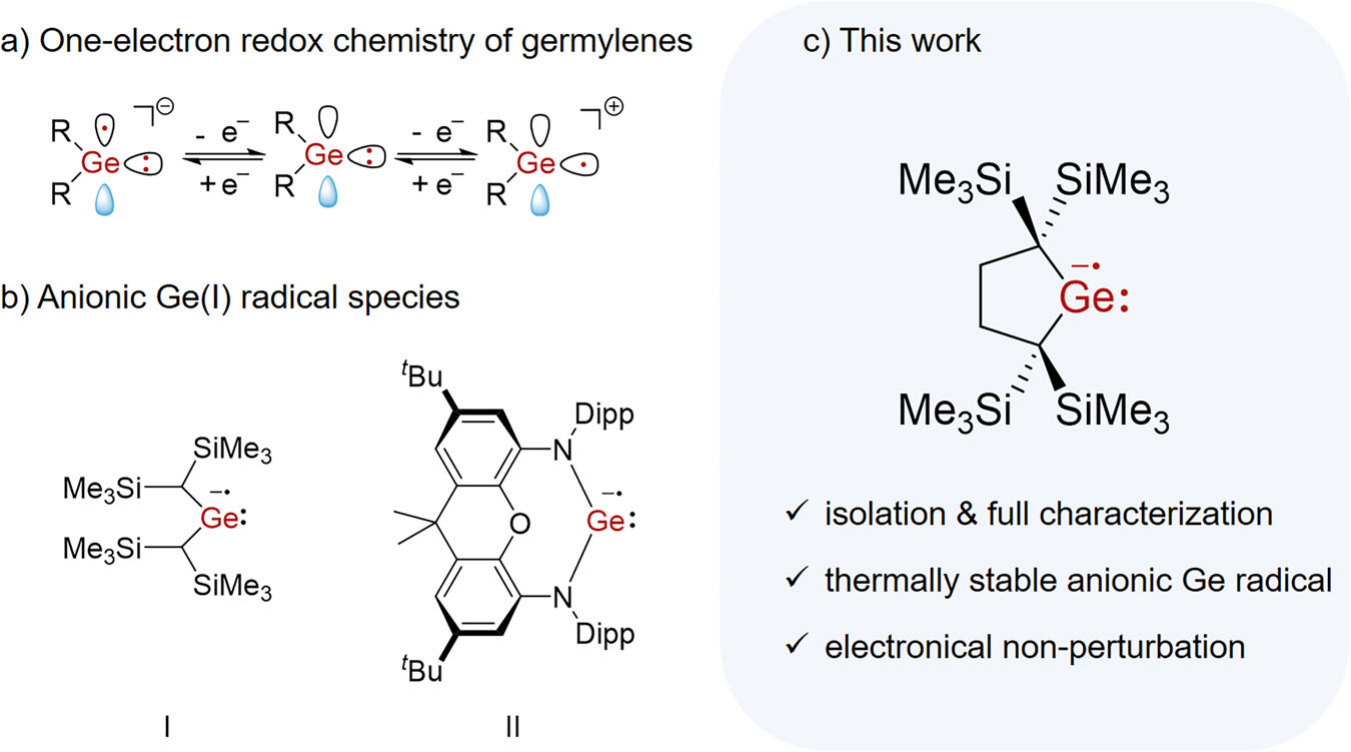
Conceptual overview and Background. **a** Oxidized and reduced forms of germylenes. **b** Selected examples of anionic Ge(I) radical species. **c** The alkyl-substituted germanium(I) radical anion reported in this work.

In this context, germanium-centered radicals have emerged as prominent examples of open-shell species among heavier main group elements^50–52^. Over the past two decades, well-defined neutral Ge(I)^53–59^ and Ge(III)^60–65^ radicals have been stabilized through carefully tailored steric or electronic environments. More recently, research has expanded to ionic germanium radicals, including Ge(III) radical cations^66^ and Ge(I) radical anions^67,68^. Their isolation and structural characterization provides new opportunities for understanding the chemical properties of low-valent main group radical systems. Among current synthetic strategies, one-electron redox chemistry of germylenes has proven effective strategy for generating such ionic germanium radicals (Fig. 1a)^40,69^.

Despite these advances, isolable Ge(I) radical anions remain exceedingly rare. The alkyl-substituted example, reported in 1995^67^, was characterized only by EPR spectroscopy and could not be isolated in substance (Fig. 1b, I). Nearly three decades later, the reduction of xanthene-based diamido-supported germylenes yielded the crystallographically characterized Ge(I) radical anions^68^. However, this species depended on diamido ligands and were persistent only below approximately –40 °C (Fig. 1b, II). To date, an alkyl-substituted, electronically non‑perturbing Ge(I) radical anion has not yet been isolated, leaving the intrinsic properties of such heteroatom-free radical systems unexplored.

Herein, we report the synthesis, isolation and full characterization of an alkyl-substituted germanium(I) radical anion (Fig. 1c). Stabilized exclusively by σ-donating alkyl ligands, this species exhibits exceptional thermal stability in both the solid state and solution at ambient temperature. Detailed structural and spectroscopic analyses provide direct insight into the intrinsic electronic and structural properties of a minimally perturbed Ge(I) radical anion.

## Results and discussion

### Synthesis and characterization of the germanium radical anions

**Fig. 2:**
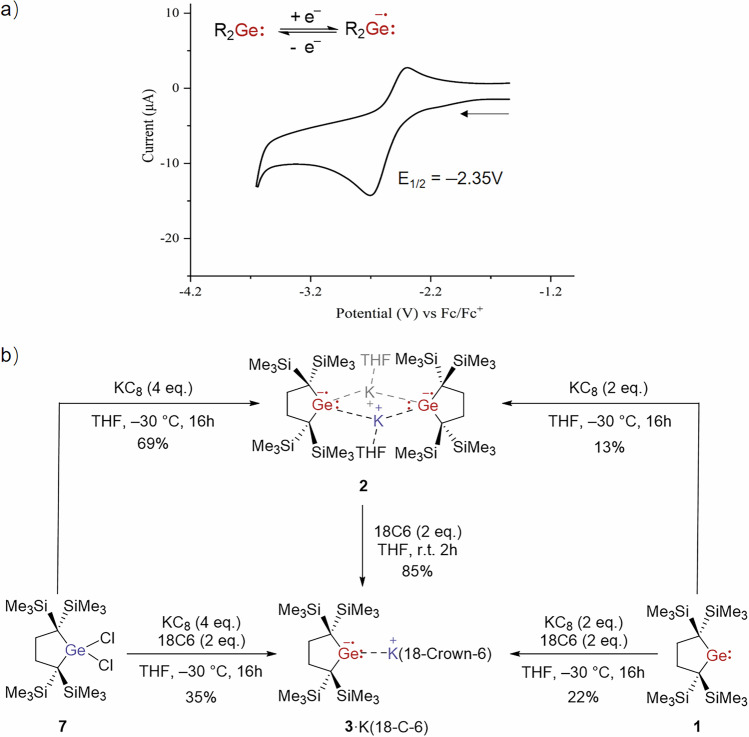
Cyclic voltammogram of **1** and synthesis of **2** and **3**·K(18-C-6). **a** Cyclic voltammogram of **1** at 0.1 V s^−1^ scan rate in THF / 0.1 M [*n-*Bu_4_N][PF_6_]. **b** Synthesis of germanium radical anions **2** and **3**·K(18-C-6).

Building upon the precedent of generating silicon radical anions via reduction of dialkylsilylene and tetrakis(di-*tert*-butylmethylsilyl)disilene^46,48,49^, we hypothesized that a similar redox transformation might be achievable for their germanium analogs^70^. Cyclic voltammetry (CV) of dialkylgermylene **1** in tetrahydrofuran (THF) showed a reversible one-electron reduction at E_1/2_ = –2.35 V (vs. Fc/Fc^+^), suggesting the accessibility of a corresponding germanium radical anion (Fig. 2a). Encouraged by these electrochemical results, we proceeded to target the synthesis of such a radical anion starting from germylene **1**.

Potassium graphite (KC_8_) was selected as the reductant for the reduction of germylene **1**. Treatment of **1** with two equivalents of KC_8_ in THF at –30°C resulted in an immediate color change from yellow to dark red. After stirring for 16 h and subsequent workup, the germanium(I) radical anion **2** was isolated as a dark red solid in 13% yield (Fig. 2b). Single crystals suitable for X-ray diffraction analysis were obtained from a concentrated hexane solution, which revealed a dimeric structure in the solid state.

Isolation of the monomeric species was achieved by subsequent treatment of the crude product of **2** with two equivalents of 18-crown-6 ether (18-C-6) in THF at room temperature, which afforded the germanium radical anion **3**·K(18-C-6) as orange crystals in 85% yield. Given the low yield of the previous synthesis, an alternative route to preparation of **2** was devised, involving the reduction of a cyclic dichlorogermane precursor **7** to give **2** in a moderate isolated yield of 69% (Fig. 2b). Compound **3**·K(18-C-6) is highly sensitive to air and moisture but exhibits remarkable thermal stability under inert conditions in both the solid state and in solution, showing no detectable decomposition at least one month at room temperature. The UV–vis spectra of **2** and **3**·K(18-C-6) in THF showed broad absorption bands at 464 nm (ε = 6.25 × 10^3 ^L·mol^-1^·cm^-1^) and 477 nm (ε = 6.73 × 10^2 ^L·mol^-1^·cm^-1^), respectively, both displaying a notable bathochromic shift relative to the absorption of the neutral germylene **1** (λ_max_ = 447 nm) (Figs. S2–S3, S6). Time-dependent DFT (TD-DFT) calculations further support these assignments. For the germanium radical anion **3**, the calculated absorption maximum at 454 nm is mainly attributed to HOMO (β) → LUMO (β) transition. This result is in good agreement with the experimentally observed absorption band.

**Fig. 3:**
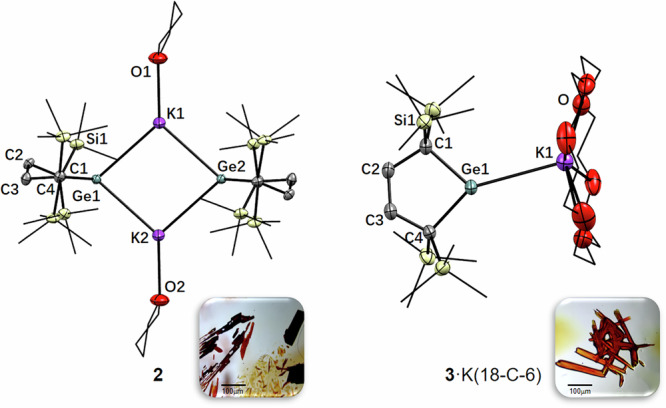
Single-crystal structure of **2** and **3**·K(18-C-6). Molecular structures of **2** (left) and **3**·K(18-C-6) (right) in the solid state. Thermal ellipsoids are shown at the 50% probability level and hydrogen atoms were omitted for clarity.

Graphic representations of the solid-state structures of the Ge(I) radical anions **2** and **3**·K(18-C-6) are depicted in Fig. 3 (for details, see Supplementary Information). Compound **2**, crystallized as a THF solvate, forms a dimeric structure in the solid state featuring two germanium centers bridged by potassium ions (Fig. 3a). The nearly square-planar Ge_2_K_2_ core is oriented perpendicular to the two five-membered rings (GeC_4_). The Ge···K distances in **2** (3.367(12) and 3.405(12) Å) are considerably exceeds the Pyykkö’s covalent bond radius sum for Ge–K bond (3.17 Å)^71^, but fall within the reported range for Ge···K interactions (3.19–3.79 Å)^43,58,72^. A slightly elongated Ge···K distance was observed in **3**·K(18-C-6) (3.557(9) Å), which can be attributed to the coordination of 18-crown-6 (18-C-6) ether to the potassium centers in **3**·K(18-C-6). The Ge–C bond lengths in **2** (2.083(4) and 2.062(4) Å) and **3**·K(18-C-6) (2.111(3) and 2.100(3) Å) are slightly elongated compared to those in the precursor germylene **1** (2.016(5) and 2.014(5) Å), consistent with the expected increase in ionic radius upon formal one-electron reduction from Ge(II) to Ge(I)^70^. Collectively, these structural parameters provide strong evidence for the localization of the unpaired electron at the germanium centers in both anionic species.

**Fig. 4:**
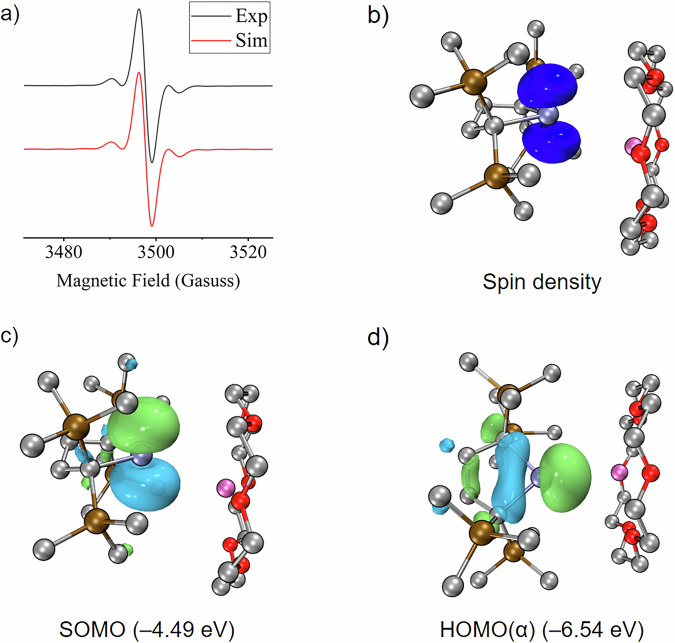
The EPR spectrum, electron spin density distribution and frontier molecular orbitals of **3**·K(18-C-6). **a** The experimental and simulated EPR spectra of **3**·K(18-C-6) in toluene solution (298 K, 9.86 GHz); **b** spin density of **3**·K(18-C-6) (isovalue = 0.005); **c**,**d** calculated frontier molecular orbitals (isovalue = 0.05) of **3**·K(18-C-6) (calculated at the UBP86-D3(BJ)/def2-TZVP level).

The germanium(I) radical anion **3**·K(18-C-6) was also characterized by electron paramagnetic resonance (EPR) spectroscopy (Fig. 4a and Fig. S6). The room-temperature EPR spectrum of **3**·K(18-C-6) in THF displays a central singlet accompanied by two satellite signals arising from hyperfine coupling to the ^29^Si nucleus (I = 1/2) of the TMS substituents, with the hyperfine coupling constants *a*(^29^Si) = 12.87 G and 10.39 G. A magnified view of the spectrum reveals additional three satellite peaks on each side of the central line, which is attributable to hyper-fine interaction with the ^73^Ge nucleus (I = 9/2, 7.8% natural abundance), with *a*(^73^Ge) = 5.08 G (Fig. S6). The remaining satellite peaks of the expected 10-line pattern overlap with the intense central signal and are not resolved. The measured *g*-value of 2.0127 for this Ge(I) radical species falls within the characteristic range reported for germanium-centered radicals^53–55,67,68^. Collectively, the EPR data confirm localization of the unpaired electron predominantly on the germanium center.

To gain deeper insight into the electronic structures of the Ge(I) radical anions, density functional theory (DFT) calculations were performed at the UBP86-D3(BJ)/def2-TZVP level. The optimized geometries show excellent agreement with the single crystal X-ray diffraction structures (see Supplementary Information, Table S3). Frontier molecular orbital (FMO) analysis reveals that the singly occupied molecular orbital (SOMO) is predominantly composed of the Ge 4*p*-orbital, while the highest occupied molecular orbital corresponds to the Ge 4*s*-based lone pair (Fig. 4c, d). Spin density analysis further confirms exclusive localization of the unpaired electron on the germanium center (Fig. 4b). These computational results are fully consistent with the EPR spectroscopic data and provide compelling evidence for the radical character being centered on germanium atom.

### Reactivity studies of the germanium radical anion 2

**Fig. 5:**
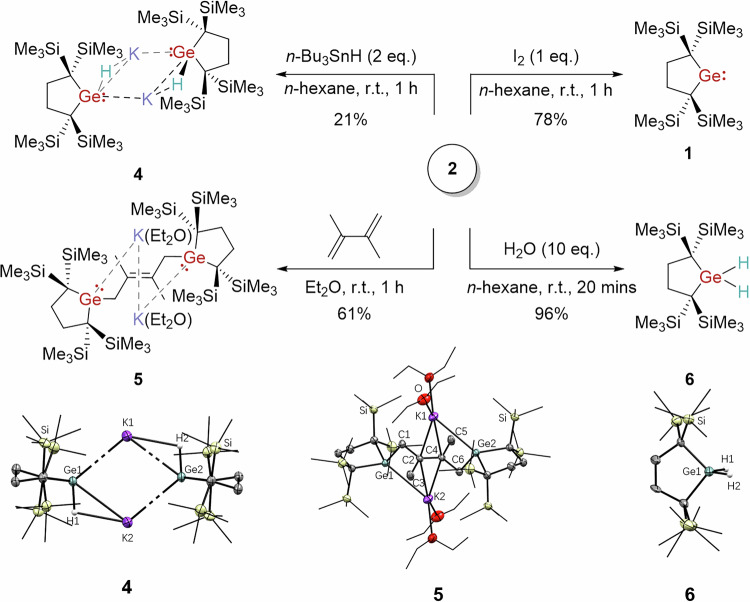
Radical reactivity studies of **2** with *n*-Bu_3_SnH, I_2_, 2,3-dimethyl-1,3-butadiene and H_2_O, respectively. Radical reactivities of **2** and molecular structures of compounds **4,** **5** and **6** in the solid state. Thermal ellipsoids are shown at the 50% probability level and hydrogen atoms were omitted for clarity.

The successful isolation of the germanium radical anion **2** enabled systematic investigation of its reactivity profile (Fig. 5). Initial radical trapping experiments using 2,2,6,6-tetramethylpiperidinooxy (TEMPO) or diphenyl disulfide resulted in complex mixtures, from which no well-defined products could be isolated. In contrast, treatment of compound **2** with *n*-Bu_3_SnH in *n*-hexane at room temperature afforded the hydrogenated product **4** as a colorless solid in 21% yield. The FT-IR spectrum of **4** shows a characteristic absorption band at 1735 cm^−1^, attributable to the Ge–H stretching vibration (Fig. S26)^73^. Furthermore, single-crystal X-ray diffraction (sc-XRD) analysis confirmed its molecular structure, consistent with a formal hydrogen atom transfer to the germanium-centered radical (Fig. 5). Oxidation of **2** with stoichiometric iodine in *n*-hexane cleanly regenerated the parent germylene **1**, establishing the reversibility of the Ge(I) /Ge(II) redox couple and corroborating the electrochemical data. Further reactivity exploration with unsaturated substrates revealed distinct radical pathways. Treatment of **2** with 2,3-dimethyl−1,3-butadiene yielded **5** as colorless crystals in 61% yield. Crystallographic analysis showed the two potassium ions were positioned above and below the central C = C unit, indicating significant cation–*π* interactions within the adduct. The solid-state structure further revealed the formation of Ge–C bonds at the terminal positions of the diene, consistent with a 1,4-addition process and supporting the radical nature of **2**. This reactivity contrasted markedly with that of germylene **1**, which undergoes [4 + 2] cycloaddition with the same diene, underscoring the unique reactivity imparted by the radical anion state^70^. Finally, exposure of **2** to air or treatment with excess H_2_O yielded the hydrogenated product **6** as colorless crystals. To ascertain the origin of the hydrogen atoms, **2** was treated with D_2_O. In the resulting ^1^H NMR spectrum, the Ge–H resonance at 4.62 ppm was absent, consistent with the formation of the corresponding Ge–D isotopologue. These results confirm that both hydrogen atoms in **6** are derived from water (Fig. S21). These comparative studies clearly demonstrate the unique radical reactivity of germanium radical anion.

In summary, we have successfully isolated and fully characterized the alkyl-substituted Ge(I) radical anion, which is an example of an electronically unbiased main group open-shell species stabilized by σ-donating alkyl substituents^74,75^. This radical anion demonstrates remarkable thermal stability in both solid state and solution, enabling detailed structural, spectroscopic, and reactivity investigations. Combined EPR spectroscopy and DFT calculations confirm predominant localization of the unpaired electron at the germanium center. The radical anion exhibits distinct reactivity patterns, including selective hydrogen atom abstraction and unique radical coupling with unsaturated substrates. These findings demonstrate the crucial role of sterically encumbered alkyl ligands in stabilizing open-shell Ge(I) species and establish a new paradigm for exploring other stable low-valent main group radicals.

## Methods

### General considerations

All reactions were performed under a controlled dry argon or nitrogen atmosphere using a high-vacuum line, standard Schlenk techniques, and a Vigor glovebox. The glassware used was dried in an oven at 150 °C and evacuated prior to use. The solvents THF and *n*-hexane were dried over sodium/potassium alloy and distilled under argon prior to use. Deuterated benzene-*d*_6_ and THF-*d*_8_ were dried over sodium/potassium alloy, distilled and stored over molecular sieve (4 Å). All standard chemicals used were obtained from commercial suppliers and used as delivered if not mentioned otherwise. Dialkylgermylene **1** and dichlorogermane **7** were prepared according to the ref. ^70^. For new compounds, all available NMR spectra are provided. NMR spectra were recorded on Bruker AVANCE III HD 400 NMR spectrometers. All NMR spectra were acquired at 298 K, unless otherwise specified. Multiplets were assigned as s (singlet), d (doublet), t (triplet), dd (doublet of doublet) and m (multiplet). ^1^H NMR spectra were calibrated against the residual proton signal of the solvent as internal reference (benzene-*d*_6_: δ^1^H = 7.16 ppm, THF-*d*_8_: δ^1^H = 1.73 ppm, 3.58 ppm) and ^13^C NMR spectra by using the central line of the solvent signal (benzene-*d*_6_: δ^13^C = 128.1 ppm, THF-*d*_8_: δ^13^C = 25.3 ppm, 67.2 ppm). High resolution mass spectra (HRMS) were recorded on the Bruker Daltonics MicroQtof spectrometer equipped with ESI ionization source, THF was used for all samples. Melting points were measured with OptiMelt (Stanford Research Systems). UV-vis spectra (UV-vis) were recorded on a UV−1900i instrument (SHIMADZU). The cyclic voltammetry (CV) experiments were carried out with a CHI 600D electrochemical workstation under an atmosphere of argon at room temperature. The electron paramagnetic resonance (EPR) spectrum in fluid solution at X-band (9.86 GHz) of were measured on a Bruker EMXplus X-band EPR spectrometer. Fourier-transform infrared spectra were obtained using a Brucker ALPHA II.

### Synthesis of compound 2: method 1

THF (20 mL) was added to a mixture of dialkylgermylene **1** (200 mg, 0.48 mmol) and KC_8_ (129 mg, 0.96 mmol) at –30 °C. The reaction mixture was stirred for 16 h, during which the solution gradually turned dark red. Afterward, all volatiles were removed under reduced pressure, and the residue was extracted with *n*-hexane (60 mL). Filtration afforded a dark red solution. Removal of the solvent under vacuum yielded the germanium(I) radical anion **2** as a dark-red solid (84.5 mg, 13%). M.p.: 84.3 °C–84.7 °C (dec.). HRMS (ESI): m/z calculated for C_40_H_96_Ge_2_K_2_O_2_Si_8_ [M + H]^+^ 1057.3344, found 1057.3323.

### Synthesis of compound 2: method 2

THF (50 mL) was added to a mixture of dichlorogermane **7** (300 mg, 0.61 mmol) and KC_8_ (331 mg, 2.44 mmol) at –30 °C. The mixture was stirred for 16 h, during which the solution turned dark red. All volatiles were then removed under reduced pressure, and the remaining residue was extracted with *n*-hexane (80 mL). Filtration afforded a dark red solution. Subsequent removal of the solvent under vacuum gave the germanium(I) radical anion **2** as a dark-red solid (449 mg, 69%).

### Synthesis of compound 3·K(18-C-6): method 1

THF (20 mL) was added to a mixture of **2** (100 mg, 0.14 mmol) and 18-crown-6 (50 mg, 0.28 mmol) at room temperature. The resulting mixture was stirred for 2 h, during which the solution color changed from dark red to orange. After completion, all volatiles were removed under reduced pressure, and the residue was dissolved in diethyl ether (20 mL). Slow evaporation of the solution afforded compound **3**·K(18-C-6) as orange crystals (58 mg, 85%). M.p.: 90.5 °C–91.2 °C(dec.). HRMS (ESI): m/z calculated for C_28_H_64_GeKO_6_Si_4_ [M + H]^+^ 722.2702, found 722.2756.

### Synthesis of compound 3·K(18-C-6): method 2

THF (20 mL) was added to a mixture of **1** (200 mg, 0.48 mmol), KC_8_ (129 mg, 0.96 mmol), and 18-crown-6 (253 mg, 0.96 mmol) at –30 °C. The mixture was stirred for 16 h, during which the solution color changed from dark red to orange. Afterward, the THF was removed under reduced pressure, and the residue was extracted with diethyl ether to give an orange-yellow solution. Removal of the solvent afforded the corresponding product as an orange solid (76 mg, 22%).

### Synthesis of compound 3·K(18-C-6): method 3

THF (50 mL) was added to a mixture of **7** (300 mg, 0.61 mmol), 18-crown-6 (325 mg, 1.22 mmol), and KC_8_ (332 mg, 2.44 mmol) at –30 °C. The mixture was stirred for 16 h, during which the solution color changed from colorless to orange. Afterward, the THF was removed under reduced pressure, and the residue was extracted with diethyl ether to give an orange-yellow solution. Removal of the solvent afforded the corresponding product as an orange solid (155 mg, 35%).

### Synthesis of compound 4

Tri-*n*-butyltin hydride (27.5 mg, 0.94 mmol) was added to an *n*-hexane (10 mL) solution of compound **2** (50 mg, 0.47 mmol) at room temperature. The mixture was stirred for 1 h, during which the solution color changed from deep red to pale yellow and a white precipitate formed. The precipitate was removed by filtration, and the filtrate was concentrated under vacuum. Recrystallization from *n*-pentane at −30 °C afforded the product as colorless crystals (9.1 mg, 21%). M.p.: 76.1 °C–76.8 °C (dec.). ^1^H NMR (400 MHz, THF-*d*_8_): δ = 0.02 (s, 36H, Si*Me*_3_), 0.04 (s, 36H, Si*Me*_3_), 1.87 (d, 8H, qC*H*_2_-C*H*_2_), 3.70 (s, 2H, Ge*H*). ^13^C NMR (101 MHz, THF-*d*_8_): δ = 10.8 (s, TMS-q*C*), 38.3 (s, backbone-q*C*), 3.7 (s, Si-*C*H_*3*_), 3.8 (s, Si-*C*H_*3*_). ^29^Si NMR (99 MHz, THF-*d*_8_): δ = –1.6 (s), 1.1 (s). FT-IR: (solid, cm^−1^): ν_Ge-H_ = 1736. HRMS (ESI): *m/z* calculated for C_32_H_82_Ge_2_K_2_Si_8_ [M+Na]^+^ 937.2277, found 937.2254.

### Synthesis of compound 1

Compound **2** (50 mg, 0.47 mmol) and iodine (12 mg, 0.47 mmol) were added to an *n*-hexane (10 mL) solution at room temperature. The mixture was stirred for 1 h, during which the solution color changed from dark red to orange and a white precipitate formed. The precipitate was removed by filtration, affording an orange solution. Removal of the solvent under vacuum yielded the germylene **1** as a yellow solid (15.6 mg, 78%).^1^H NMR (400 MHz, benzene-d6): δ = 2.39 (s, 4H, C*H*_2_-C*H*_2_), 0.22 (s, 36H, Si*Me*_3_).

### Synthesis of compound 5

2,3-Dimethyl-1,3-butadiene (50 mg, 0.47 mmol) was added to a diethyl ether (5 mL) solution of compound **2** (50 mg, 0.47 mmol) at room temperature. The mixture was stirred for 1 h, during which the solution color changed from dark red to pale yellow. Removal of the diethyl ether under vacuum afforded the product as a yellow solid. Slow evaporation of the solution afforded compound **5** as colorless crystals (37.2 mg, 61%). M.p.: 75.7 °C–76.3 °C (dec.). ^1^H NMR (400 MHz, THF-*d*_8_): δ = 1.94 (s, 8H, qC*H*_2_-C*H*_2_), 0.09-0.10 (d, 72H, Si*Me*_3_), 1.09-1.13 (m, 12H, *J* = 0.2 Hz, OCH_2_C*H*_3_), 3.35–3.41 (m, 8H, *J* = 2.0 Hz, OC*H*_2_CH_3_), 1.74 (s, 6H, CC*H*_3_), 2.13 (s, 4H, CC*H*_2_). ^13^C NMR (101 MHz, THF-*d*_8_): δ = 4.5 (s, Si(*C*H_3_)_3_), 5.5 (s, Si(*C*H_3_)_3_), 15.3 (s, backbone-q*C*), 15.5 (s, C-*C*H_3_), 22.9 (s, OCH_2_*C*H_3_), 66.1 (s, O*C*H_2_CH_3_), 29.0 (s, backbone-q*C*H_2_), 37.1 (s, C*C*H_2_), 128.7 (s, CH_3_*C*CH_2_). ^29^Si NMR (99 MHz, THF-*d*_8_): δ = –0.3 (s), 1.8(s). FT-IR: (solid, cm^-1^): ν_Ge-H_ = 2240, 2087. HRMS (ESI): m/z calculated for C_54_H_130_Ge_2_K_2_Si_8_O_4_ [M + H]^+^ 1292.6555, found 1292.6615.

### Synthesis of compound 6

H_2_O (169.3 μL, 9.4 mmol) was added to the *n*-hexane (5 mL) solution of compound **2** (100 mg, 0.94 mmol) at room temperature. The mixture was stirred for 1 h, during which the solution color changed from dark red to colorless. Removal of the *n*-hexane under vacuum afforded the product as a white solid. Slow evaporation of the solution afforded compound **6** as colorless crystals (38.4 mg, 96%). M.p.: 75.9 °C–76.5 °C (dec.). ^1^H NMR (400 MHz, benzene-*d*_*6*_): δ = 1.83 (s, 4H, C*H*_2_-C*H*_2_), 0.19 (s, 36H, Si*Me*_3_), 4.62 (s, 2H, Ge*H*_2_). ^13^C NMR (101 MHz, benzene-*d*_*6*_): δ = 2.4 (s, TMS-q*C*), 35.0 (s, backbone-q*C*), 7.8 (s, Si-*C*H_*3*_). ^29^Si NMR (99 MHz, benzene-*d*_*6*_): δ = 3.8 (s). FT-IR: (solid, cm^-1^): ν_Ge-H_ = 2043. HRMS (ESI): *m/z* calculated for C_16_H_42_GeSi_4_ [M + H]^+^ 421.1648, found 421.1688.

### Synthesis of compound 7^70^

THF (50 mL) was added to a mixture of lithium granules (580 mg, 83.6 mmol) and trimethyl(1-(trimethylsilyl)vinyl)silane (12 g, 69.6 mmol) at room temperature. The mixture was stirred overnight, during which the solution color changed from colorless to orange. Then, CuCN (7.7 g, 85.5 mmol) was added under ice-water bath conditions. After reacting for 4 h, GeCl_4_ (11.8 g, 55.0 mmol) was added dropwise, and the reaction mixture was stirred overnight. Afterward, the mixture was filtered and the THF was removed under reduced pressure. The residue was extracted with *n*-hexane to give a pale-yellow solution. Removal of the solvent afforded the corresponding crude product. Purification by column chromatography (petroleum ether) followed by recrystallization afforded compound **7** as white crystals (5.1 g, 30%). ^1^H NMR (400 MHz, benzene-*d*_*6*_): δ = 0.28 (s, 36H, Si*Me*_3_), 1.82 (s, 4H, C*H*_2_).

## Supplementary information

Supplementary Information

Transparent Peer Review file

## Source data

Source Data

## Data Availability

General information, experimental details, and analytical data: NMR spectra, HRMS data, UV-vis spectra and computational details can be found in the Supplementary Information. The X-ray crystallographic coordinates for structures reported in this study have been deposited at the Cambridge Crystallographic Data Center (CCDC), under deposition numbers 2513038 (2), 2513039 (3·K(18-C-6)), 2513037 (4), 2513036 (5), 2513035 (6), respectively. These data can be obtained free of charge from The Cambridge Crystallographic Data Center via www.ccdc.cam.ac.uk/data_request/cif. Source Data containing optimized Cartesian coordinates are provided in this paper. All data are available from the corresponding author upon request. Source data are provided with this paper.

## References

[1] Koivisto, B. D. & Hicks, R. G. The magnetochemistry of verdazyl radical-based materials. *Coord. Chem. Rev.* **249**, 2612–2630 (2005).

[2] Hicks, R. *Stable Radicals: Fundamentals and Applied Aspects of Odd-electron Compounds*. (Wiley, 2010).

[3] Ratera, I. & Veciana, J. Playing with organic radicals as building blocks for functional molecular materials. *Chem. Soc. Rev.* **41**, 303–349 (2012). 21850355 10.1039/c1cs15165g

[4] Abe, M. Diradicals. *Chem. Rev.* **113**, 7011–7088 (2013). 23883325 10.1021/cr400056a

[5] Bonanno, N. M. et al. Reversible solution π-dimerization and long multicenter bonding in a stable phenoxyl radical. *Chem. Eur. J.* **24**, 14906–14910 (2018). 30040151 10.1002/chem.201802204

[6] Stuyver, T. et al. Do diradicals behave like radicals? *Chem. Rev.* **119**, 11291–11351 (2019). 31593450 10.1021/acs.chemrev.9b00260

[7] Péter, Á, Agasti, S., Knowles, O., Pye, E. & Procter, D. J. Recent advances in the chemistry of ketyl radicals. *Chem. Soc. Rev.* **50**, 5349–5365 (2021). 33972956 10.1039/d0cs00358a PMC8111543

[8] Bhunia, A. & Studer, A. Recent advances in radical chemistry proceeding through pro-aromatic radicals. *Chem* **7**, 2060–2100 (2021).

[9] Stubbe, J. & Nocera, D. G. Radicals in biology: your life is in their hands. *J. Am. Chem. Soc.* **143**, 13463–13472 (2021). 34423635 10.1021/jacs.1c05952 PMC8735831

[10] Lee, W.-C. C. & Zhang, X. P. Metalloradical catalysis: general approach for controlling reactivity and selectivity of homolytic radical reactions. *Angew. Chem. Int. Ed.* **63**, e202320243 (2024). 10.1002/anie.202320243 PMC11097140 38472114

[11] Mizuno, A., Matsuoka, R., Mibu, T. & Kusamoto, T. Luminescent radicals. *Chem. Rev.* **124**, 1034–1121 (2024). 38230673 10.1021/acs.chemrev.3c00613

[12] Meckes, J. A. et al. Verdazyl-based radicals for high-field dynamic nuclear polarization NMR. *J. Am. Chem. Soc.* **147**, 7293–7304 (2025). 39982131 10.1021/jacs.4c13374

[13] Zeng, L., Zhang, Y., Hu, M., Ouyang, X.-H. & Li, J.-H. Synthetic applications of electro/photochemical halogen atom transfer (XAT)-driven carbon radical chemistry. *Chem. Soc. Rev.* **55**, 955–1007 (2026). 41347475 10.1039/d5cs00849b

[14] Hinz, A., Bresien, J., Breher, F. & Schulz, A. Heteroatom-based diradical(oid)s. *Chem. Rev.* **123**, 10468–10526 (2023). 37556842 10.1021/acs.chemrev.3c00255

[15] Yeo, H., Debnath, S., Krishnan, B. P. & Boudouris, B. W. Radical polymers in optoelectronic and spintronic applications. *RSC Appl. Polym.* **2**, 7–25 (2024).

[16] Tahir, H. et al. Verdazyl radical polymers for advanced organic spintronics. *Nat. Commun.* **16**, 652 (2025). 39809810 10.1038/s41467-025-56056-w PMC11733114

[17] Liu, L. L. & Stephan, D. W. Radicals derived from Lewis acid/base pairs. *Chem. Soc. Rev.* **48**, 3454–3463 (2019). 30724924 10.1039/c8cs00940f

[18] Jupp, A. R. & Stephan, D. W. New directions for frustrated Lewis pair chemistry. *Trends Chem.* **1**, 35–48 (2019).

[19] Hicks, R. G. What’s new in stable radical chemistry? *Org. Biomol. Chem.* **5**, 1321–1338 (2007). 17464399 10.1039/b617142g

[20] Zetterlund, P. B., Kagawa, Y. & Okubo, M. Controlled/living radical polymerization in dispersed systems. *Chem. Rev.* **108**, 3747–3794 (2008). 18729519 10.1021/cr800242x

[21] Sanvito, S. Molecular spintronics. *Chem. Soc. Rev.* **40**, 3336–3355 (2011). 21552606 10.1039/c1cs15047b

[22] Rostro, L., Baradwaj, A. G. & Boudouris, B. W. Controlled radical polymerization and quantification of solid state electrical conductivities of macromolecules bearing pendant stable radical groups. *ACS Appl. Mater. Interfaces* **5**, 9896–9901 (2013). 24044350 10.1021/am403223s

[23] Nesterov, V. et al. NHCs in main group chemistry. *Chem. Rev.* **118**, 9678–9842 (2018). 29969239 10.1021/acs.chemrev.8b00079

[24] Tang, B., Zhao, J., Xu, J.-F. & Zhang, X. Tuning the stability of organic radicals: from covalent approaches to non-covalent approaches. *Chem. Sci.* **11**, 1192–1204 (2020). 34123243 10.1039/c9sc06143f PMC8148027

[25] Hatakeyama-Sato, K. & Oyaizu, K. Redox: organic robust radicals and their polymers for energy conversion/storage devices. *Chem. Rev.* **123**, 11336–11391 (2023). 37695670 10.1021/acs.chemrev.3c00172

[26] Tang, S. & Wang, X. Spin frustration in organic radicals. *Angew. Chem. Int. Ed.* **63**, e202310147 (2024). 10.1002/anie.202310147 37767854

[27] Kim, Y. & Lee, E. Stable organic radicals derived from N-heterocyclic carbenes. *Chem. Eur. J.* **24**, 19110–19121 (2018). 30058298 10.1002/chem.201801560

[28] Kato, K. & Osuka, A. Platforms for stable carbon-centered radicals. *Angew. Chem. Int. Ed.* **58**, 8978–8986 (2019). 10.1002/anie.201900307 30746863

[29] Leifert, D. & Studer, A. The persistent radical effect in organic synthesis. *Angew. Chem. Int. Ed.* **59**, 74–108 (2020). 10.1002/anie.201903726 31116479

[30] Ren, H., Hu, M. & Li, J. Recent advances in carbon-centered radical-initiated olefin transformation chemistry. *Catalysts* **15**, 461 (2025).

[31] Power, P. P. Persistent and stable radicals of the heavier main group elements and related species. *Chem. Rev.* **103**, 789–810 (2003). 12630853 10.1021/cr020406p

[32] Lee, V. Y. & Sekiguchi, A. Stable silyl, germyl, and stannyl cations, radicals, and anions: heavy versions of carbocations, carbon radicals, and carbanions. *Acc. Chem. Res.* **40**, 410–419 (2007). 17487959 10.1021/ar6000473

[33] Lee, V. Y., Nakamoto, M. & Sekiguchi, A. Making stable radicals of heavy elements of groups 14 and 13: the might of silyl substitution. *Chem. Lett.* **37**, 128–133 (2008).

[34] Chandra Mondal, K., Roy, S. & Roesky, H. W. Silicon-based radicals, radical ions, diradicals and diradicaloids. *Chem. Soc. Rev.* **45**, 1080–1111 (2016). 26585359 10.1039/c5cs00739a

[35] Tan, G. & Wang, X. Isolable radical ions of main-group elements: structures, bonding and properties. *Chin. J. Chem.* **36**, 573–586 (2018).

[36] Helling, C. & Schulz, S. Long-lived radicals of the heavier group 15 elements arsenic, antimony, and bismuth. *Eur. J. Inorg. Chem.* **2020**, 3209–3221 (2020).

[37] Taniguchi, T. Advances in chemistry of N-heterocyclic carbene boryl radicals. *Chem. Soc. Rev.* **50**, 8995–9021 (2021). 34250526 10.1039/d1cs00385b

[38] Peng, T.-Y., Zhang, F.-L. & Wang, Y.-F. Lewis base–boryl radicals enabled borylation reactions and selective activation of carbon–heteroatom bonds. *Acc. Chem. Res.* **56**, 169–186 (2023). 36571794 10.1021/acs.accounts.2c00752

[39] Symes, D. L. G. & Masuda, J. D. Recent advances in heavier group 15 (P, As, Sb, Bi) radical chemistry – frameworks, small molecule reactivity, and catalysis. *Dalton Trans.* **54**, 5234–5249 (2025). 40028835 10.1039/d4dt03582h

[40] Dong, Z. et al. Evidence for a single electron shift in a Lewis acid–base reaction. *J. Am. Chem. Soc.* **140**, 15419–15424 (2018). 30359019 10.1021/jacs.8b09214

[41] Merk, A. et al. Single-electron transfer reactions in frustrated and conventional silylium ion/phosphane Lewis pairs. *Angew. Chem. Int. Ed.* **57**, 15267–15271 (2018). 10.1002/anie.201808922 30178534

[42] Kundu, S., Sinhababu, S., Chandrasekhar, V. & Roesky, H. W. Stable cyclic (alkyl)(amino)carbene (cAAC) radicals with main group substituents. *Chem. Sci.* **10**, 4727–4741 (2019). 31160949 10.1039/c9sc01351b PMC6510188

[43] Reinhold, C. R. W., Schmidtmann, M., Tumanskii, B. & Müller, T. Radicals and anions of siloles and germoles. *Chem. Eur. J.* **27**, 12063–12068 (2021). 33978965 10.1002/chem.202101415 PMC8453960

[44] Feng, Z., Tang, S., Su, Y. & Wang, X. Recent advances in stable main group element radicals: preparation and characterization. *Chem. Soc. Rev.* **51**, 5930–5973 (2022). 35770612 10.1039/d2cs00288d

[45] Tan, G. & Ye, S. Isolable pseudo-monocoordinate group 14 to 16 compounds supported by hydrindacenyl ligands. *Acc. Chem. Res.* **59**, 397–410 (2026). 41364468 10.1021/acs.accounts.5c00706

[46] Ishida, S., Iwamoto, T. & Kira, M. Radical anion of isolable dialkylsilylene. *J. Am. Chem. Soc.* **125**, 3212–3213 (2003). 12630866 10.1021/ja028192g

[47] Ebeler, F. et al. Bicyclo[1.1.0]tetragermane-2,4-diide diradicaloid. *Angew. Chem. Int. Ed.* **64**, e202513772 (2025). 10.1002/anie.202513772 PMC12435412 40772647

[48] Inoue, S., Ichinohe, M. & Sekiguchi, A. Isolable silylene anion radical: structural characteristics in the solid state and in solution. *J. Am. Chem. Soc.* **129**, 6096–6097 (2007). 17441724 10.1021/ja0711314

[49] Inoue, S., Ichinohe, M. & Sekiguchi, A. Isolable alkali-metal-substituted silyl radicals (tBu_2_MeSi)_2_SiM (M=Li, Na, K): electronically and sterically accessible planar silyl radicals. *Organometallics* **27**, 1358–1360 (2008).

[50] Egorov, M. P., Jouikov, V. V., Nikolaevskaya, E. N. & Syroeshkin, M. A. Germanium-centered Ion Radicals. In *Organogermanium Compounds: Theory, Experiment, and Applications* (ed. Lee, V. Y.) 507–559 (Wiley, 2023).

[51] Hinz, A. & Breher, F. Germanium-Containing Radicals. In *Organogermanium Compounds: Theory, Experiment, and Applications* (ed. Lee, V. Y.) 339-360 (Wiley, 2023).

[52] Pu, L. et al. Germanium and tin analogues of alkynes and their reduction products. *J. Am. Chem. Soc.* **125**, 11626–11636 (2003). 13129367 10.1021/ja035711m

[53] Woodul, W. D. et al. A neutral, monomeric germanium(I) radical. *J. Am. Chem. Soc.* **133**, 10074–10077 (2011). 21662245 10.1021/ja204344e

[54] Siddiqui, M. M. et al. Isolation of transient acyclic germanium(I) radicals stabilized by cyclic alkyl(amino) carbenes. *J. Am. Chem. Soc.* **141**, 1908–1912 (2019). 30633503 10.1021/jacs.8b13434

[55] Wang, D. et al. An isolable germylyne radical with a one-coordinate germanium atom. *Nat. Chem.* **15**, 200–205 (2023). 36344822 10.1038/s41557-022-01081-1

[56] Yu, J. et al. Types of six-membered N-heterocyclic germanium radicals: a combined computational and experimental study. *Inorg. Chem.* **58**, 5688–5694 (2019). 30950606 10.1021/acs.inorgchem.9b00034

[57] Sharma, M. K. et al. Isolation of a Ge(I) diradicaloid and dihydrogen splitting. *J. Am. Chem. Soc.* **143**, 121–125 (2021). 33373236 10.1021/jacs.0c11828

[58] Gendy, C., Mikko Rautiainen, J., Mailman, A. & Tuononen, H. M. Low-valent germanylidene anions: efficient single-site nucleophiles for activation of small molecules. *Chem. Eur. J.* **27**, 14405–14409 (2021). 34403540 10.1002/chem.202102804 PMC8596740

[59] Chia, S.-P., Carter, E., Xi, H.-W., Li, Y. & So, C.-W. Group II metal complexes of the germylidendiide dianion radical and germylidenide anion. *Angew. Chem. Int. Ed.* **53**, 8455–8458 (2014). 10.1002/anie.201404357 24924768

[60] M. Olmstead, M., Pu, L., S. Simons, R. & P. Power, P. Reduction of Ge(Cl)C_6_H_3_mes_2_-2,6 to give the cyclotrigermenyl radical (GeC_6_H_3_mes_2_-2,6)_3_· and the trigermenyl anion salt K(GeC_6_H_3_mes_2_-2,6)_3_. *Chem. Commun*. 1595-1596 (Royal Society of Chemistry, 1997).

[61] Sekiguchi, A., Fukawa, T., Nakamoto, M., Lee, V. Y. & Ichinohe, M. Isolable silyl and germyl radicals lacking conjugation with π-bonds: synthesis, characterization, and reactivity. *J. Am. Chem. Soc.* **124**, 9865–9869 (2002). 12175246 10.1021/ja0126780

[62] Drost, C., Griebel, J., Kirmse, R., Lönnecke, P. & Reinhold, J. A stable and crystalline triarylgermyl radical: structure and EPR spectra. *Angew. Chem. Int. Ed.* **48**, 1962–1965 (2009). 10.1002/anie.200805328 19191360

[63] Goldshtein, Y. et al. Synthesis of genuine germenyl lithiums and the first persistent germenyl radicals. *Angew. Chem. Int. Ed.* **61**, e202202452 (2022). 10.1002/anie.202202452 PMC9320928 35438228

[64] Cui, C., Brynda, M., Olmstead, M. M. & Power, P. P. Synthesis and characterization of the Non-Kekulé, Singlet Biradicaloid Ar’Ge(μ-NSiMe_3_)_2_GeAr’(Ar =2,6-Dipp_2_C_6_H_3_, Dipp = 2,6-*i*-Pr_2_C_6_H_3_). *J. Am. Chem. Soc.* **126**, 6510–6511 (2004). 15161252 10.1021/ja0492182

[65] Lee, V. Y. et al. From a (Silatrigerma)cyclobutenylium Ion to a (Silatrigerma)cyclobutenyl Radical and Back. *J. Am. Chem. Soc.* **142**, 16455–16460 (2020). 32862647 10.1021/jacs.0c08052

[66] Dai, Y. et al. Crystalline germanium-dipyrromethene radicals: from a delocalized neutral to a localized cation. *Chin. J. Chem.* **40**, 2387–2392 (2022).

[67] Egorov, M. P., Nefedov, O. M., Lin, T.-S. & Gaspar, P. P. Germylene and stannylene anion-radicals: generation and electronic structure. *Organometallics* **14**, 1539–1541 (1995).

[68] Lim, L. F. et al. Crystalline germanium(I) and Tin(I) centered radical anions. *Angew. Chem. Int. Ed.* **61**, e202201248 (2022). 10.1002/anie.202201248 PMC9401049 35266609

[69] Lee, V. Y. *Organogermanium Compounds: Theory, Experiment, and Applications*. (Wiley, 2023).

[70] Kira, M. et al. Synthesis and structure of a stable cyclic dialkylgermylene. *Chem. Lett.* **28**, 263–264 (1999).

[71] Pyykkö, P. Additive covalent radii for single-, double-, and triple-bonded molecules and tetrahedrally bonded crystals: a summary. *J. Phys. Chem. A.* **119**, 2326–2337 (2015). 25162610 10.1021/jp5065819

[72] Siddiqui, M. M. et al. Silanylidene and germanylidene anions: valence-isoelectronic species to the well-studied phosphinidene. *Angew. Chem. Int. Ed.* **57**, 11776–11780 (2018). 10.1002/anie.201805936 29975006

[73] Hadlington, T. J., Driess, M. & Jones, C. Low-valent group 14 element hydride chemistry: towards catalysis. *Chem. Soc. Rev.* **47**, 4176–4197 (2018). 29666847 10.1039/c7cs00649g

[74] Ishida, S., Hirakawa, F. & Iwamoto, T. A stable dialkylphosphinyl radical. *J. Am. Chem. Soc.* **133**, 12968–12971 (2011). 21776957 10.1021/ja205001m

[75] Ishida, S., Hirakawa, F., Furukawa, K., Yoza, K. & Iwamoto, T. Persistent antimony- and bismuth-centered radicals in solution. *Angew. Chem. Int. Ed.* **53**, 11172–11176 (2014). 10.1002/anie.201405509 25066471

