# Supplementary material for: Isolation and reactivity of an alkyl-substituted germanium(I) radical anion
Source: Nat Commun. 2026 Jun 19;17:8251. doi: 10.1038/s41467-026-74699-1 (PMC13462986; doi:10.1038/s41467-026-74699-1)
Supplement: Supplementary file 1 [file 41467_2026_74699_MOESM1_ESM.pdf]

Supplementary Information for

Isolation and Reactivity of an Alkyl-substituted Germanium(I) Radical Anion

Chenting Yan,[1]* Yanxue Bi,[1] Nanfeng Zhou,[1] Yingying Xi,[1] Chanyu Li,[1] Gaofeng Bian,[1] Ru-De Lin,[2] Yihan Zhou,[2] Hanjiao Chen,[3] Zhifang Li[1]*, Zhaowen Dong[2]*

[1] Key Laboratory of Organosilicon Chemistry and Material Technology, Ministry of Education, Zhejiang Key Laboratory of Organosilicon Material Technology, College of Material, Chemistry and Chemical Engineering, Hangzhou Normal University, Hangzhou, 311121, Zhejiang, China

[2] Key Laboratory of Green Chemistry & Technology, Ministry of Education, College of Chemistry, Sichuan University, 610064 Chengdu, People's Republic of China

[3] Analytical & Testing Center, Sichuan University, 610064 Chengdu, People's Republic of China

*Corresponding author: yanchenting@hznu.edu.cn; zhifanglee@hznu.edu.cn; dongzhaowen@scu.edu.cn

Content

1. Supplementary Methods 2
2. Synthesis and Characterization Data 3
    2.1 Synthesis of compound 2 3
    2.2 Synthesis of compound 3·K(18-C-6) 4
3. Cyclic voltammetry measurements 6
4. UV-Vis spectroscopy 7
5. Electron paramagnetic resonance 11
6. Reactivity of compound 2 12
    6.1 The reaction of compound 2 with tri-*n*-butyltin hydride 12
    6.2 The reaction of compound 2 with iodine 16
    6.3 The reaction of compound 2 with 2,3-Dimethyl-1,3-butadiene 17
    6.4 The reaction of compound 2 with water 20
    6.5 The reaction of compound 2 with Deuterated water 23
7. Fourier-transform infrared spectroscopy 24
8. X-ray Crystallographic Studies 26
9. Computational Details 32
10. Supplementary Reference 37

1. Supplementary Methods

All reactions were performed under a controlled dry argon or nitrogen atmosphere using a high-vacuum line, standard Schlenk techniques, and a Vigor glovebox. The glassware used was dried in an oven at 150 °C and evacuated prior to use. The solvents THF and *n*-hexane were dried over sodium/potassium alloy and distilled under argon prior to use. Deuterated benzene-d_6 and THF-d_8 were dried over sodium/potassium alloy, distilled and stored over molecular sieve (4 Å). All standard chemicals used were obtained from commercial suppliers and used as delivered if not mentioned otherwise. Compounds **1** and **7** were prepared according to the reference.[1] For new compounds, all available NMR spectra are provided.

NMR spectroscopy. NMR spectra were recorded on Bruker AVANCE III HD 400 NMR spectrometers. All NMR spectra were acquired at 298 K, unless otherwise specified. Multiplets were assigned as s (singlet), d (doublet), t (triplet), dd (doublet of doublet) and m (multiplet). ^{1}H NMR spectra were calibrated against the residual proton signal of the solvent as internal reference (benzene-d_6: δ^1H = 7.16 ppm, THF-d_8: δ^1H = 1.73 ppm, 3.58 ppm) and ^{13}C NMR spectra by using the central line of the solvent signal (benzene-d_6: δ^{13}C = 128.1 ppm, THF-d_8: δ^{13}C = 25.3 ppm, 67.2 ppm).

HRMS: High resolution mass spectra (HRMS) were recorded on the Bruker Daltonics MicroQtof spectrometer equipped with ESI ionization source, THF was used for all samples.

MS: Melting points were measured with OptiMelt (Stanford Research Systems).

UV-vis spectroscopy: UV-vis spectra (UV-vis) were recorded on a UV-1900i instrument (SHIMADZU).

Cyclic voltammetry: The cyclic voltammetry (CV) experiments were carried out with a CHI 600D electrochemical workstation under an atmosphere of argon at room temperature.

Electron paramagnetic resonance: The electron paramagnetic resonance (EPR) spectrum in fluid solution at X-band (9.86 GHz) of were measured on a Bruker EMXplus X-band EPR spectrometer.

Fourier-transform infrared spectroscopy (FT-IR): Fourier-transform infrared spectra were obtained using a Brucker ALPHA II.

2. Synthesis and Characterization Data

2.1 Synthesis of compound 2

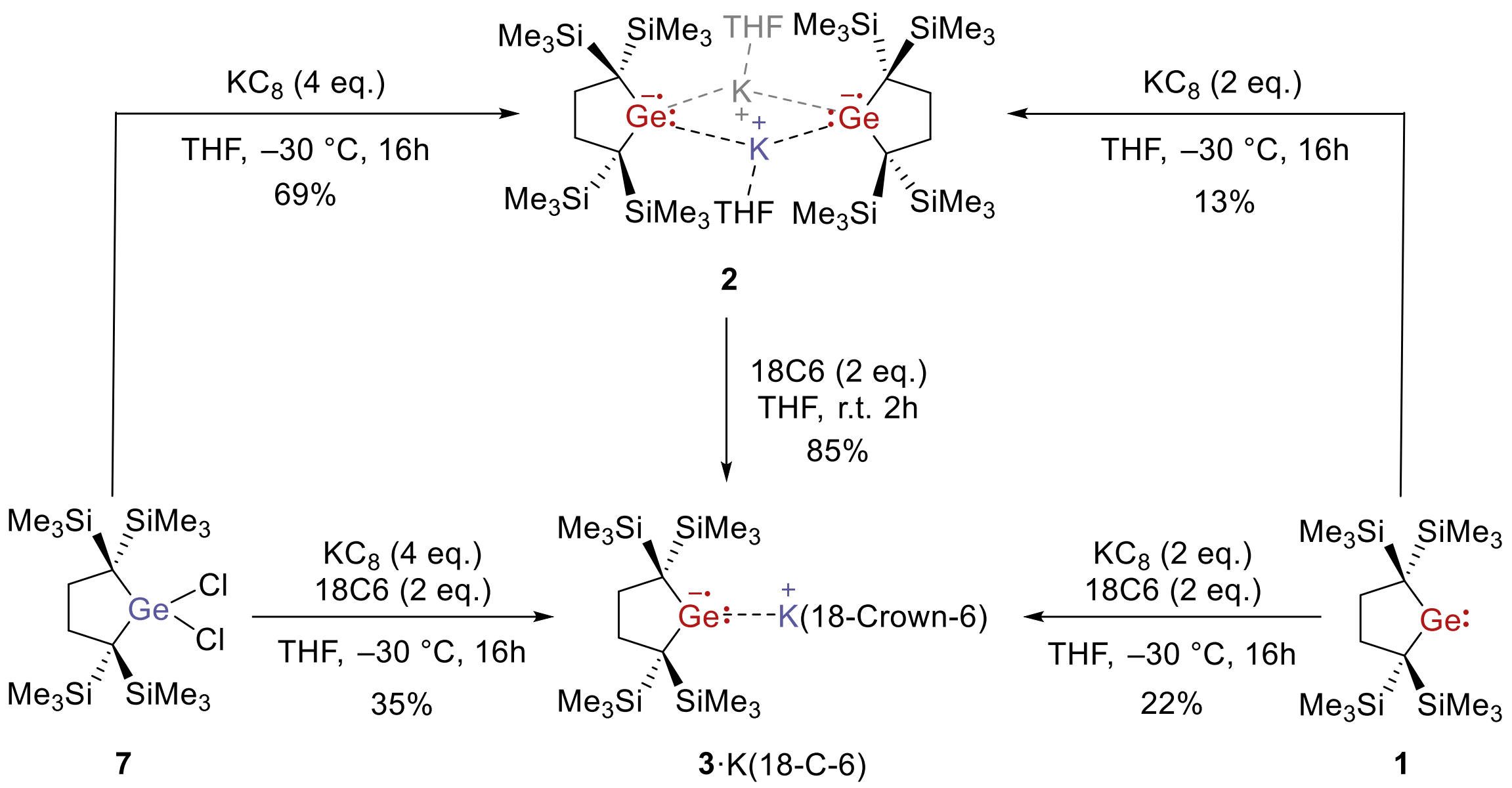

2.1.1 Method 1

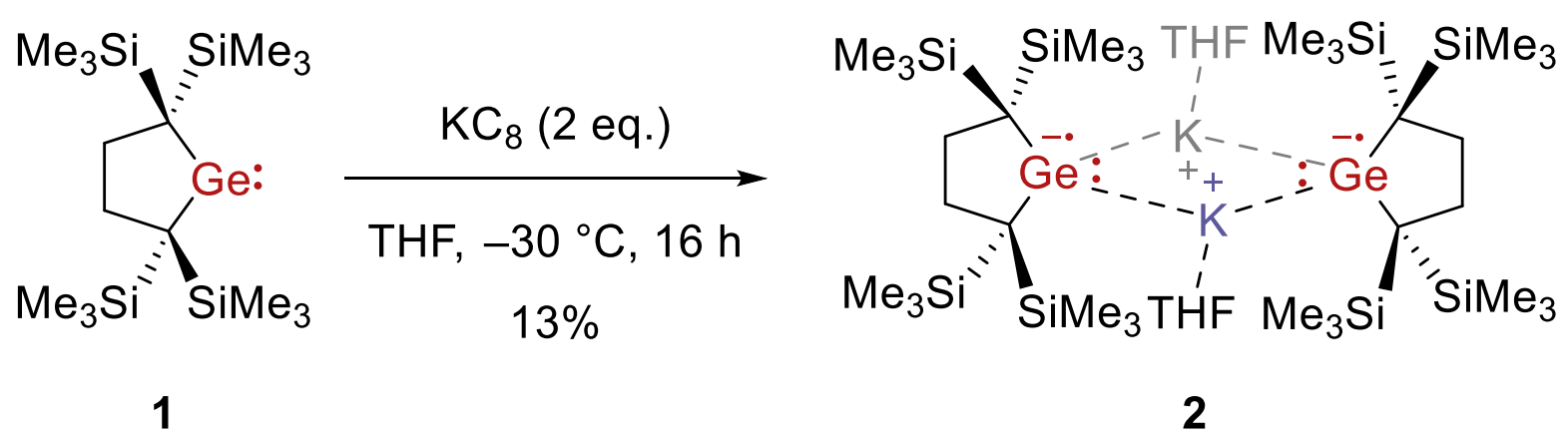

THF (20 mL) was added to a mixture of dialkylgermylene **1** (200 mg, 0.48 mmol) and KC_8 (129 mg, 0.96 mmol) at –30 °C. The reaction mixture was stirred for 16 h, during which the solution gradually turned dark red. Afterward, all volatiles were removed under reduced pressure, and the residue was extracted with *n*-hexane (60 mL). Filtration afforded a dark red solution. Removal of the solvent under vacuum yielded the germanium(I) radical anion **2** as a dark-red solid (84.5 mg, 13%).

M.p.: 84.3 – 84.7 °C (dec.).

HRMS (ESI): m/z calculated for $C_{40}H_{96}Ge_2K_2O_2Si_8$ $[M+H]^+$ 1057.3344, found 1057.3323.

2.1.2 Method 2

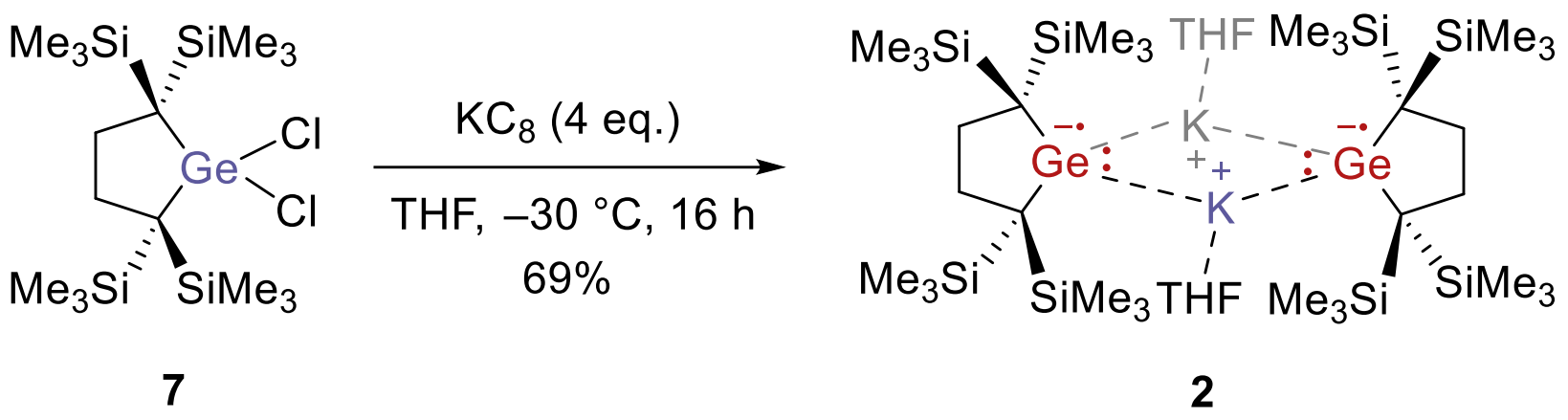

THF (50 mL) was added to a mixture of compound **7** (300 mg, 0.61 mmol) and KC_8 (331 mg, 2.44 mmol) at –30 °C. The mixture was stirred for 16 h, during which the solution turned dark red. All volatiles were then removed under reduced pressure, and the remaining residue was extracted with *n*-hexane (80 mL). Filtration afforded a dark red solution. Subsequent removal of the solvent under vacuum gave the germanium(I) radical anion **2** as a dark-red solid (449 mg, 69%).

2.2 Synthesis of compound 3·K(18-C-6)

2.2.1 Method 1

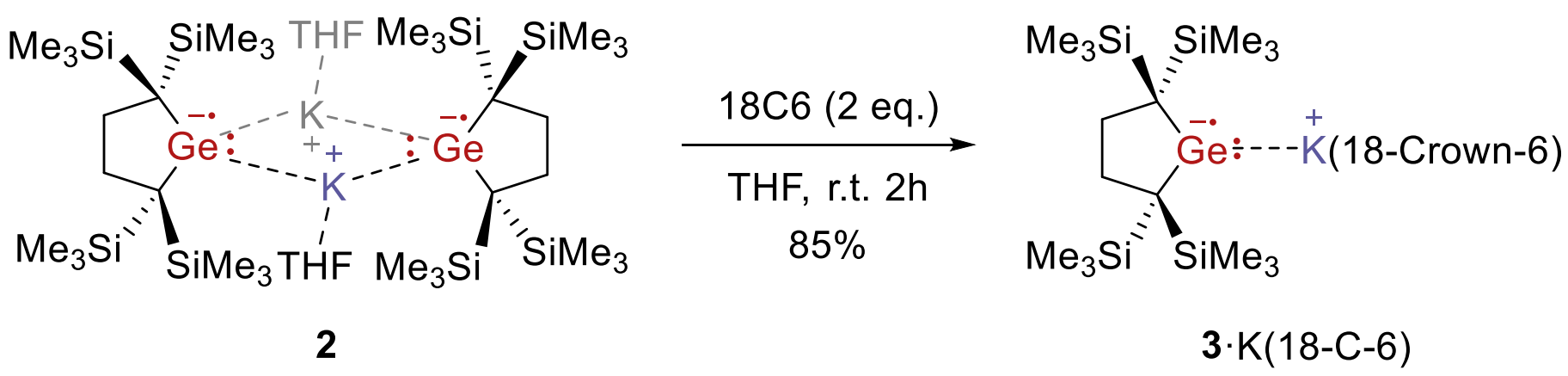

THF (20 mL) was added to a mixture of **2** (100 mg, 0.14 mmol) and 18-crown-6 (50 mg, 0.28 mmol) at room temperature. The resulting mixture was stirred for 2 h, during which the solution color changed from dark red to orange. After completion, all volatiles were removed under reduced pressure, and the residue was dissolved in diethyl ether (20 mL). Slow evaporation of the solution afforded compound **3**·K(18-C-6) as orange crystals (58 mg, 85%).

M.p.: 90.5 – 91.2 °C(dec.).

HRMS (ESI): *m/z* calculated for $C_{28}H_{64}GeKO_6Si_4$ $[M+H]^+$ 722.2702, found 722.2756.

2.2.2 Method 2

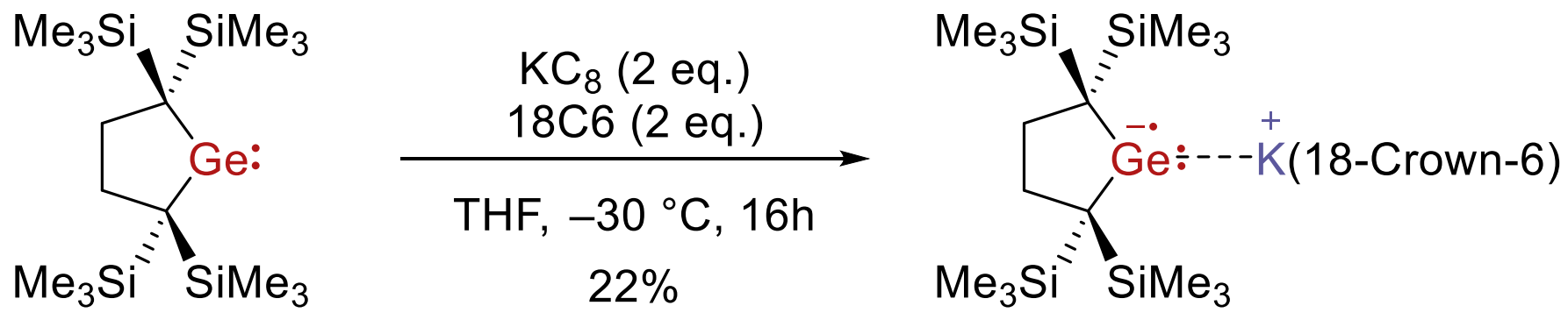

1 **3**·K(18-C-6)

THF (20 mL) was added to a mixture of **1** (200 mg, 0.48 mmol), KC_8 (129 mg, 0.96 mmol), and 18-crown-6 (253 mg, 0.96 mmol) at –30 °C. The mixture was stirred for 16 h, during which the solution changed from dark red to orange. Afterward, the THF was removed under reduced pressure, and the residue was extracted with diethyl ether to give an orange-yellow solution. Removal of the solvent afforded the corresponding product as an orange solid (76 mg, 22%).

2.2.3 Method 3

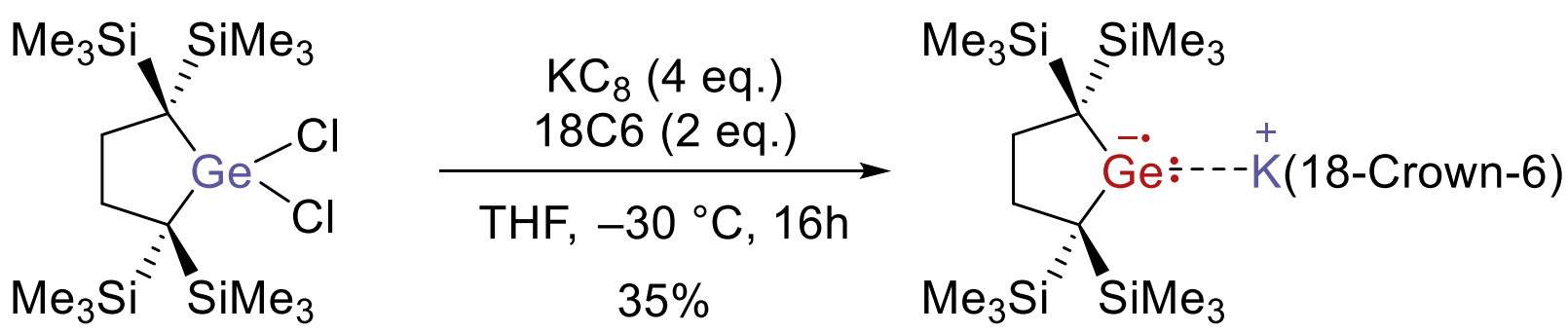

7 **3**·K(18-C-6)

THF (50 mL) was added to a mixture of compound **7** (300 mg, 0.61 mmol), 18-crown-6 (325 mg, 1.22 mmol), and KC_8 (332 mg, 2.44 mmol) at –30 °C. The mixture was stirred for 16 h, during which the solution color changed from colorless to orange. Afterward, the THF was removed under reduced pressure, and the residue was extracted with diethyl ether to give an orange-yellow solution. Removal of the solvent afforded the corresponding product as an orange solid (155 mg, 35%).

3. Cyclic voltammetry measurements

The experiments were carried out at room temperature in THF solution containing [^{n}Bu$_4$N][PF$_6$] (0.1 M) at a scan rate of 0.01 V s^{-1}. The setup consisted of a glassy carbon as working electrode (surface area = 0.06 cm^2), a platinum wire as the counter electrode, and a silver nitrate/silver wire immersed (0.01 M $AgNO_3$ in dry acetonitrile). The CV data have been referenced to the external standard Fc/Fc$^+$ (ferrocene/ferrocenium) couple which was measured under same condition before and after the measurement of samples.

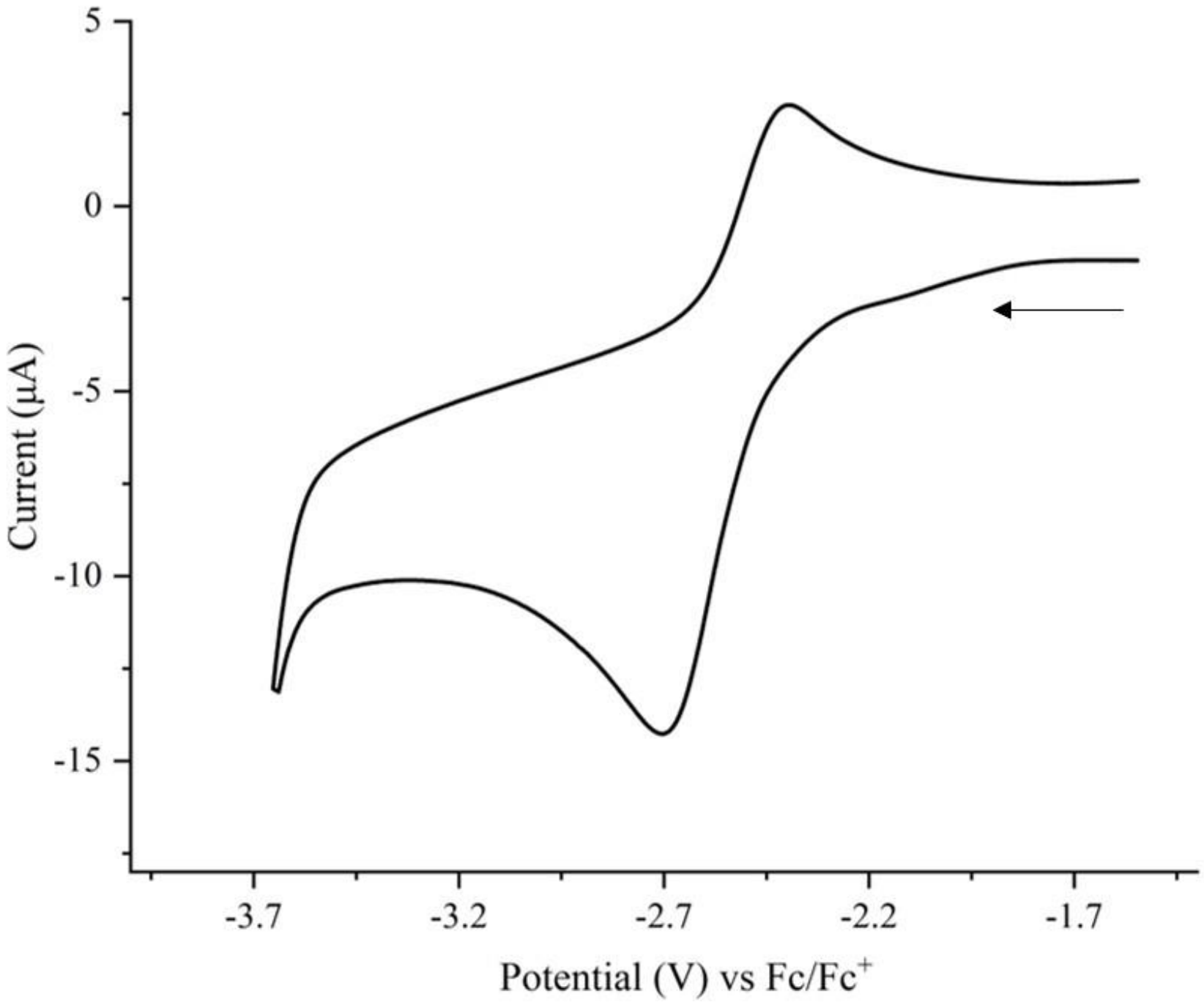

Fig. S1. Cyclic voltammogram of germylene **1** in THF / 0.1 M [*n*-Bu$_4$N][PF$_6$].

4. UV-Vis spectroscopy

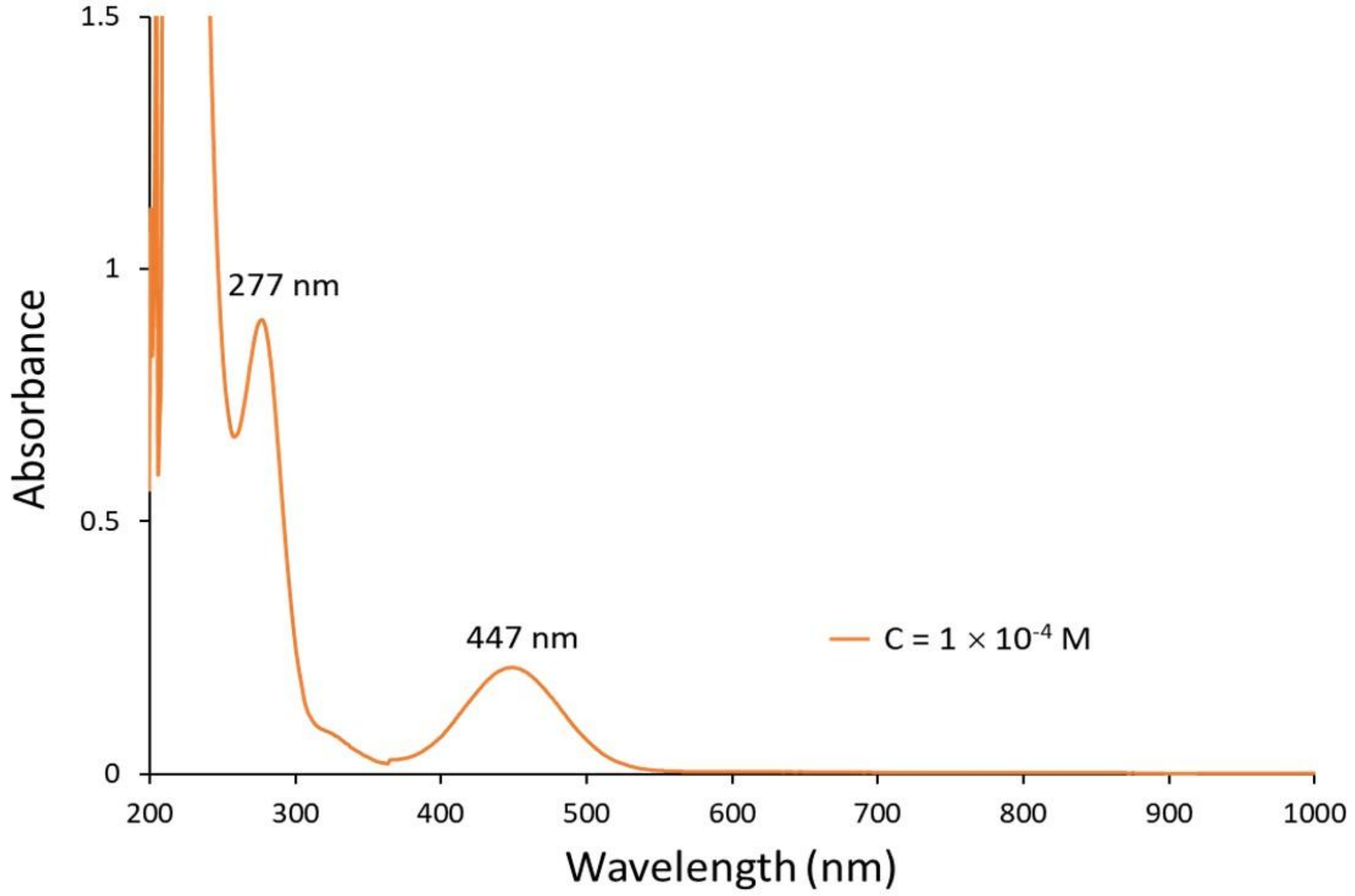

Fig. S2. UV-vis spectrum of compound **1** in THF at 298 K.

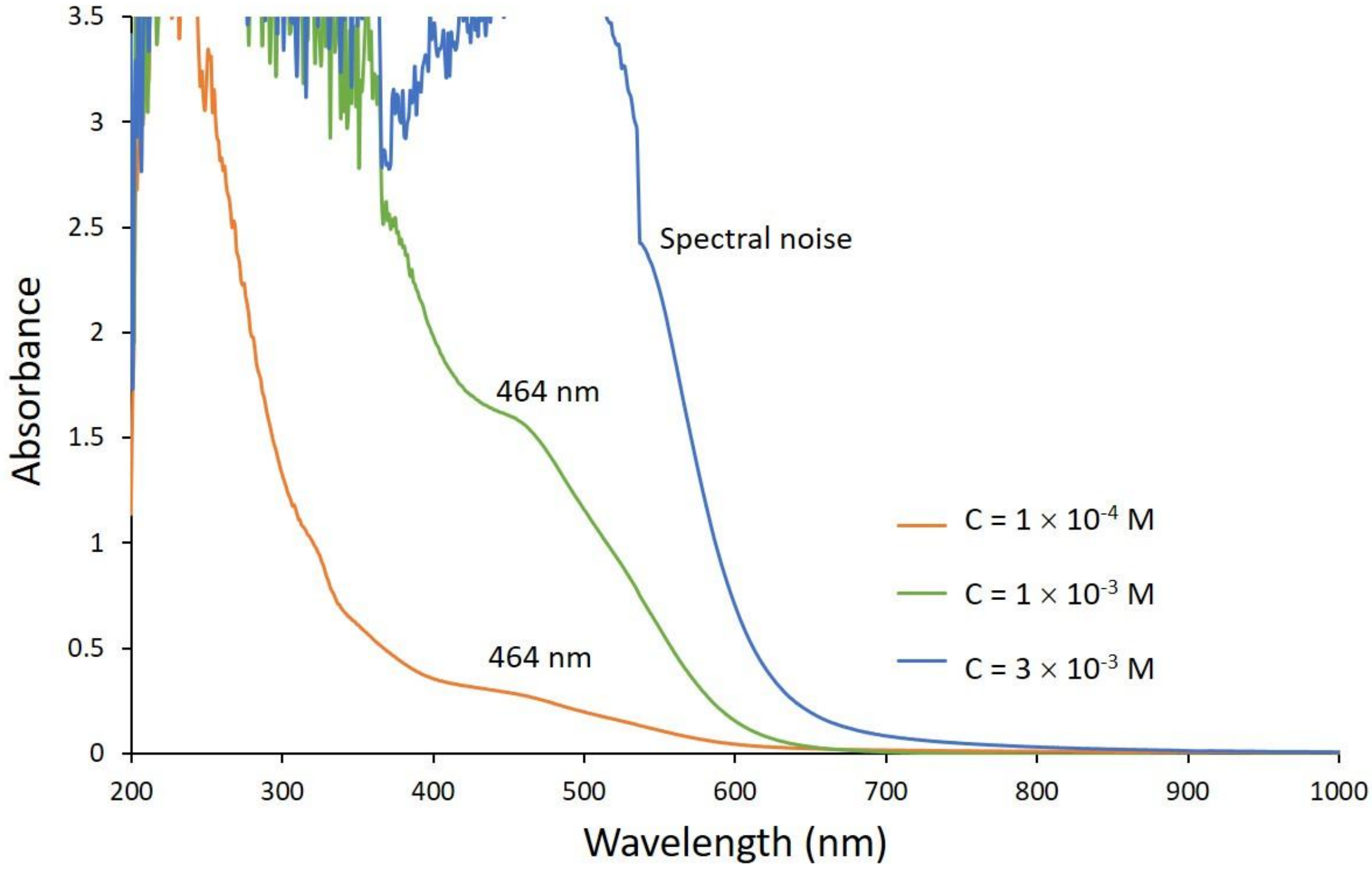

Fig. S3. UV-Vis spectra of germanium radical **2** in THF at different concentrations at 298 K.

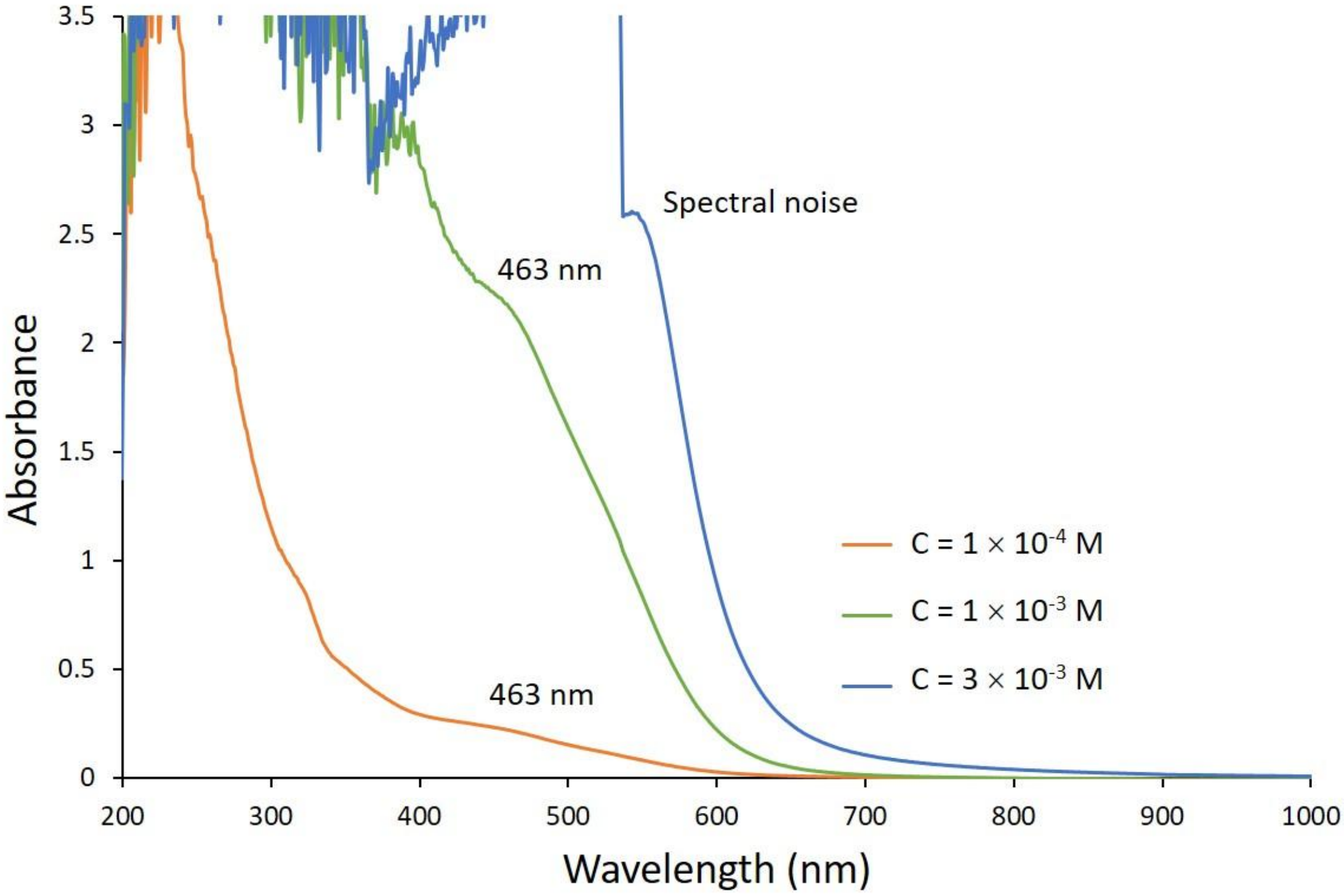

Fig. S4. UV-Vis spectra of germanium radical **2** in Et_2O at different concentrations at 298 K.

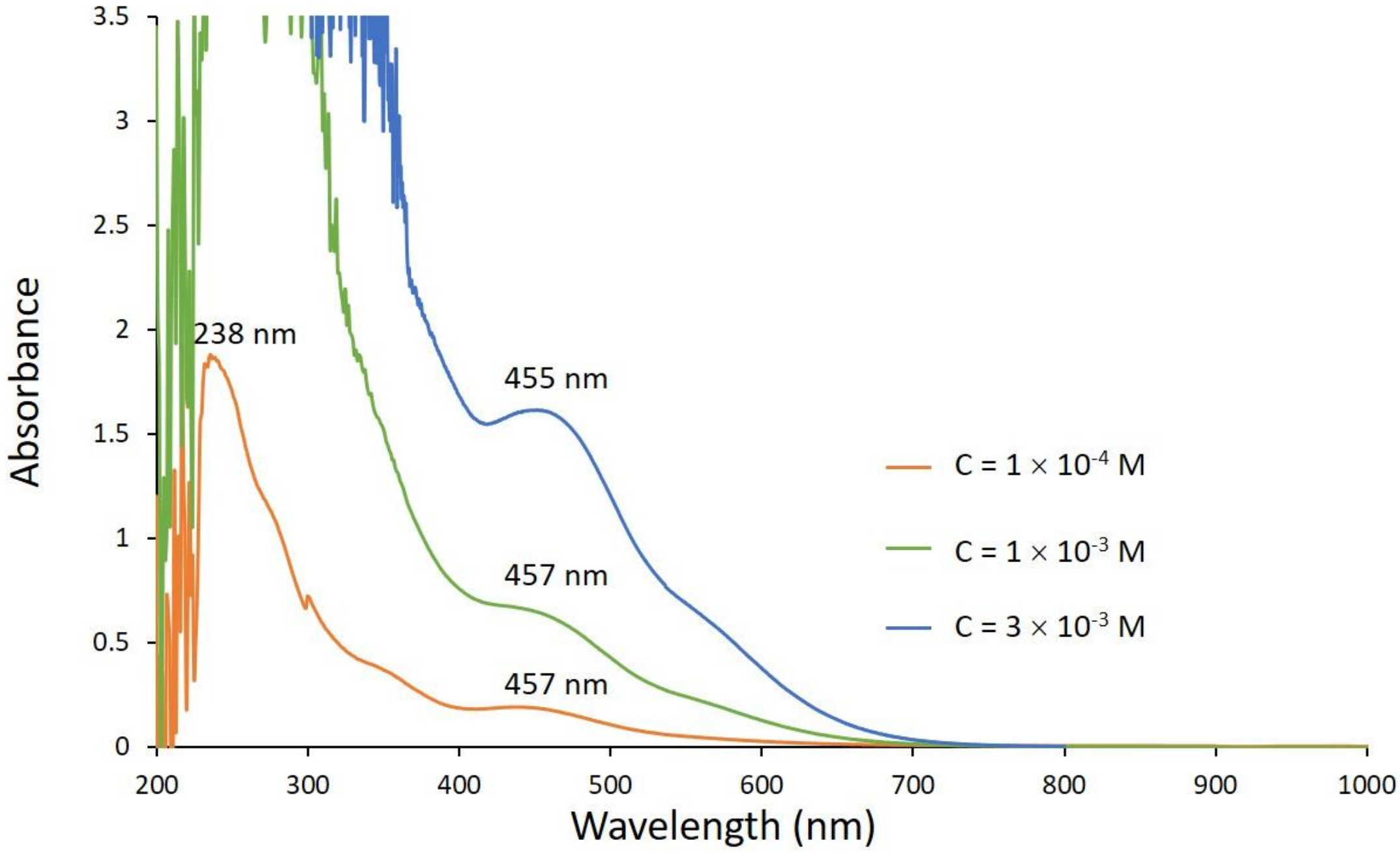

Fig. S5. UV-Vis spectra of germanium radical **2** in *n*-hexane at different concentrations at 298 K.

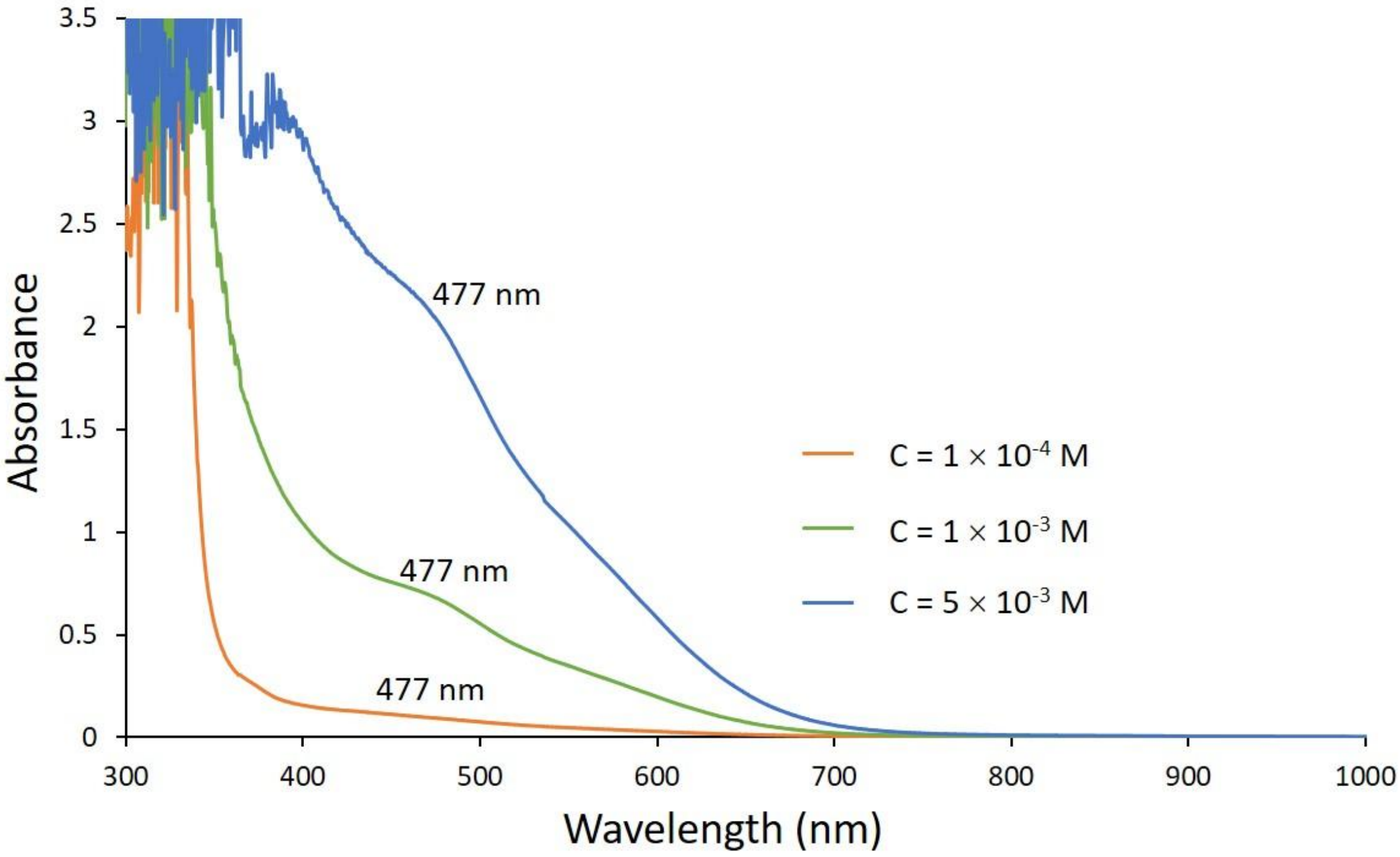

Fig. S6. UV-Vis spectra of germanium radical **3**·K(18-C-6) in THF at different concentrations at 298 K.

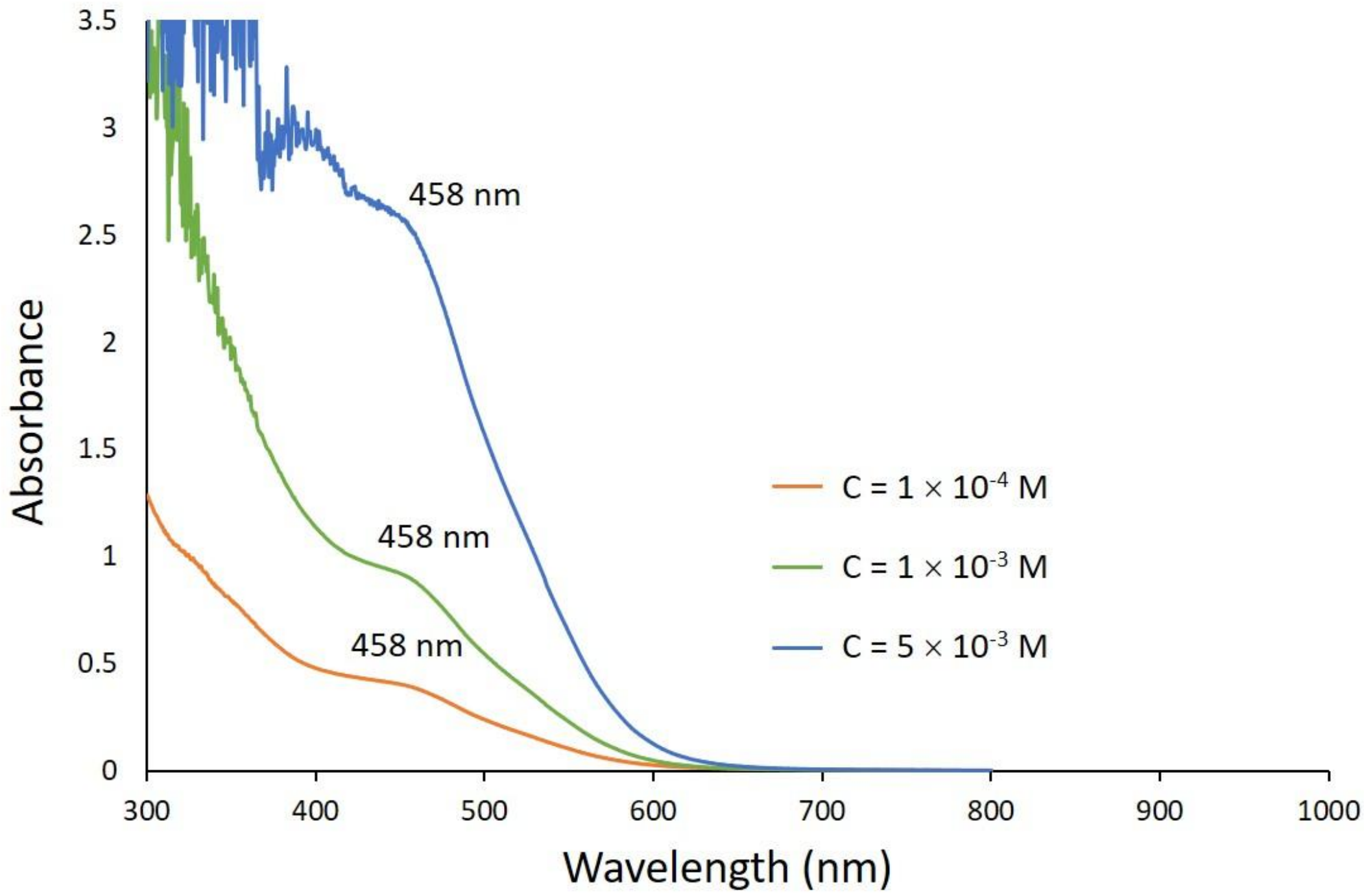

Fig. S7. UV-Vis spectra of germanium radical **3**·K(18-C-6) in Et_2O at different concentrations at 298 K.

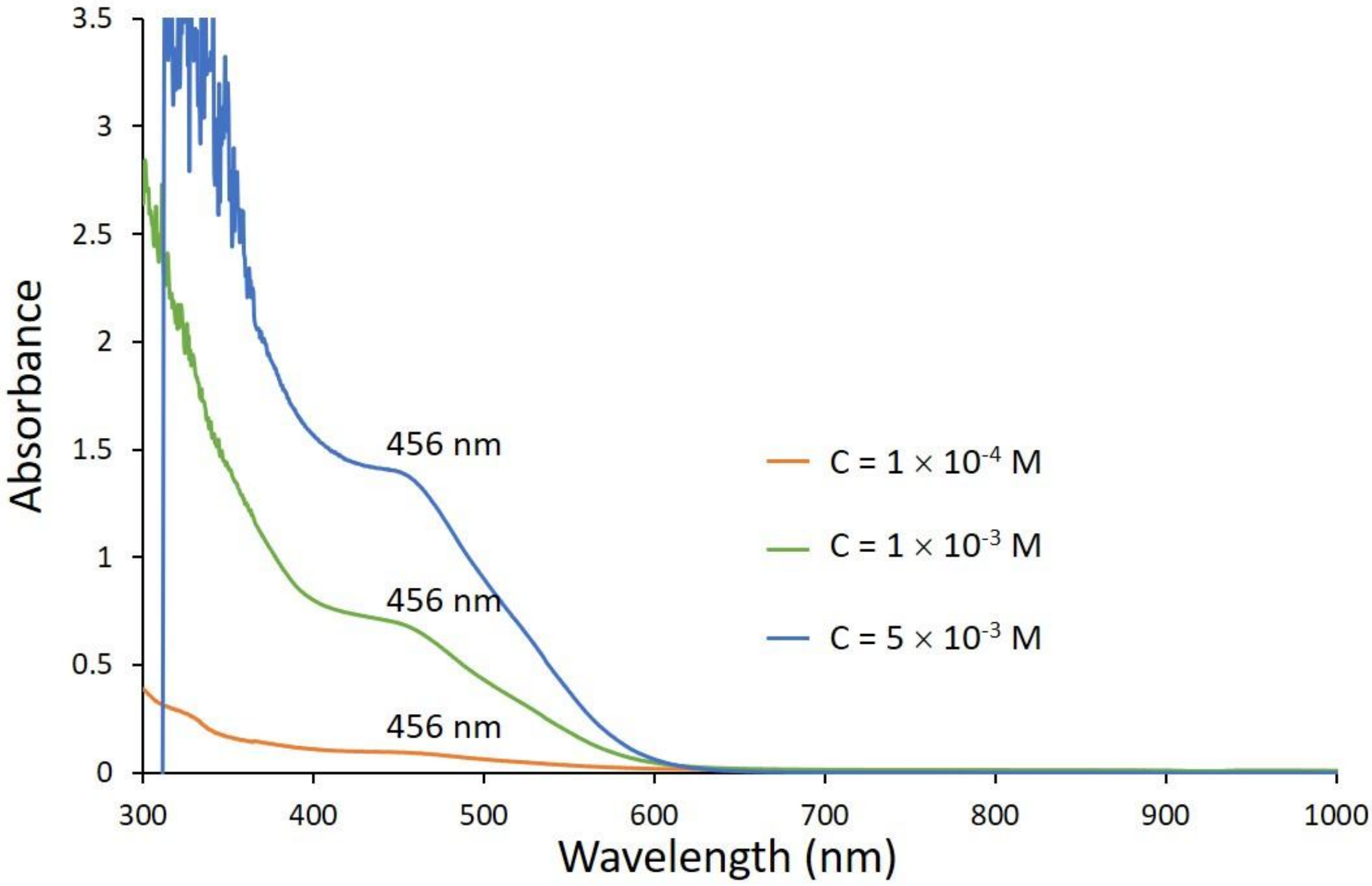

Fig. S8. UV-Vis spectra of germanium radical **3**·K(18-C-6) in *n*-hexane at different concentrations at 298 K.

5. Electron paramagnetic resonance

In the glovebox, the toluene solution of germanium radical **2** was partially transferred into the EPR sample tube and the EPR data were collected at 298 K. The EPR spectrum can be simulated by applying the following parameters: g = 2.0135, $a(^{29}Si)$ = 12.38 G, $a(^{29}Si)$ = 10.56 G, $a(^{73}Ge)$ = 6.32 G.

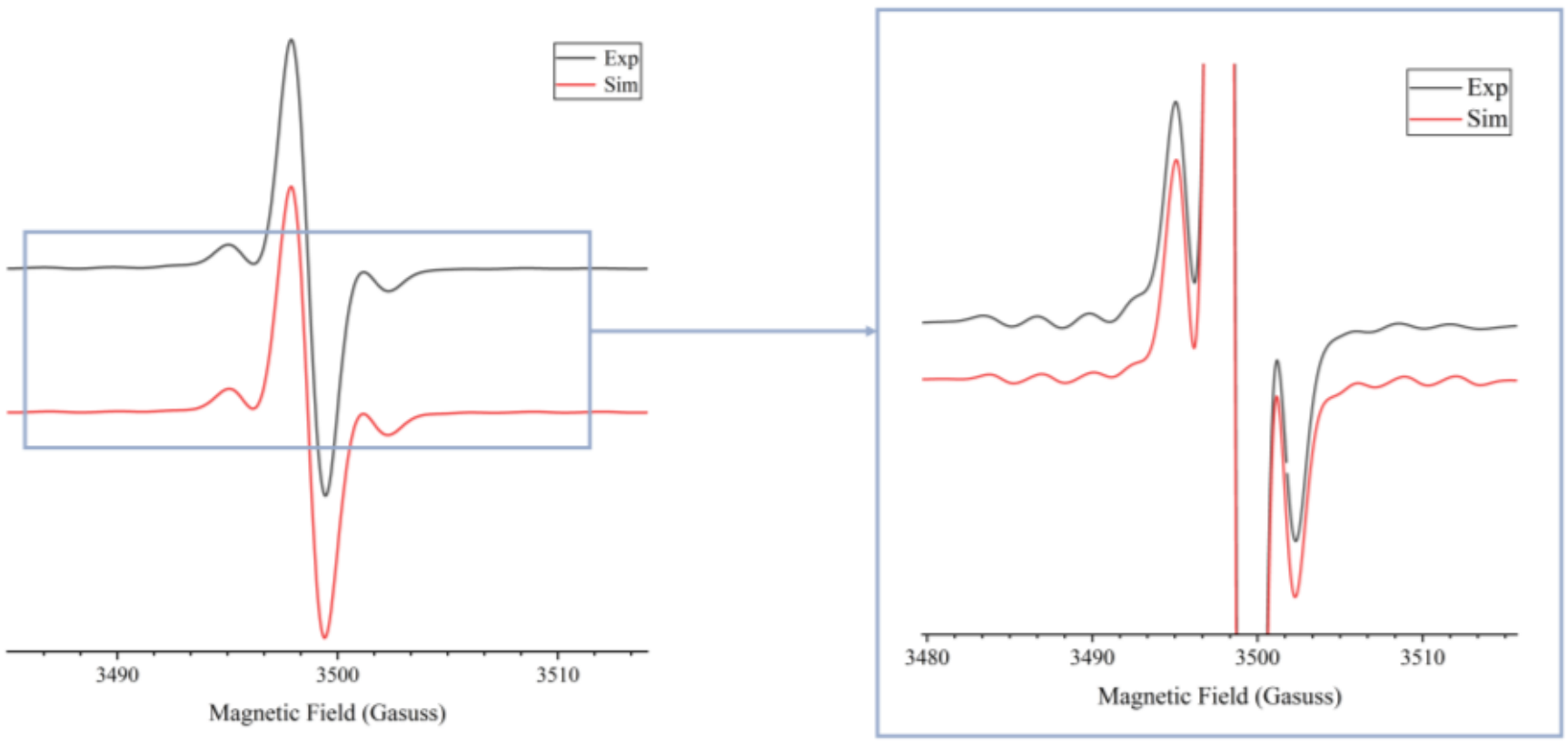

Fig. S9. The experimental (black) and simulated (red) EPR spectra of germanium radical **2** in toluene solution at 298 K.

In the glovebox, the toluene solution of germanium radical **3**·K(18-C-6) was partially transferred into the EPR sample tube and the EPR data were collected at 298 K. The EPR spectrum can be simulated by applying the following parameters: g = 2.0127, $a(^{29}Si)$ = 12.87 G, $a(^{29}Si)$ = 10.39 G, $a(^{73}Ge)$ = 5.08 G. The calculated results for electron-spin g factor and hyperfine coupling constants of **3**·K(18-C-6) show the following parameters: g = 2.0129, $a(^{29}Si)$ = 13.24 G, $a(^{29}Si)$ = 11.12 G, $a(^{73}Ge)$ = 4.78 G.

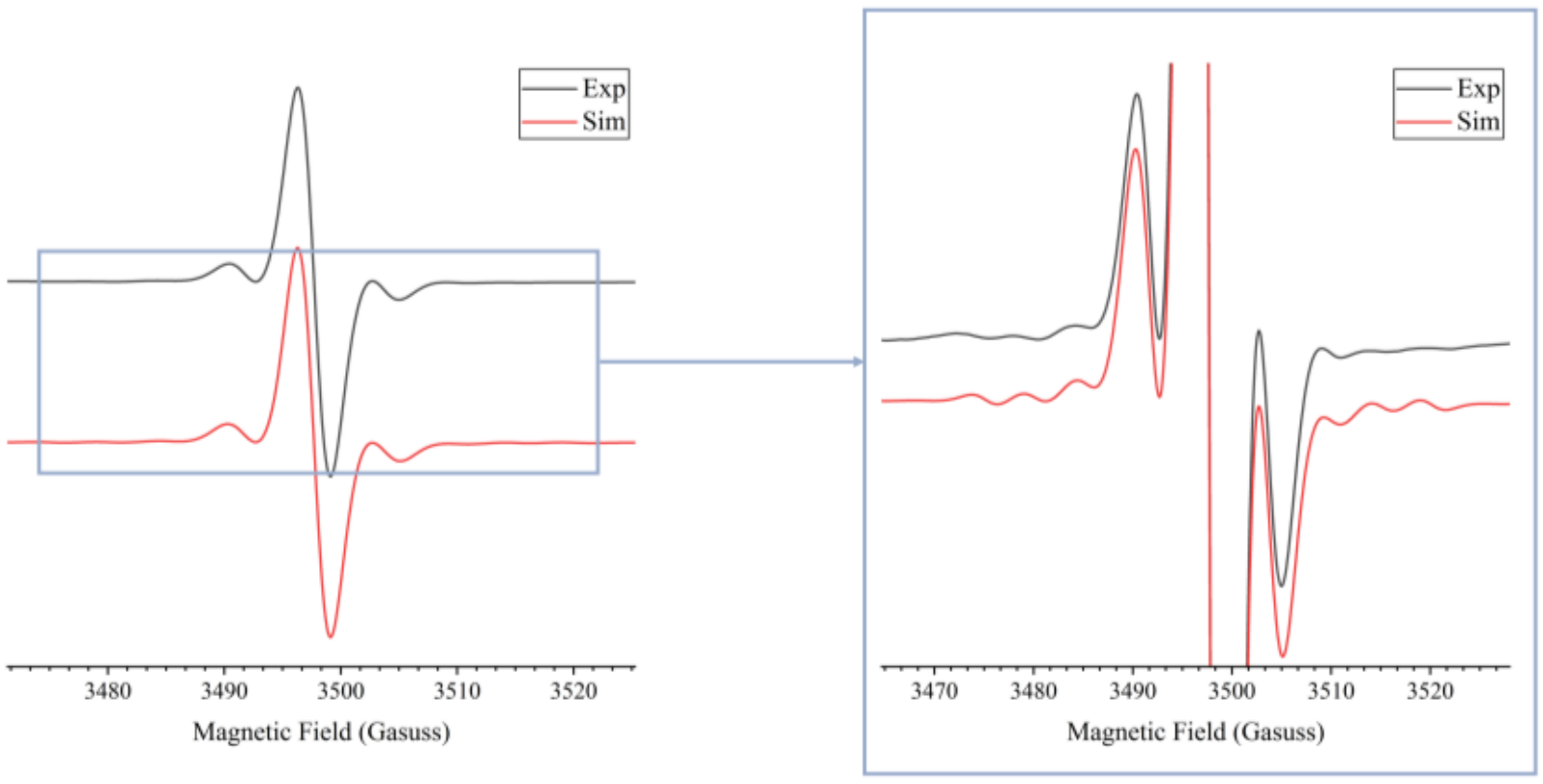

Fig. S10. The experimental (black) and simulated (red) EPR spectra of **3**·K(18-C-6) in toluene solution at 298 K.

6. Reactivity of compound 2

6.1 The reaction of compound 2 with tri-*n*-butyltin hydride

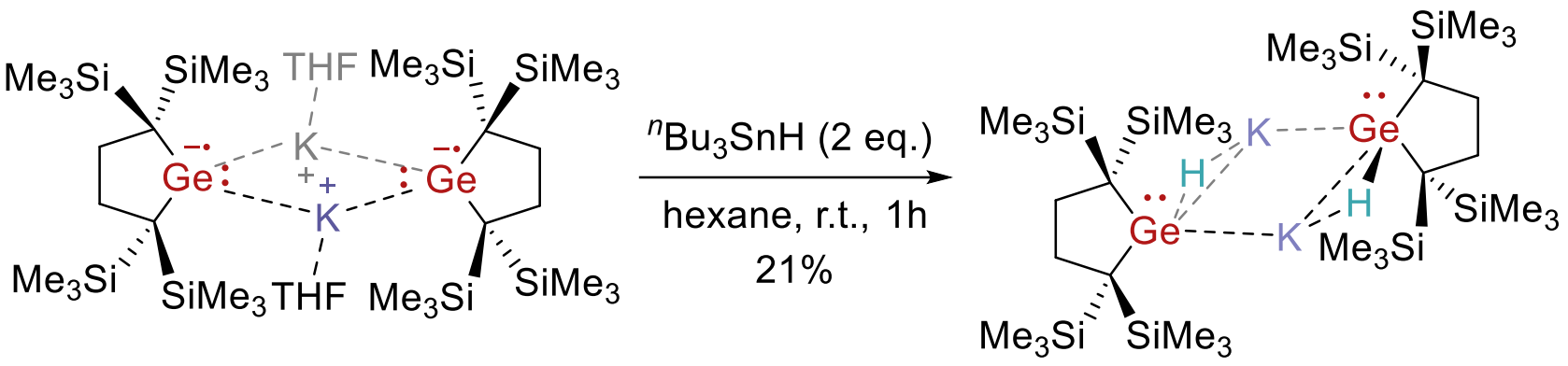

2 **4**

Tri-*n*-butyltin hydride (27.5 mg, 0.94 mmol) was added to an *n*-hexane (10 mL) solution of compound **2** (50 mg, 0.47 mmol) at room temperature. The mixture was stirred for 1 h, during which the solution color changed from deep red to pale yellow and a white precipitate formed. The precipitate was removed by filtration, and the filtrate was concentrated under vacuum. Recrystallization from *n*-pentane at -30 °C afforded the product as colorless crystals (9.1 mg, 21%).

$^{n}Bu_3Sn$-$Sn^{n}Bu_3$(δ = –83.0 ppm) was detected by ^{119}Sn NMR{^{1}H} NMR in the reaction crude reaction mixture.[2]

M.p.: 76.1 – 76.8 °C (dec.).

^{1}H NMR (400 MHz, THF-d_8): δ = 0.02 (s, 36H, Si*Me*$_3$), 0.04 (s, 36H, Si*Me*$_3$) , 1.87 (d, 8H, q*CH*$_2$-*CH*$_2$), 3.70 (s, 2H, Ge*H*).

^{13}C NMR (101 MHz, THF-d_8): δ = 10.8 (s, TMS-q*C*), 38.3 (s, backbone-q*C*), 3.7 (s, Si-*C*H$_3$) , 3.8 (s, Si-*C*H$_3$).

^{13}C Dept NMR (101 MHz, THF-d_8): δ = 38.3 (s, backbone-q*C*), 3.7 (s, Si-*C*H$_3$) , 3.8 (s, Si-*C*H$_3$).

^{29}Si NMR (99 MHz, THF-d_8): δ = –1.6 (s), 1.1 (s).

FT-IR: (solid, cm^{-1}): $\nu_{Ge\text{-}H}$ = 1736.

HRMS (ESI): *m/z* calculated for $C_{32}H_{82}Ge_2K_2Si_8$ [M+Na]$^+$ 937.2277, found 937.2254.

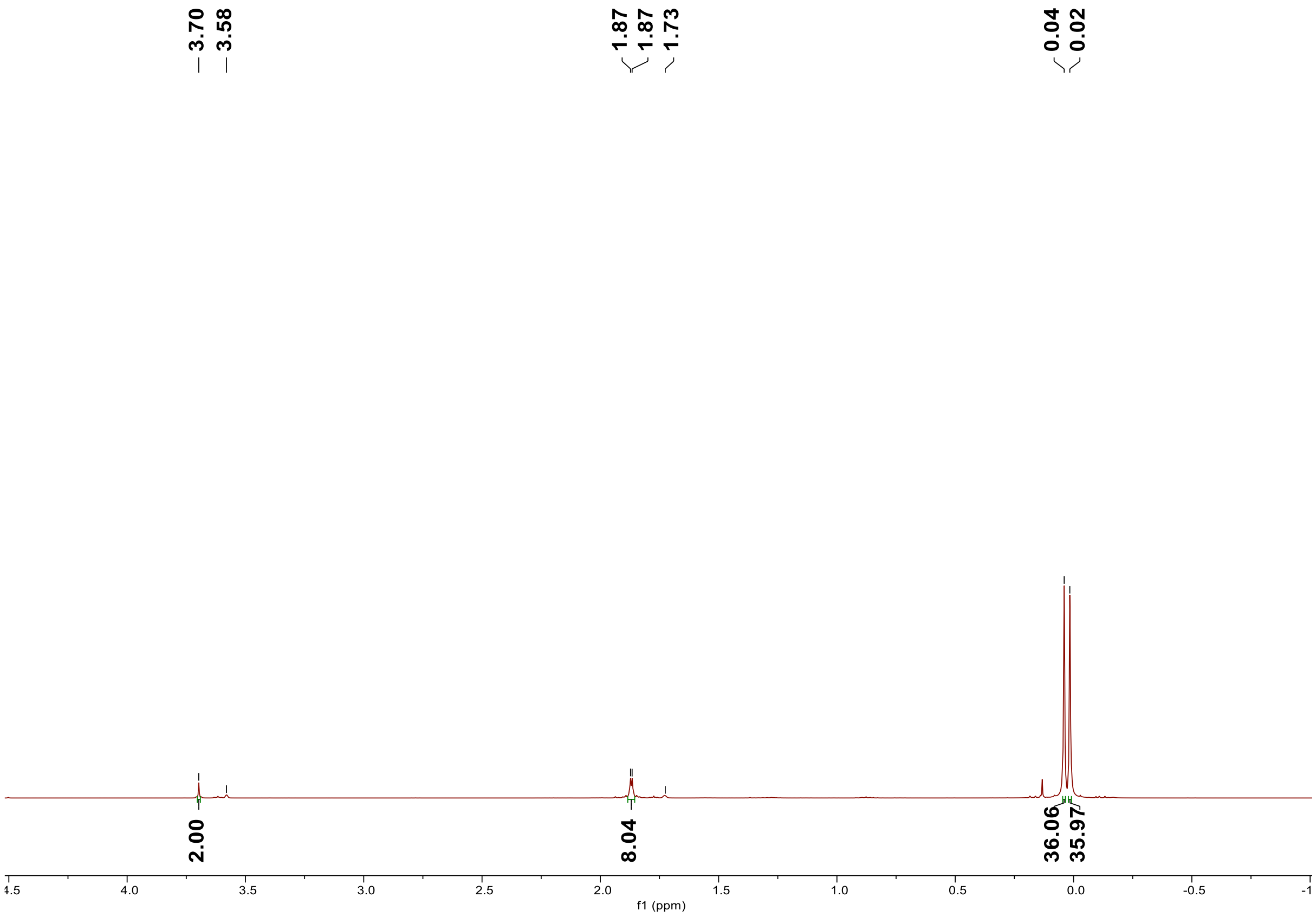

Fig. S11. ^{1}H NMR (400 MHz, THF-d_8) spectrum of compound **4**.

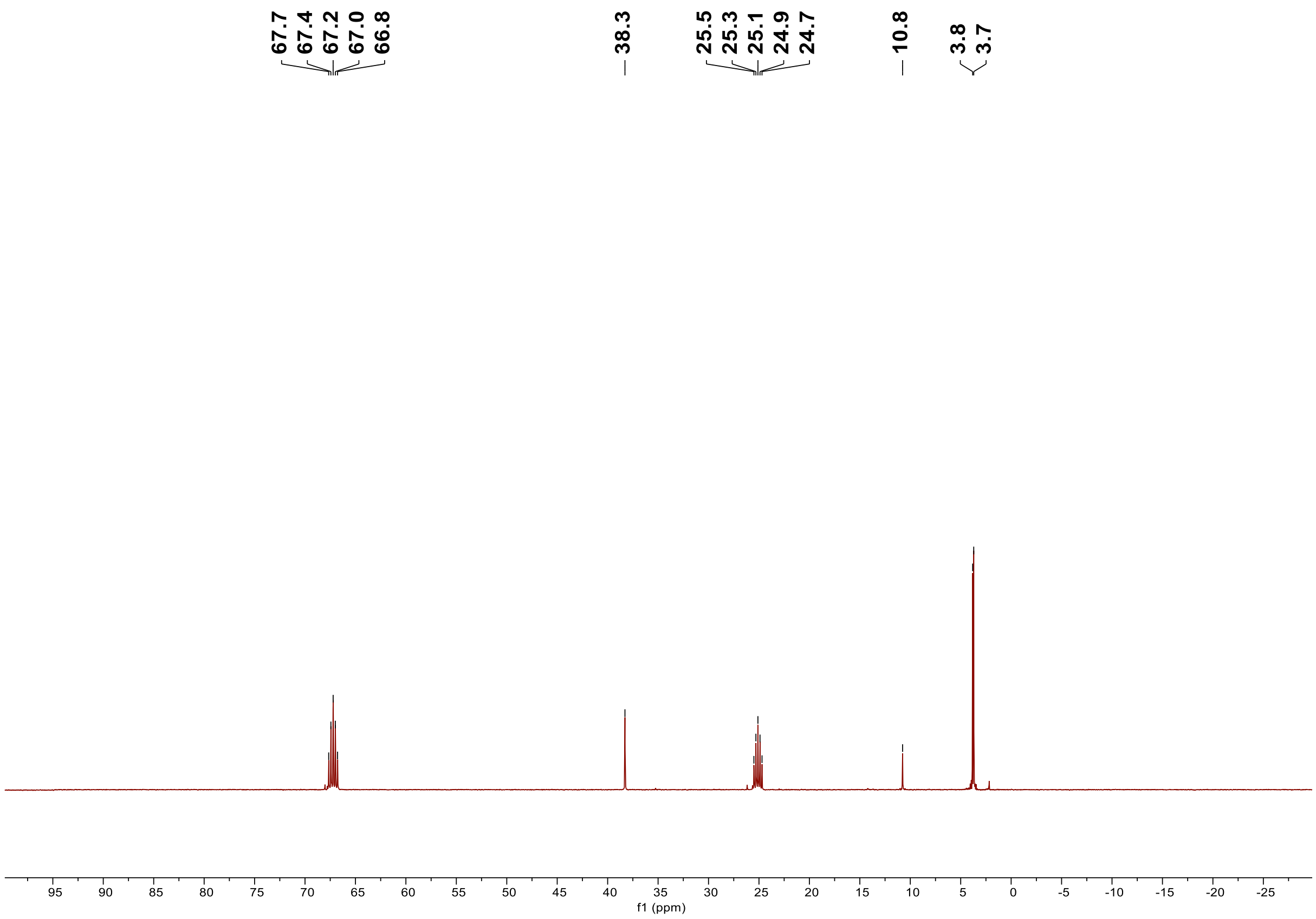

Fig. S12. ^{13}C NMR (101 MHz, THF-d_8) spectrum of **4**.

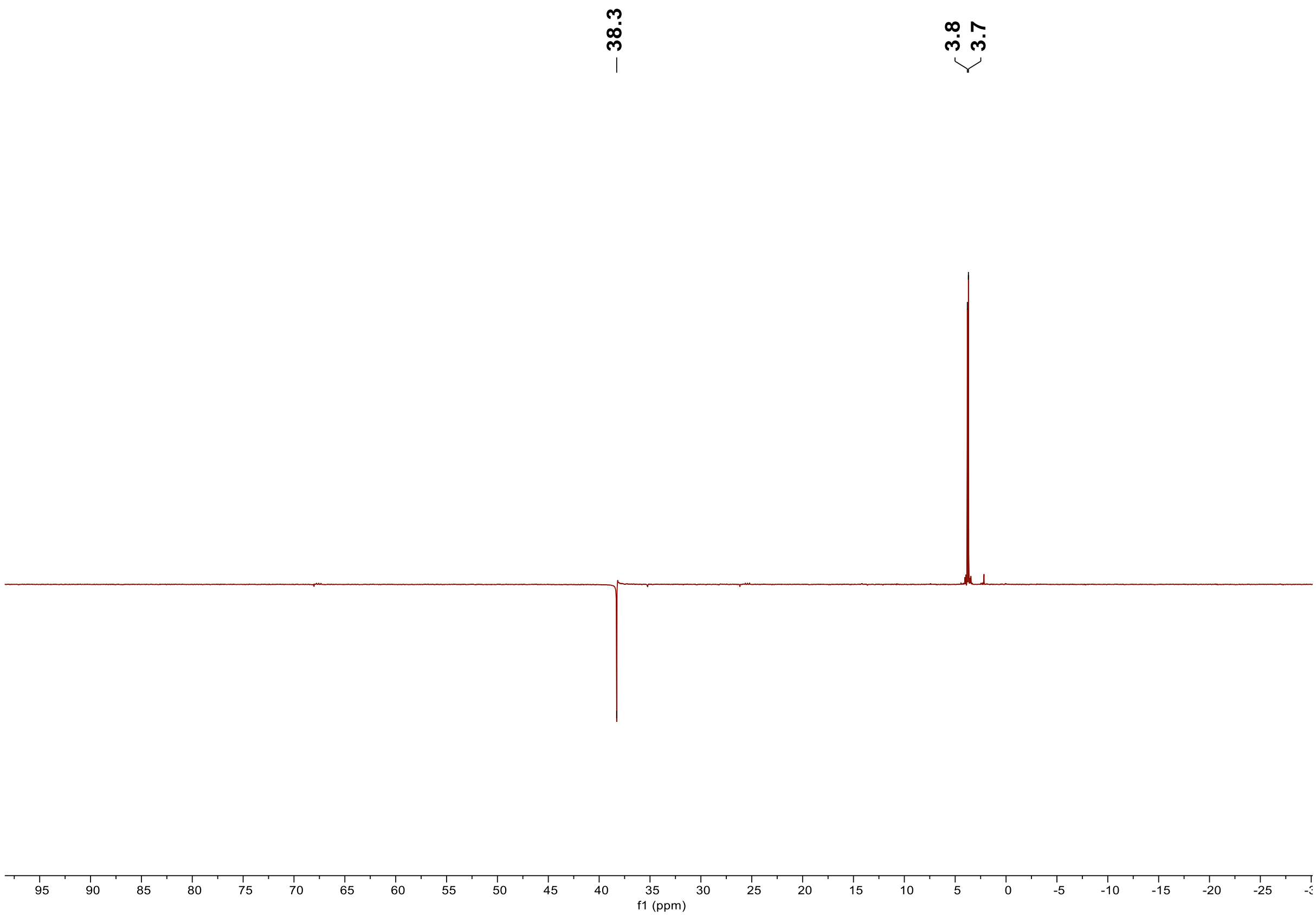

Fig. S13. ^{13}C Dept135 NMR (101 MHz, THF-d_8) spectrum of **4**.

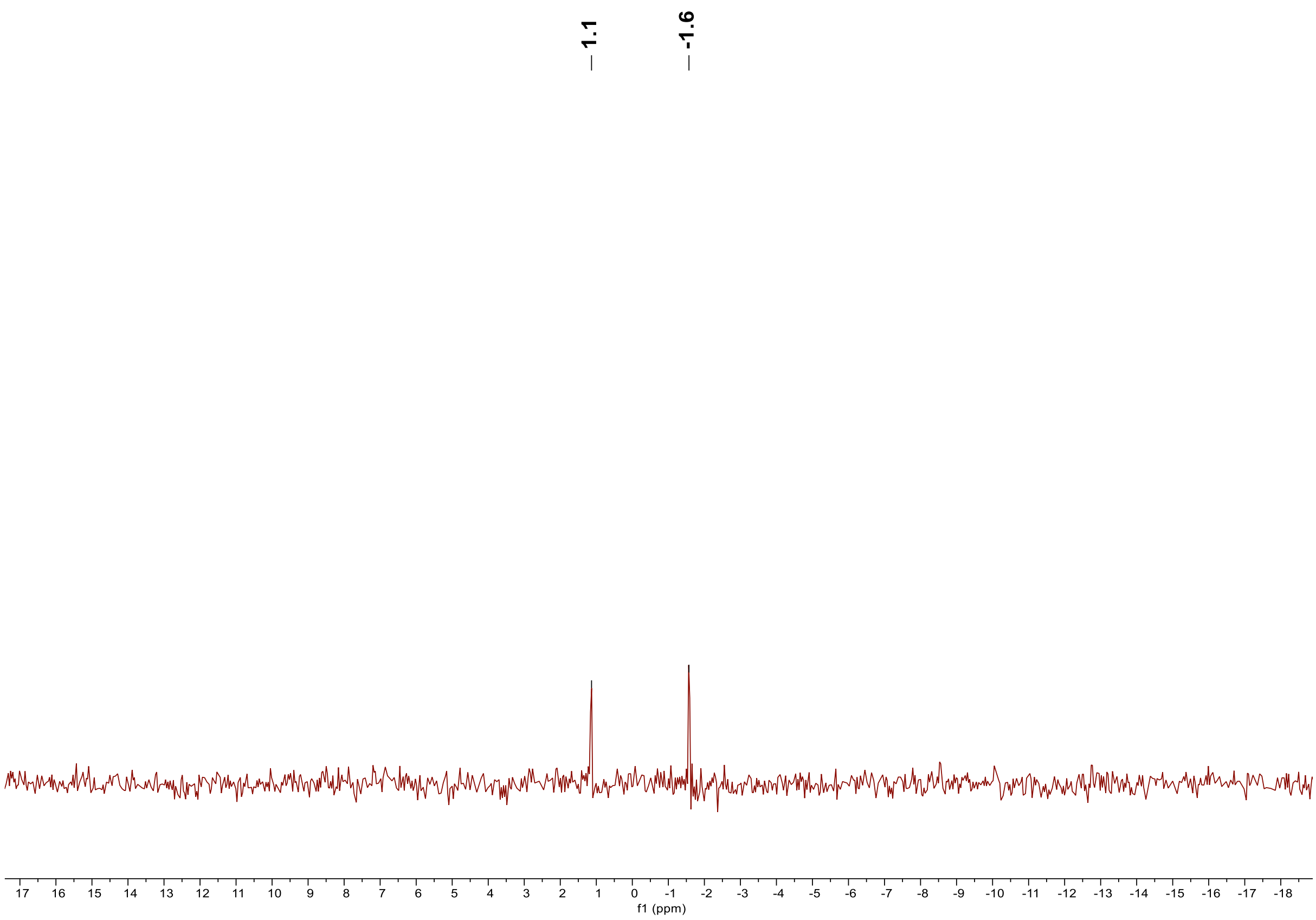

Fig. S14. ^{29}Si NMR (99 MHz, THF-d_8) spectrum of compound **4**.

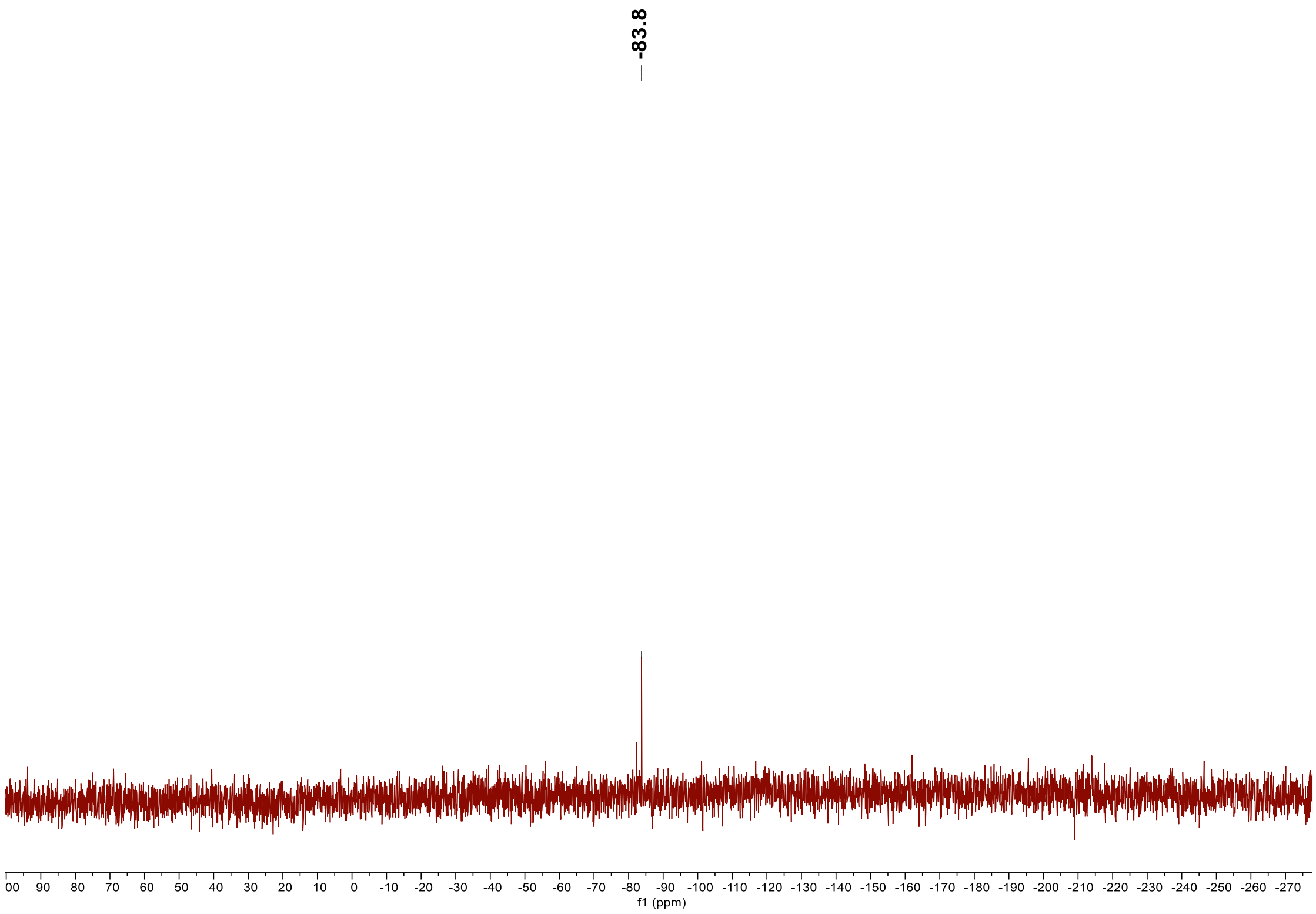

Fig. S15. ^{119}Sn NMR (99 MHz, benzene-d_6) spectrum of crude product.

6.2 The reaction of compound 2 with iodine

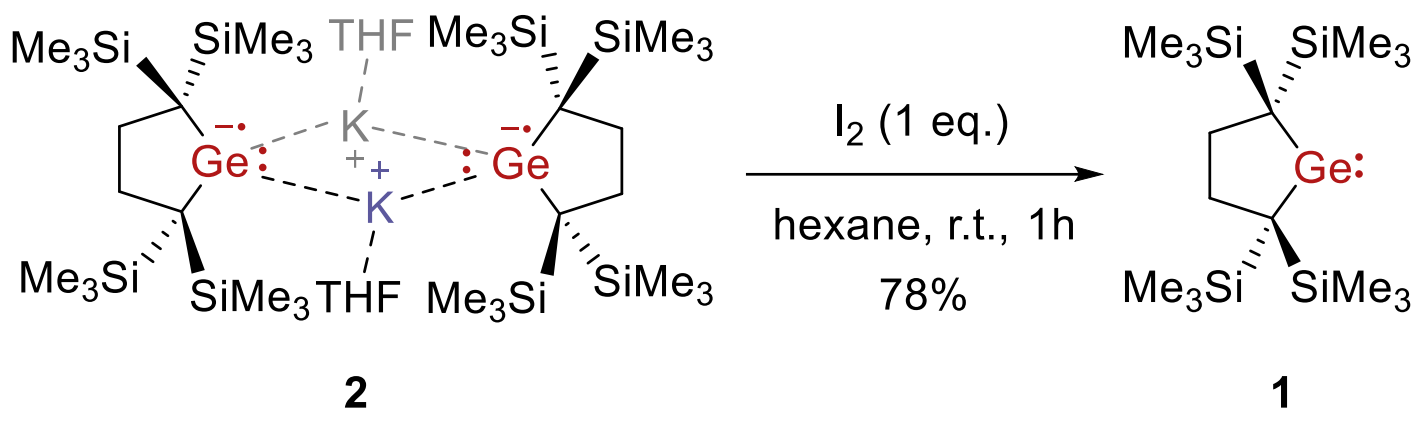

Compound **2** (50 mg, 0.47 mmol) and iodine (12 mg, 0.47 mmol) were added to an *n*-hexane (10 mL) solution at room temperature. The mixture was stirred for 1 h, during which the solution color changed from dark red to orange and a white precipitate formed. The precipitate was removed by filtration, affording an orange solution. Removal of the solvent under vacuum yielded the germylene **1** as a yellow solid (15.6 mg, 78%).

^{1}H NMR (400 MHz, benzene-d6): δ = 2.39 (s, 4H, CH_2-CH_2), 0.22 (s, 36H, SiMe_3).

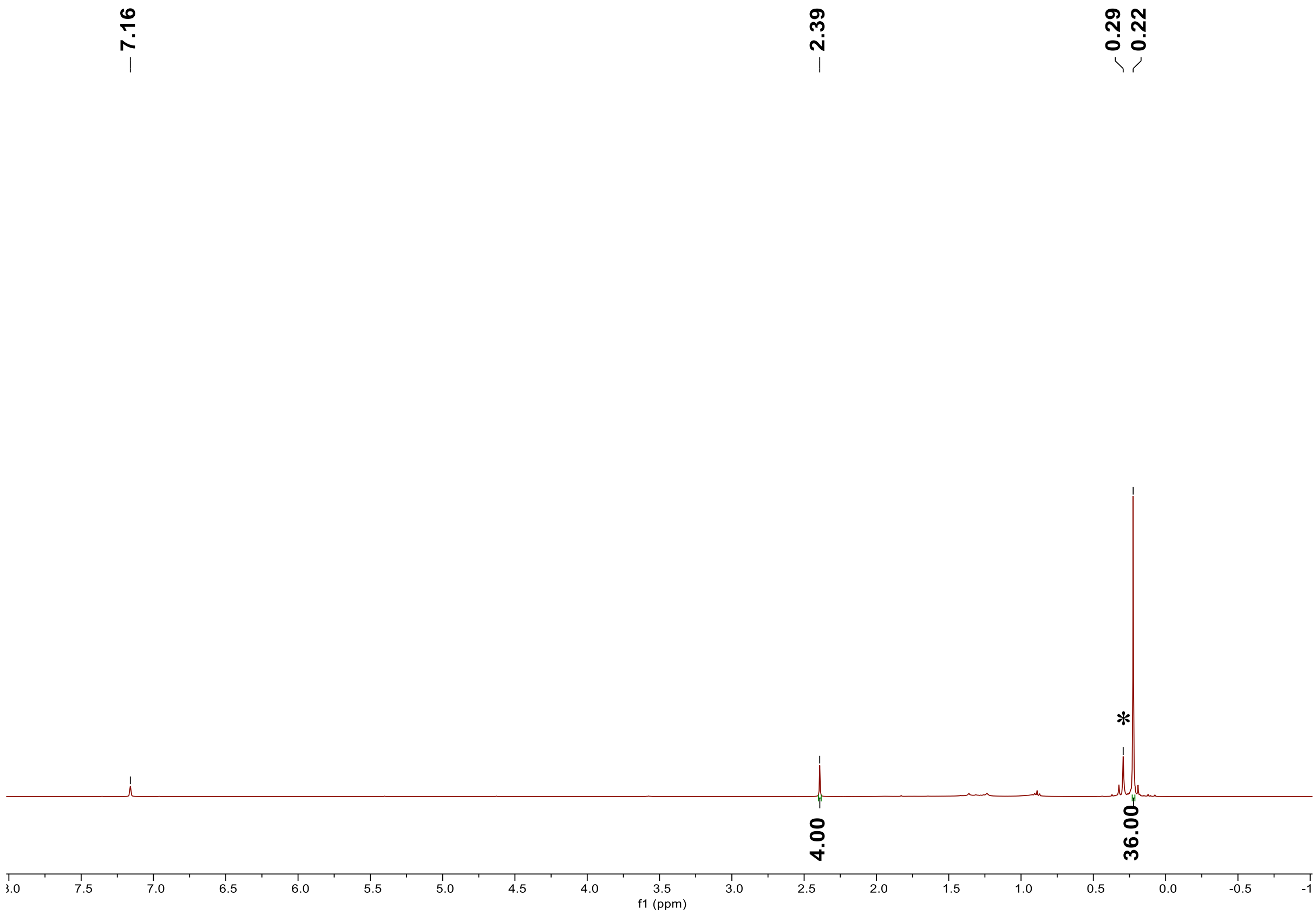

Fig. S16. ^{1}H NMR (400 MHz, benzene-d_6) spectrum of compound **1** (*=silicone grease).

6.3 The reaction of compound 2 with 2,3-Dimethyl-1,3-butadiene

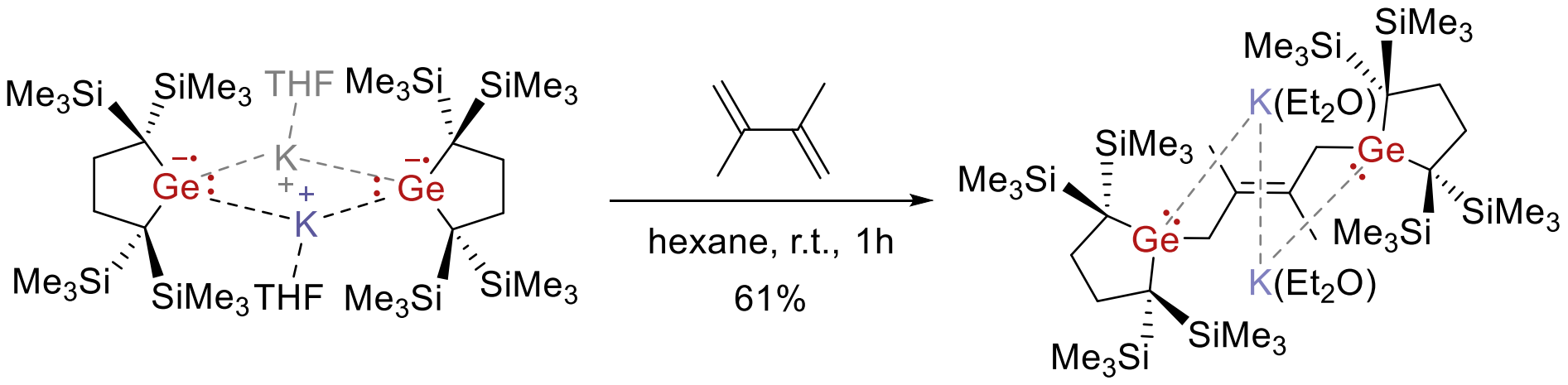

2,3-Dimethyl-1,3-butadiene (50 mg, 0.47 mmol) was added to a diethyl ether (5 mL) solution of compound **2** (50 mg, 0.47 mmol) at room temperature. The mixture was stirred for 1 h, during which the solution color changed from dark red to pale yellow. Removal of the diethyl ether under vacuum afforded the product as a yellow solid. Slow evaporation of the solution afforded compound **5** as colorless crystals (37.2 mg, 61%).

M.p.: 75.7 – 76.3 °C (dec.).

^{1}H NMR (400 MHz, THF-d_8): δ = 1.94 (s, 8H, qCH_2-CH_2), 0.09-0.10 (d, 72H, Si*Me*$_3$) , 1.09-1.13 (m, 12H, J = 0.2 Hz, OCH_2CH_3), 3.35-3.41 (m, 8H, J = 2.0 Hz, OCH_2CH_3), 1.74 (s, 6H, CCH_3), 2.13 (s, 4H, CCH_2).

^{13}C NMR (101 MHz, THF-d_8): δ = 4.5 (s, $Si(CH_3)_3$), 5.5 (s, $Si(CH_3)_3$), 15.3 (s, backbone-q*C*), 15.5 (s, C-CH_3), 22.9 (s, OCH_2CH_3), 66.1 (s, OCH_2CH_3), 29.0 (s, backbone-qCH_2), 37.1 (s, CCH_2), 128.7 (s, CH_3CCH_2).

^{13}C Dept NMR (101 MHz, THF-d_8): δ = 4.5 (s, $Si(CH_3)_3$), 5.5 (s, $Si(CH_3)_3$) , 15.5 (s, C-CH_3), 22.9 (s, OCH_2CH_3), 66.2 (s, OCH_2CH_3), 29.0 (s, backbone-qCH_2), 37.1 (s, CCH_2).

^{29}Si NMR (99 MHz, THF-d_8): δ = –0.3 (s), 1.8(s).

FT-IR: (solid, cm^{-1}): $\nu_{Ge\text{-}H}$ = 2240, 2087

HRMS (ESI): *m/z* calculated for $C_{54}H_{130}Ge_2K_2Si_8O_4$ $[M+H]^+$ 1292.6555, found 1292.6615.

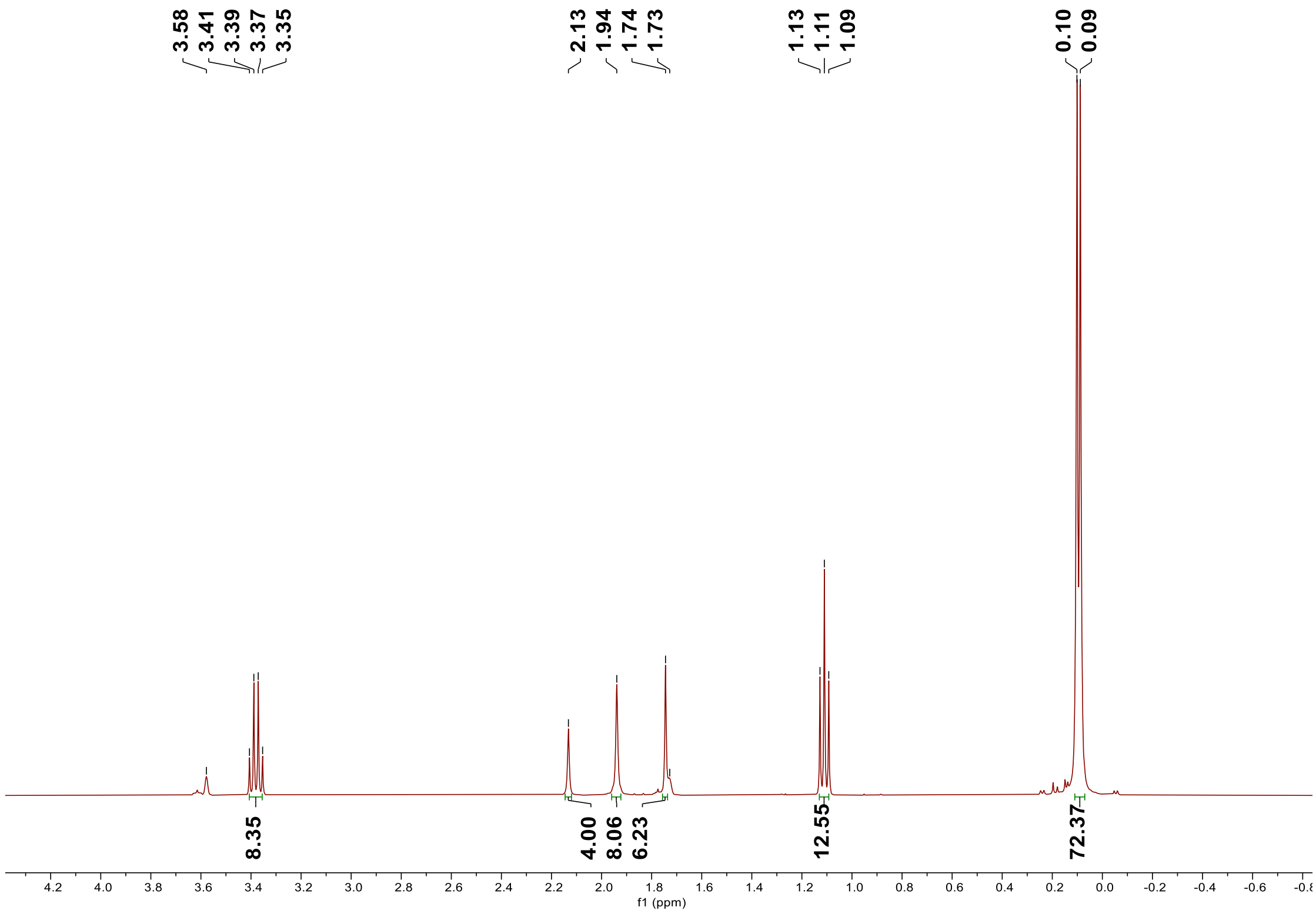

Fig. S17. ^{1}H NMR (400 MHz, THF-d_8) spectrum of compound **5**.

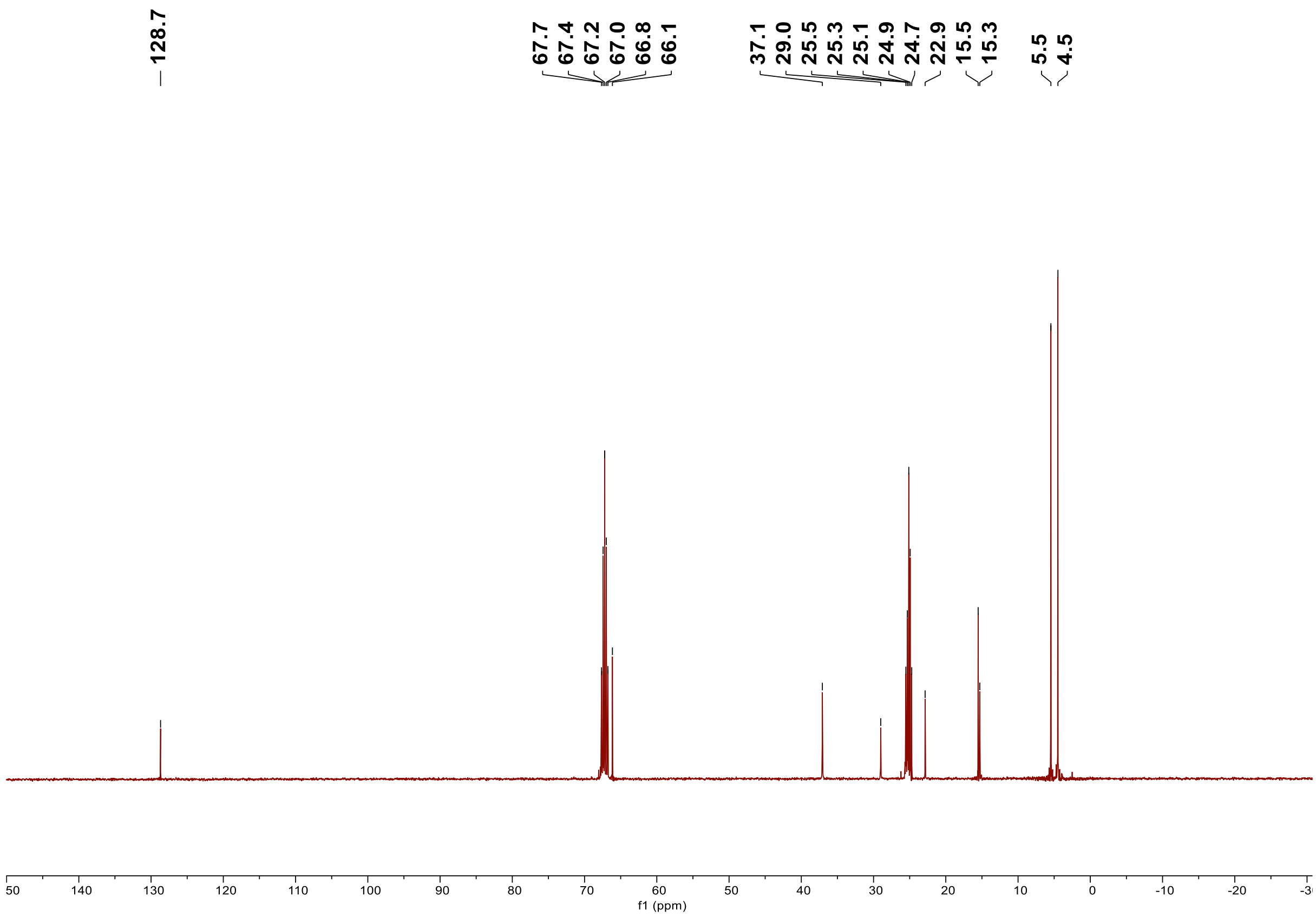

Fig. S18. ^{13}C NMR (101 MHz, THF-d_8) spectrum of **5**.

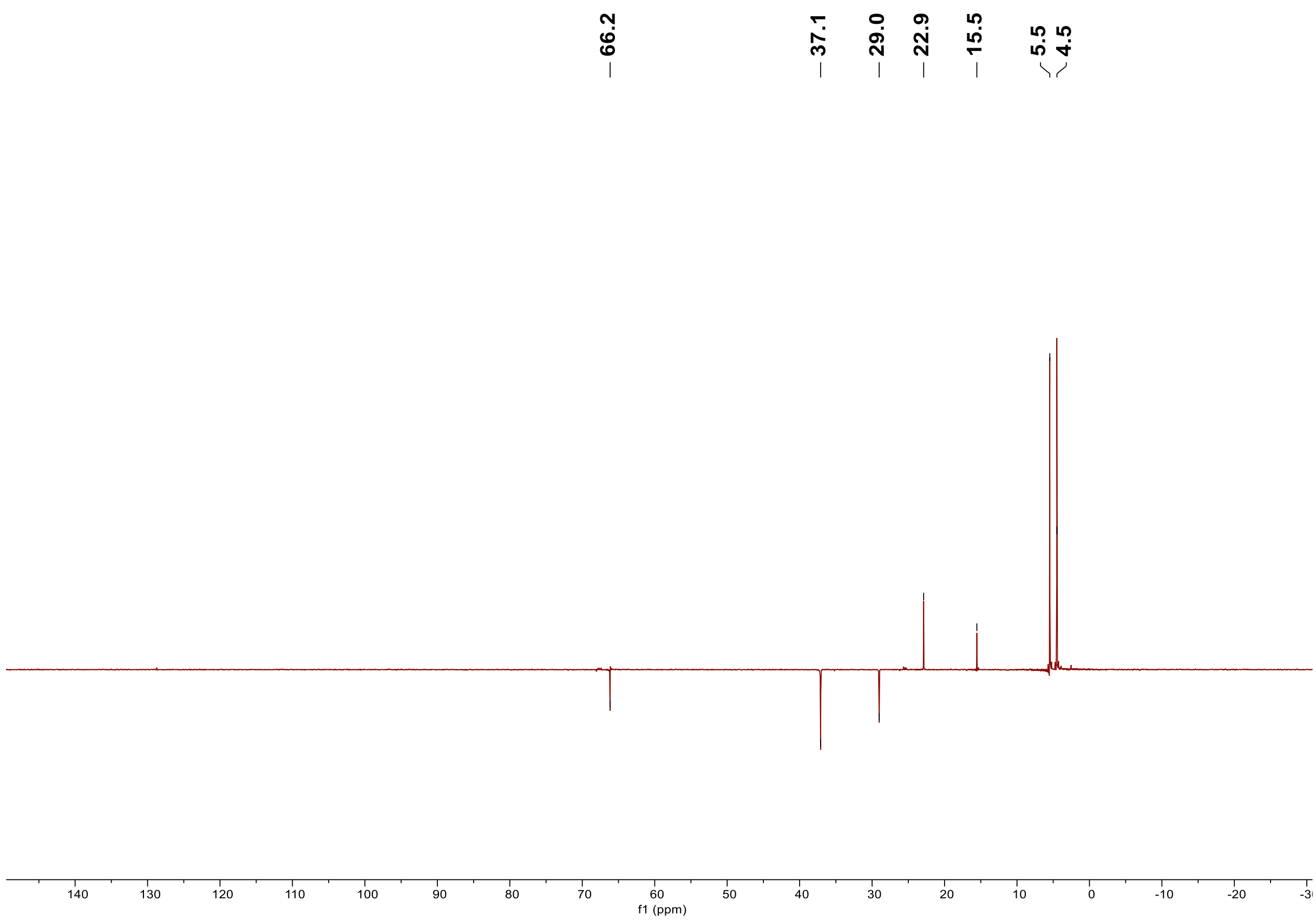

Fig. S19. ^{13}C Dept135 NMR (101 MHz, THF-d_8) spectrum of **5**.

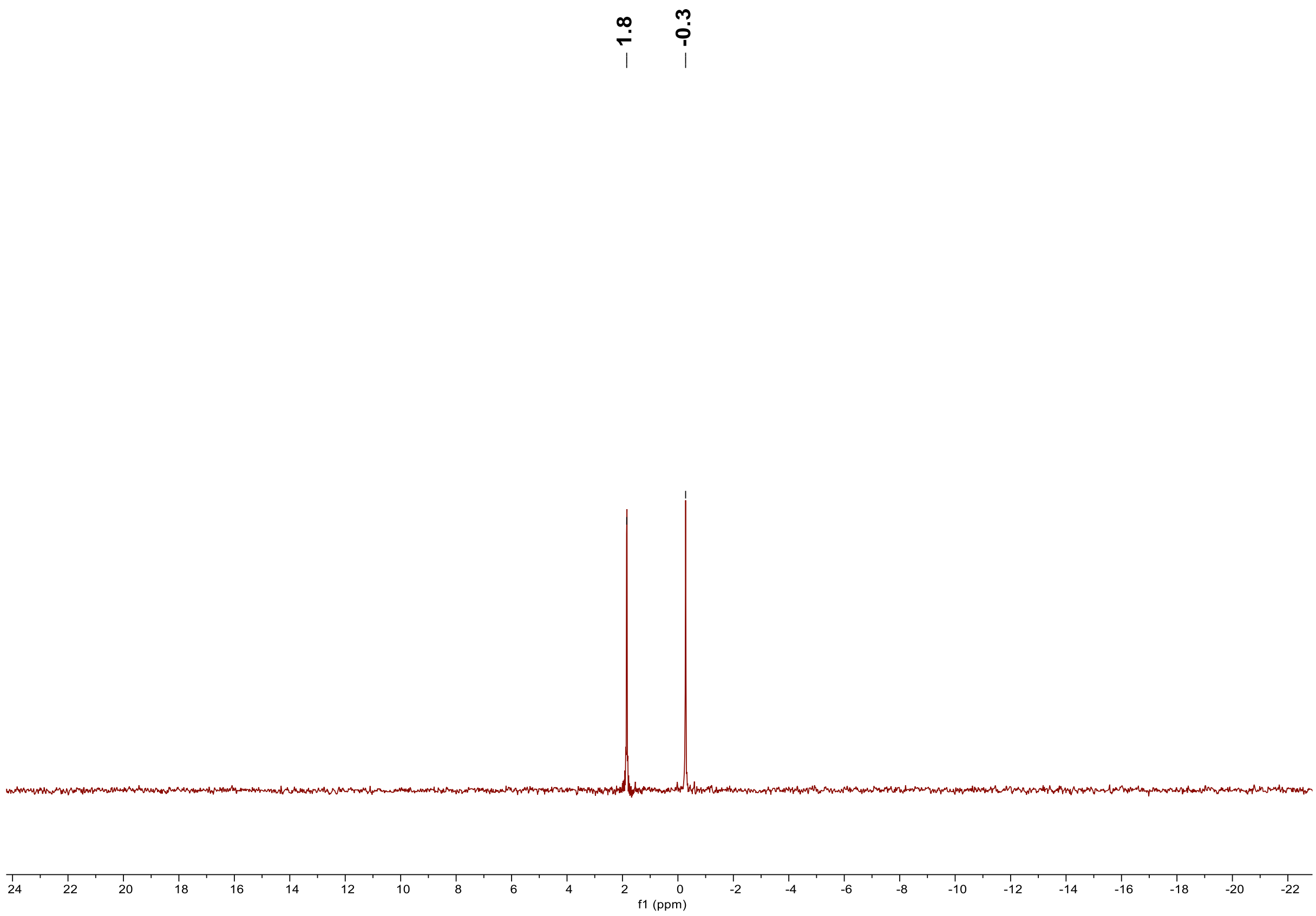

Fig. S20. ^{29}Si NMR (99 MHz, THF-d_8) spectrum of compound **5**.

6.4 The reaction of compound 2 with water

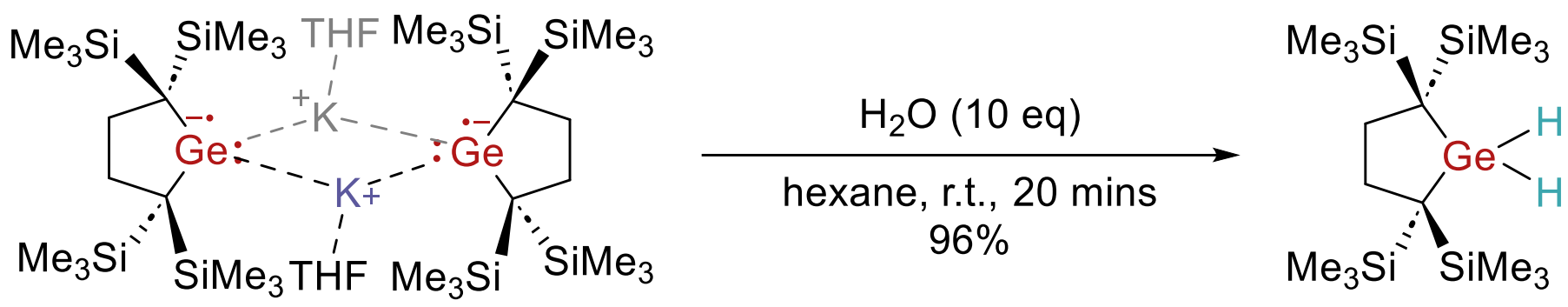

2 **6**

H_2O (169.3 µL, 9.4 mmol) was added to the *n*-hexane (5 mL) solution of compound **2** (100 mg, 0.94 mmol) at room temperature. The mixture was stirred for 1 h, during which the solution color changed from dark red to colorless. Removal of the *n*-hexane under vacuum afforded the product as a white solid. Slow evaporation of the solution afforded compound **6** as colorless crystals (38.4 mg, 96%).

M.p.: 75.9 – 76.5 °C (dec.).

^{1}H NMR (400 MHz, benzene-*d*$_6$): δ = 1.83 (s, 4H, CH_2-CH_2), 0.19 (s, 36H, SiMe_3) , 4.62 (s, 2H, GeH_2).

^{13}C NMR (101 MHz, benzene-*d*$_6$): δ = 2.4 (s, TMS-q*C*), 35.0 (s, backbone-q*C*), 7.8 (s, Si-CH_3).

^{13}C Dept NMR (101 MHz, benzene-*d*$_6$): δ = 35.0 (s, backbone-q*C*), 2.4 (s, Si-CH_3).

^{29}Si NMR (99 MHz, benzene-*d*$_6$): δ = 3.8 (s).

FT-IR: (solid, cm^{-1}): $\nu_{Ge\text{-}H}$ = 2043.

HRMS (ESI): *m/z* calculated for $C_{16}H_{42}GeSi_4$ $[M+H]^+$ 421.1648, found 421.1688.

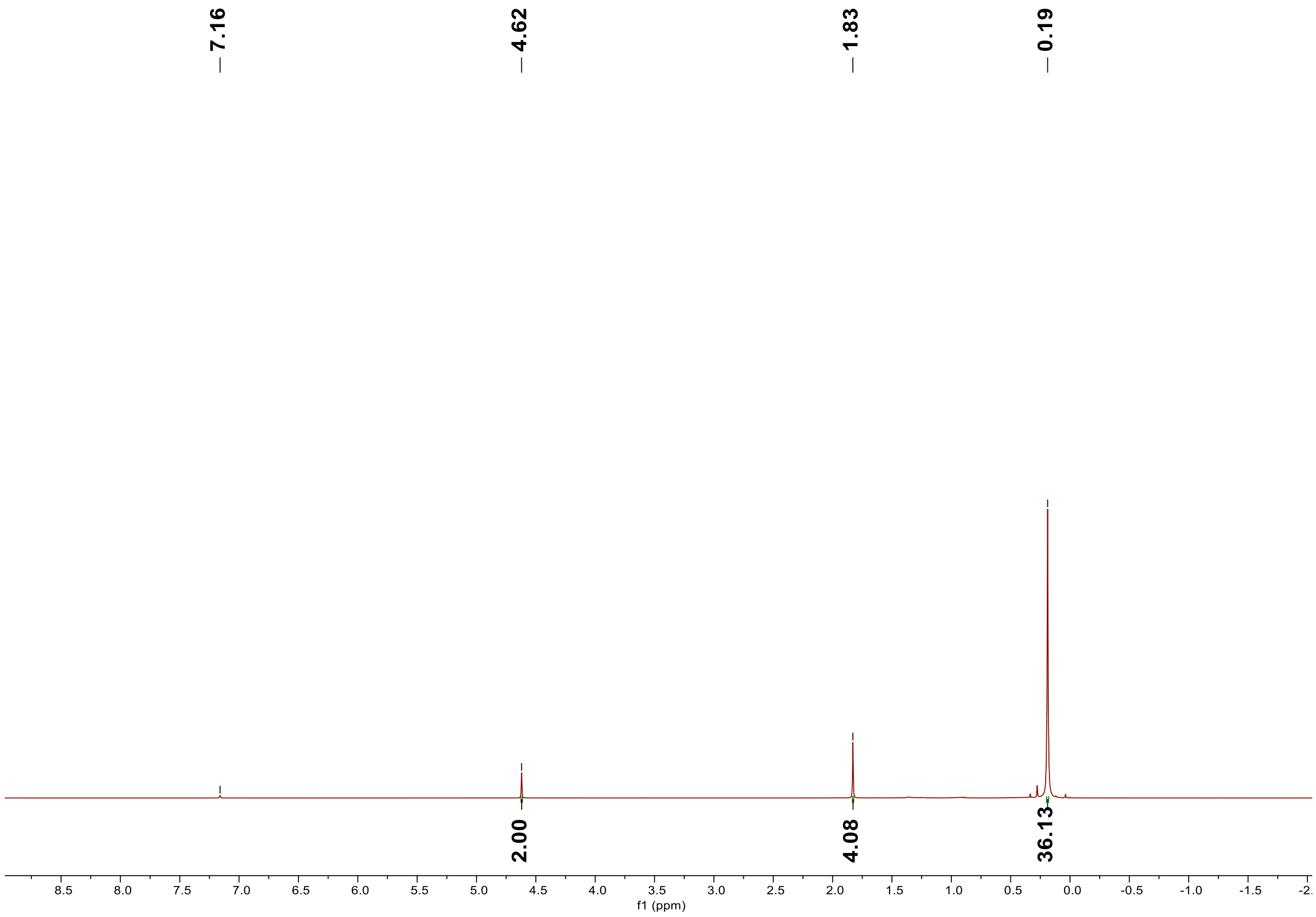

Fig. S21. ^{1}H NMR (400 MHz, benzene-d_6) spectrum of compound **6**.

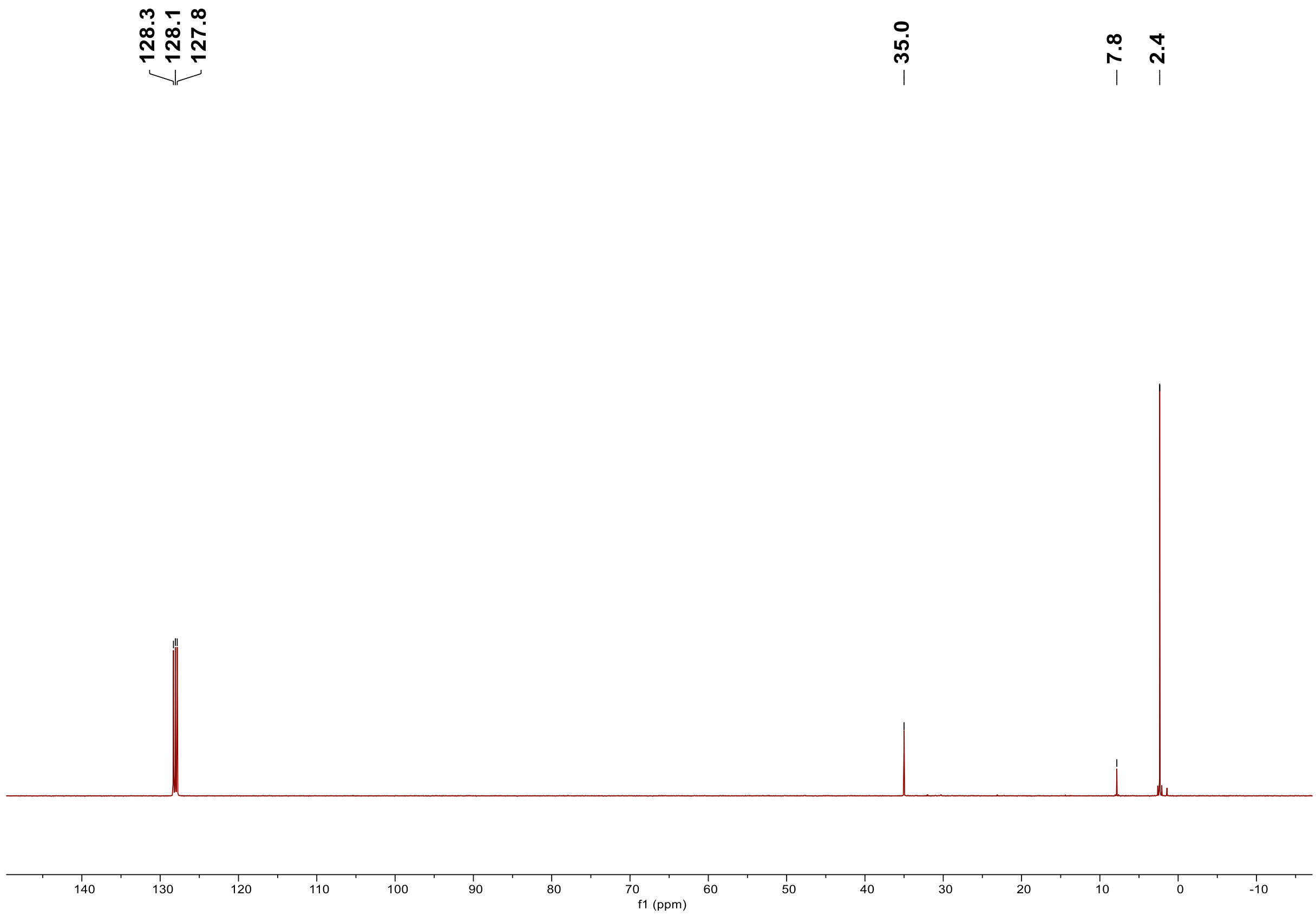

Fig. S22. ^{13}C NMR (101 MHz, benzene-d_6) spectrum of compound **6**.

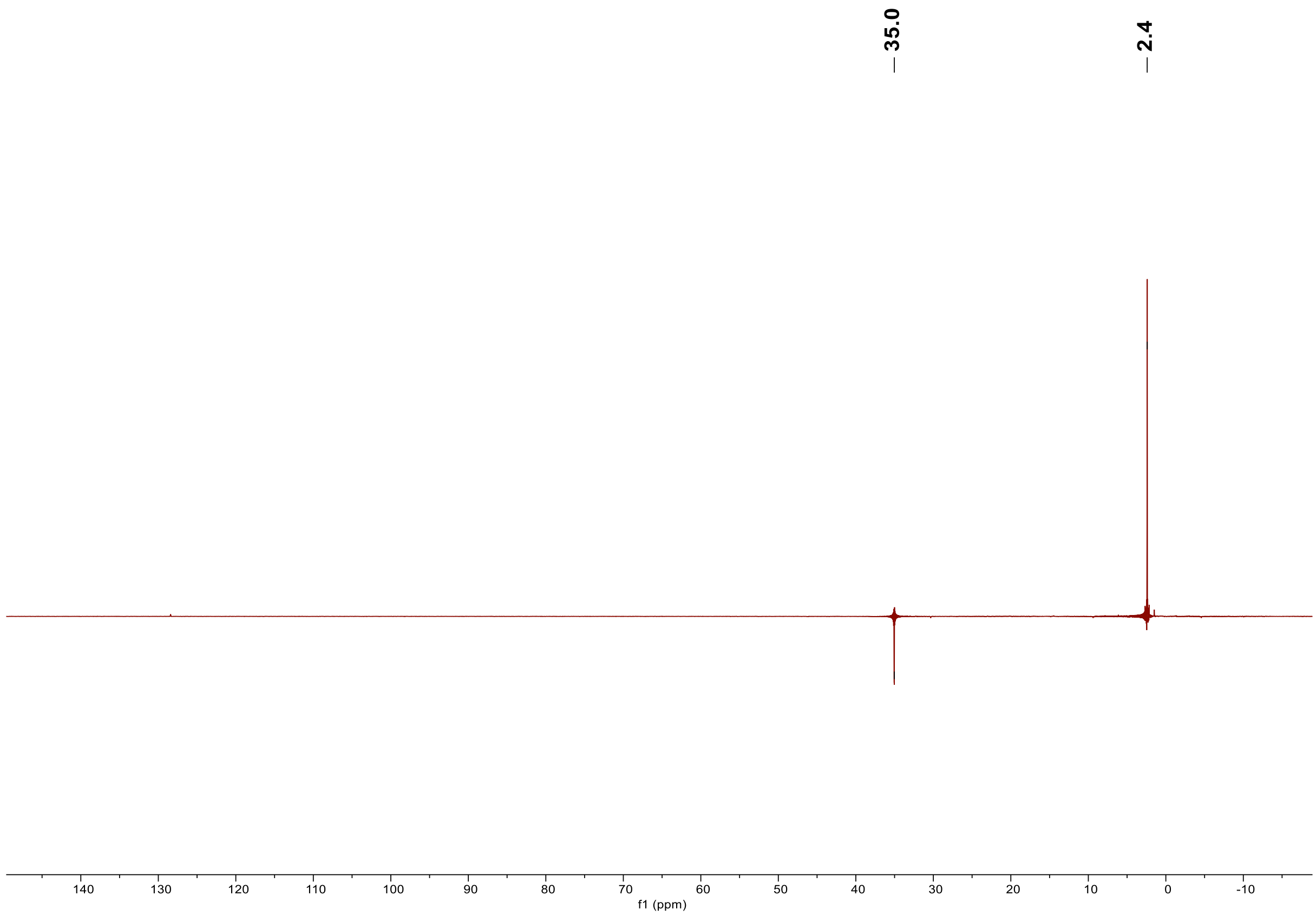

Fig. S23. ^{13}C Dept135 NMR (101 MHz, benzene-d_6) spectrum of compound **6**

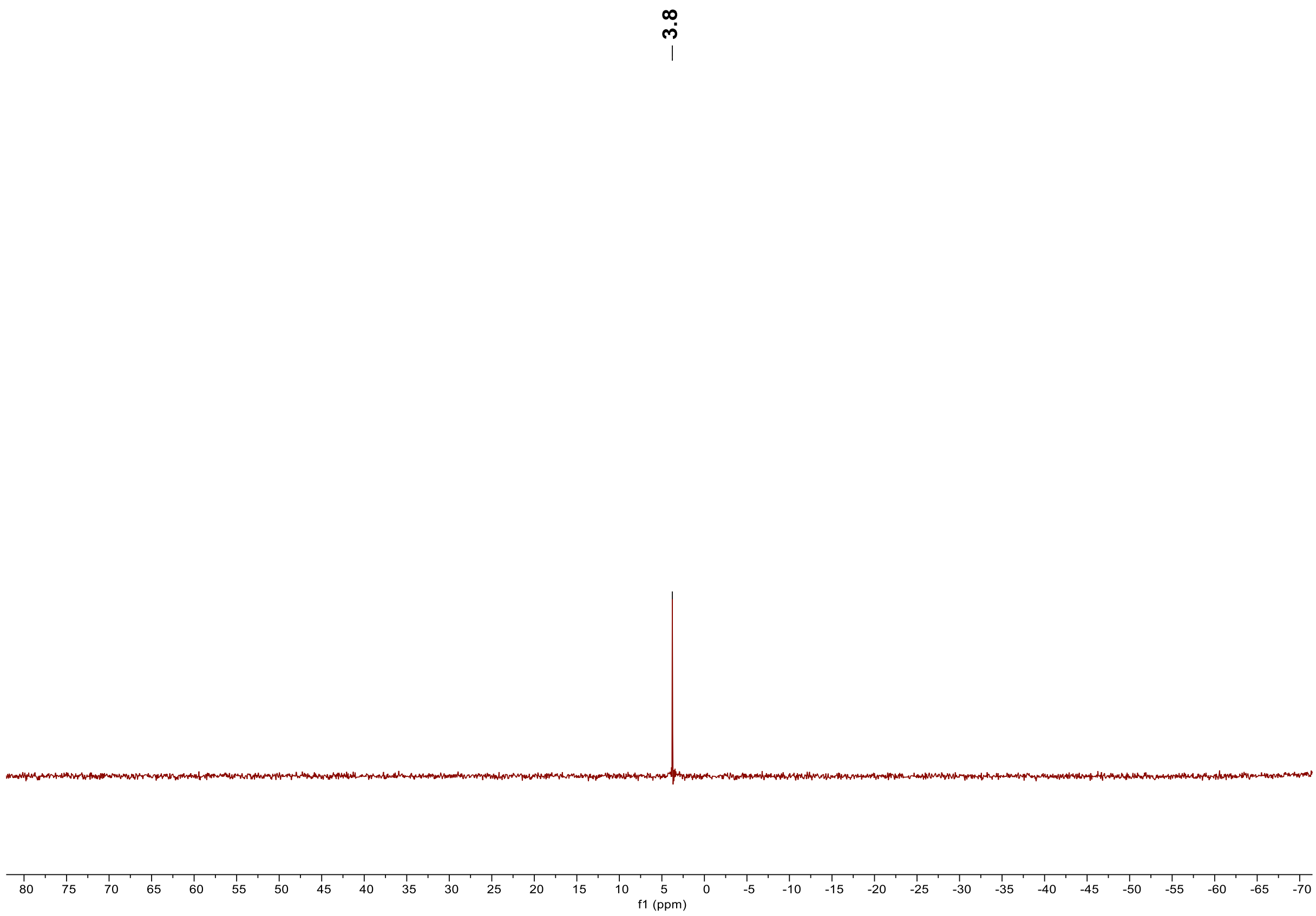

Fig. S24. ^{29}Si NMR (99 MHz, benzene-d_6) spectrum of compound **6**.

6.5 The reaction of compound 2 with Deuterated water

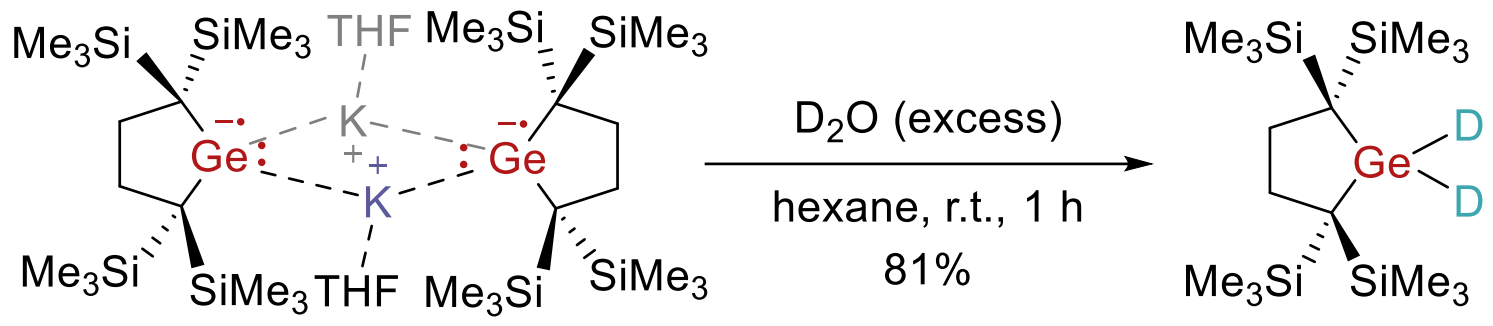

2 **8**

Deuterated water was added to the *n*-hexane (5 mL) solution of compound **2** (100 mg, 0.94 mmol) at room temperature. The mixture was stirred for 1 h, during which the solution color changed from dark red to colorless. Removal of the *n*-hexane under vacuum afforded the product as a white solid (32 mg, 81%).

^{1}H NMR (400 MHz, benzene-d_6): δ = 1.82 (s, 4H, CH_2-CH_2), 0.19 (s, 36H, SiMe_3) .

HRMS (ESI): m/z calculated for $C_{16}H_{40}D_2GeSi_4$ [M+H]$^+$ 423.1773, found 423.1742.

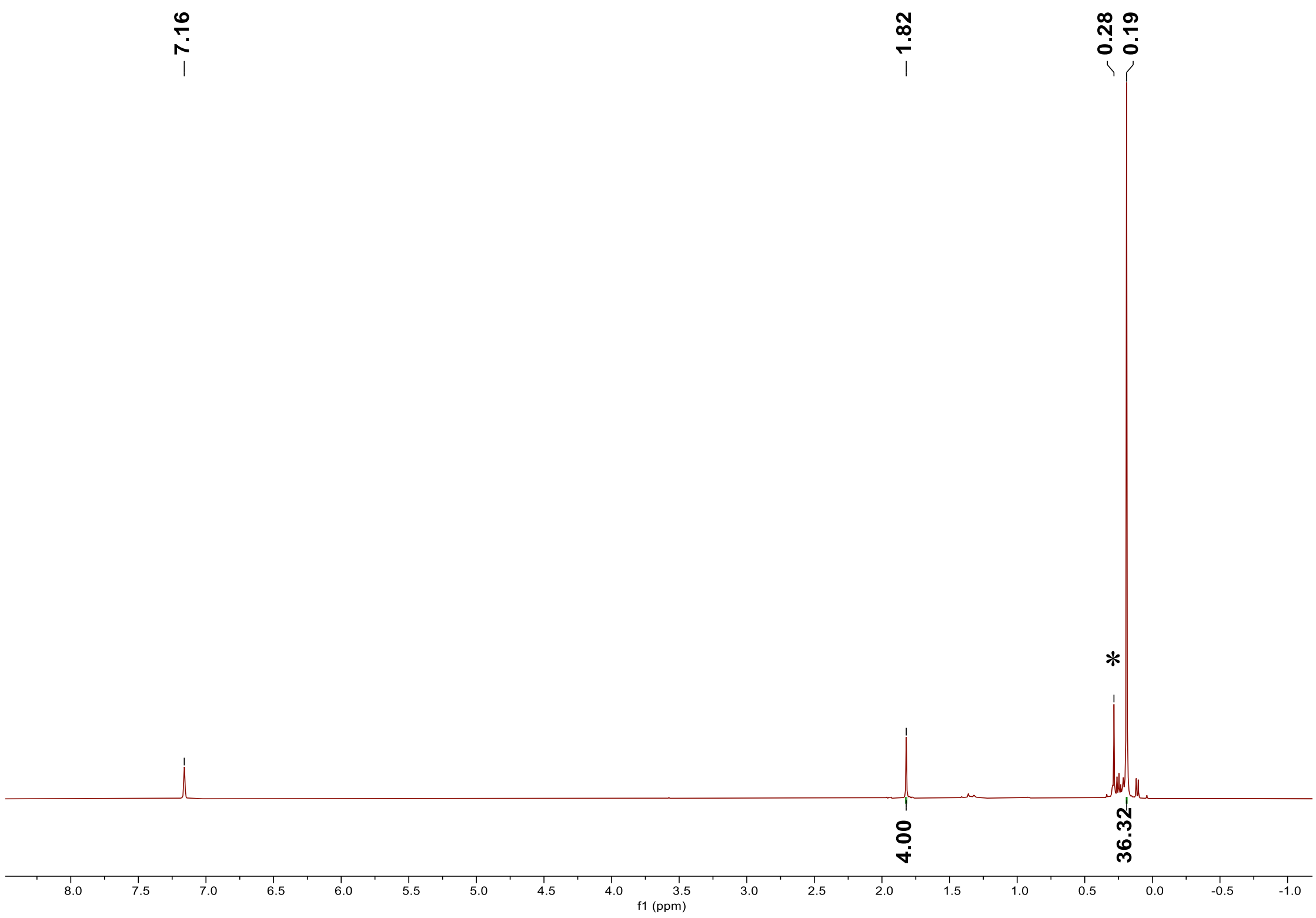

Fig. S25. ^{1}H NMR (400 MHz, benzene-d_6) spectrum of compound **8**. (*=silicone grease)

7. Fourier-transform infrared spectroscopy

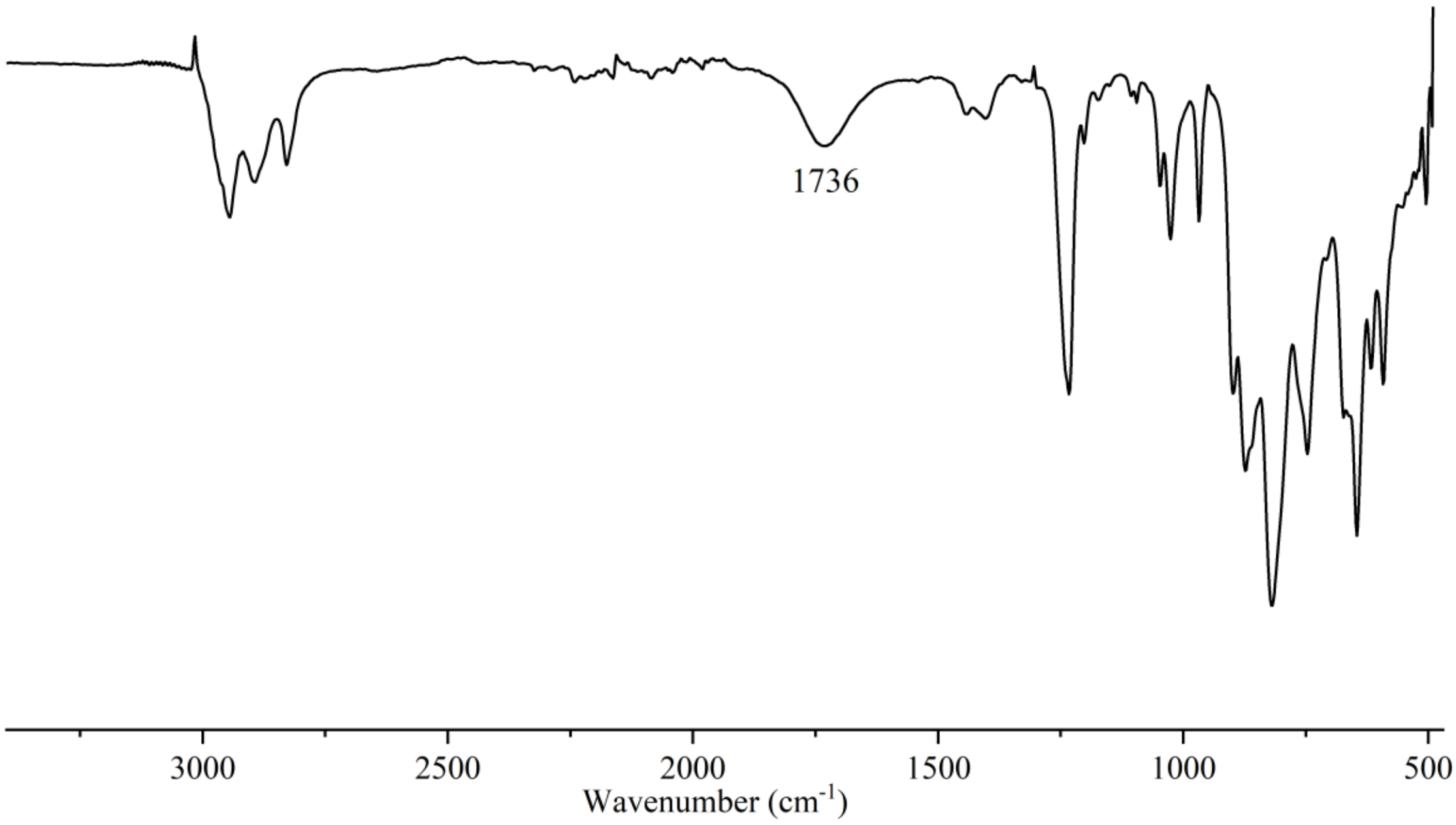

Fig. S26. FT-IR spectrum of compound **4**.

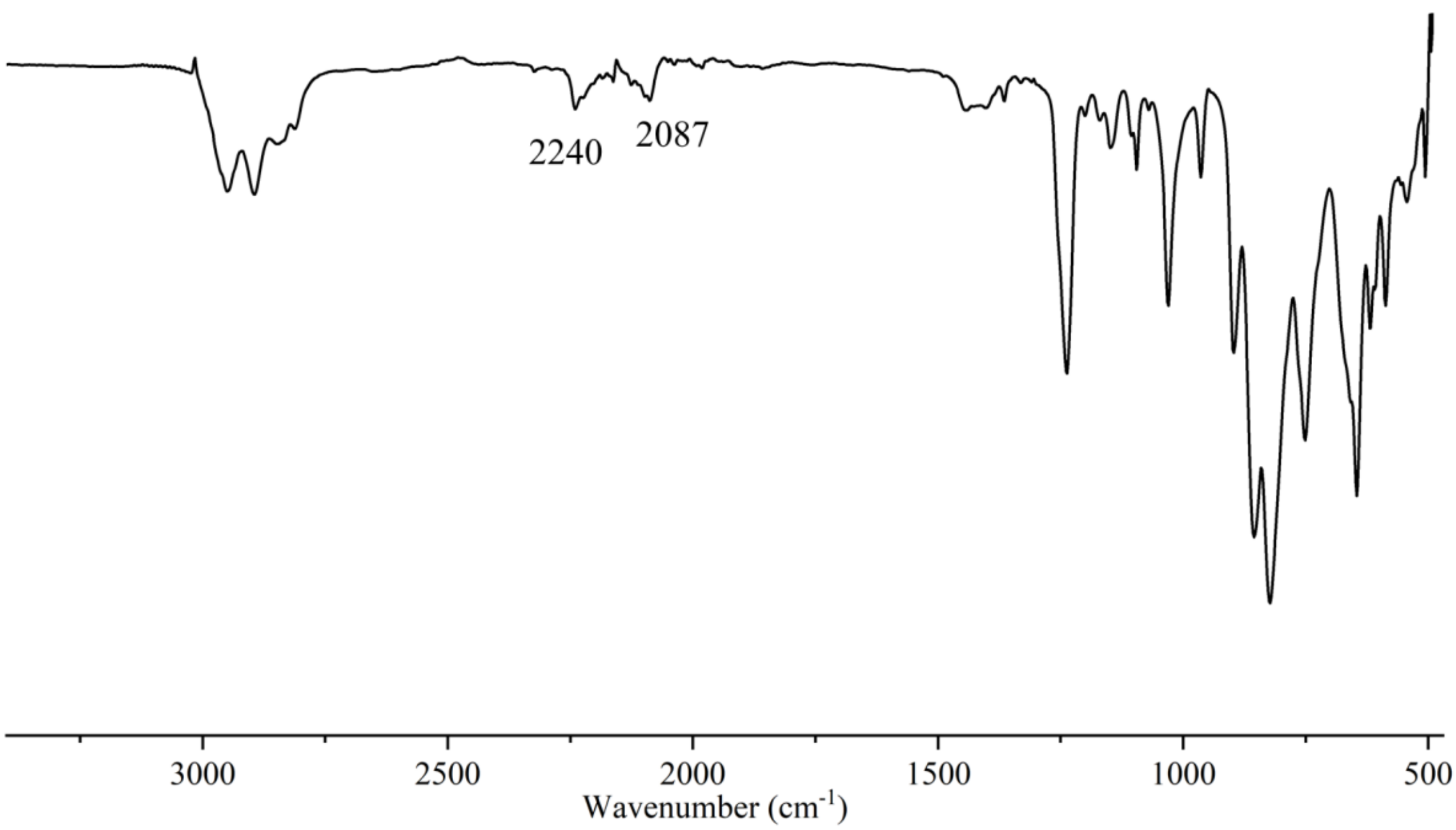

Fig. S27. FT-IR spectrum of compound **5**.

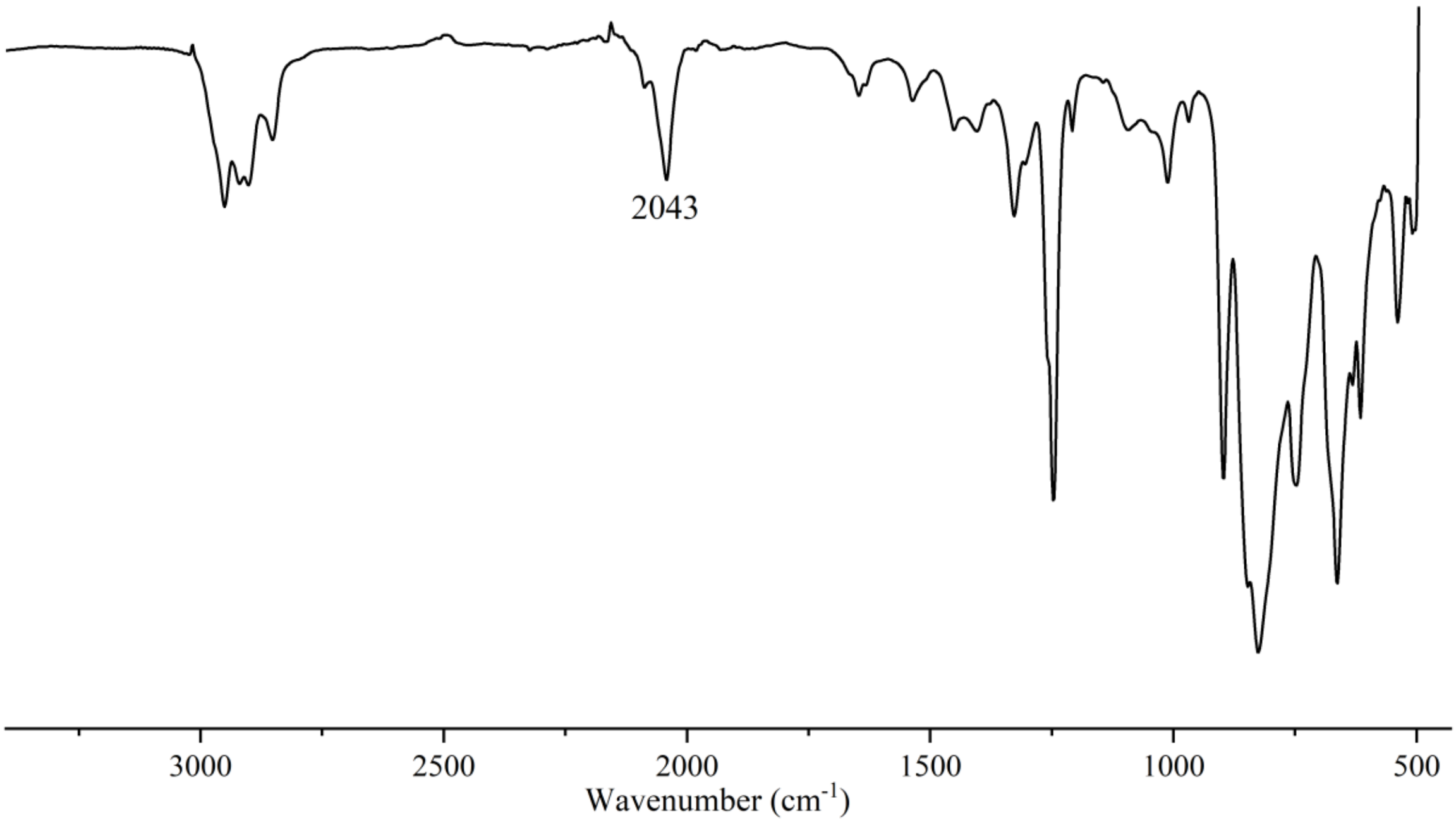

Fig. S28. FT-IR spectrum of compound **6**.

8. X-ray Crystallographic Studies

X-ray data collection and structural refinement. Intensity data for compounds **2, 3**·K(18-C-6)**, 4, 5, 6** were collected using a Bruker APEX II diffractometer. The structure was solved by direct phase determination (SHELX-2013) and refined for all data by full-matrix least squares methods on F^2. All non-hydrogen atoms were subjected to anisotropic refinement. The hydrogen atoms were generated geometrically and allowed to ride in their respective parent atoms; they were assigned appropriate isotropic thermal parameters and included in the structure-factor calculations. CCDC; 2513035-2513039 contains the supplementary crystallographic data for this paper. The data can be obtained free of charge from the Cambridge Crystallography Data Center via www.ccdc.cam.ac.uk/data_request/cif.

Table S1. Crystal data and structure refinement for compound **2** and **3**·K(18-C-6).

**Compounds**	**2**	**3**·K(18-C-6)
Formula	$C_{40}H_{96}Ge_2K_2O_2Si_8$	$C_{28}H_{64}GeKO_6Si_4$
Fw	1057.34	720.882
Crystal syst	Triclinic	Triclinic
Space group	P-1	P-1
T/K	173(2)	173(2)
a, Å	10.8448(6)	11.0048(4)
b, Å	11.3293(6)	12.0426(6)
c, Å	13.4356(8)	33.1989(17)
α, deg	105.810(2)	82.180(2)
β, deg	105.661(2)	87.994(2)
γ, deg	100.719(2)	66.680(2)
V, A^3	1468.10(14)	4001.9(3)
Z	6	12
d_{calcd} Mg/cm^3	1.196	1.195
μ, mm^{-1}	1.357	1.022
Refl collected	29116	84484
T_{max}/ T_{min}	0.6794 / 0.5329	0.862 / 0.750
F(000)	566	1545
Independent	5185	16327
R (int)	0.1331	0.0917
R [I>2σ(I)]	0.0624	0.0591
wR2[I>2σ(I)]	0.1648	0.1498
GOF	1.248	1.073
Largest diff. peak/ hole[e. $Å^{-3}$]	0.602 / -1.391	0.701 / -0.799
CCDC	2513038	2513039

Table S2. Crystal data and structure refinement for compound **4**, **5** and **6**.

**Compounds**	**4**	**5**	**6**
Formula	$C_{32}H_{82}Ge_2K_2Si_8$	$C_{54}H_{130}Ge_2K_2O_4Si_8$	$C_{16}H_{42}GeSi_4$
Fw	915.07	1291.67	419.44
Crystal syst	Triclinic	Triclinic	Monoclinic
Space group	P-1	P-1	P 1 21/c 1
T/K	173(2)	173(2)	170(2)
a, Å	12.0180(3)	11.7712(3)	16.7818(12)
b, Å	14.4718(4)	13.0465(4)	6.9005(5)
c, Å	16.6526(4)	14.8559(4)	21.8834(14)
α, deg	113.7670(10)	98.0770(10)	90
β, deg	104.8720(10)	107.6480	108.776
γ, deg	90.0800(10)	112.0490	90
V, A^3	1539.53(11)	1929.75(9)	2399.3(3)
Z	2	1	4
d_{calcd} Mg/cm^3	1.195	1.111	1.161
μ, mm^{-1}	1.554	1.045	2.425
Refl collected	50147	57676	22098
T_{max}/ T_{min}	0.7466 / 0.5689	0.7467 / 0.6682	0.7519 /0.5689
F(000)	976	700	904
Independent	10789	13774	5466
R (int)	0.0594	0.0504	0.0610
R [I>2σ(I)]	0.0391	0.0377	0.0501
wR2[I>2σ(I)]	0.0969	0.0829	0.1261
GOF	1.121	1.024	1.128
Largest diff. peak/ hole[e. Å^{-3}]	0.441 / -0.550	0.652 / -0.296	2.787 / -0.962
CCDC	2513037	2513036	2513035

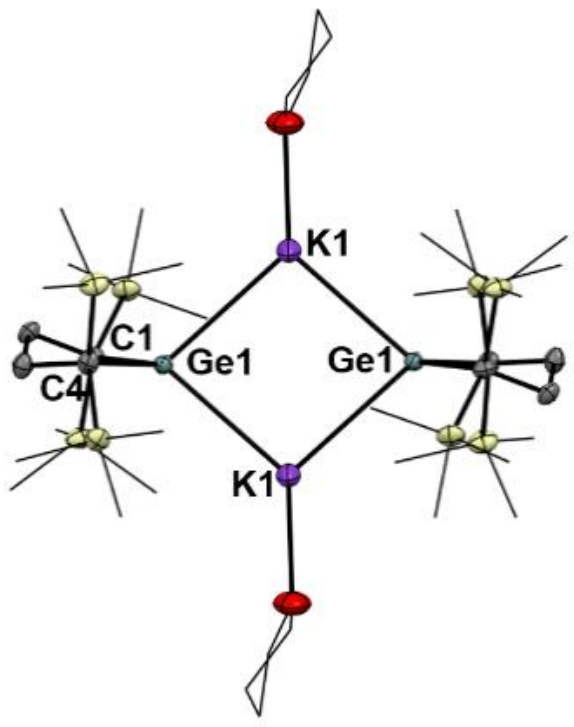

Fig. S29. Molecular structure of compound **2** in the crystal (thermal ellipsoids at 50% probability; all hydrogen atoms are omitted for clarity). Selected bond lengths (Å) and angles (°): Ge1–C4 2.062(4), Ge1–C1 2.083(4), Ge1–K1 3.367(12), C4–Ge1–C1 90.740(15), C4–Ge1–K1 129.540(1), C1–Ge1–K1 117.350(1).

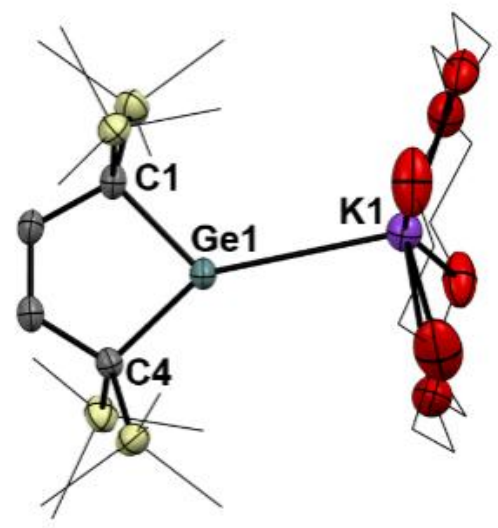

Fig. S30. Molecular structure of compound **3**·K(18-C-6) in the crystal (thermal ellipsoids at 50% probability; all hydrogen atoms are omitted for clarity). Selected bond lengths (Å) and angles (°): Ge1–C4 2.100(3), Ge1–C1 2.111(3), Ge1–K1 3.557(9), C4–Ge1–C1 89.380(1).

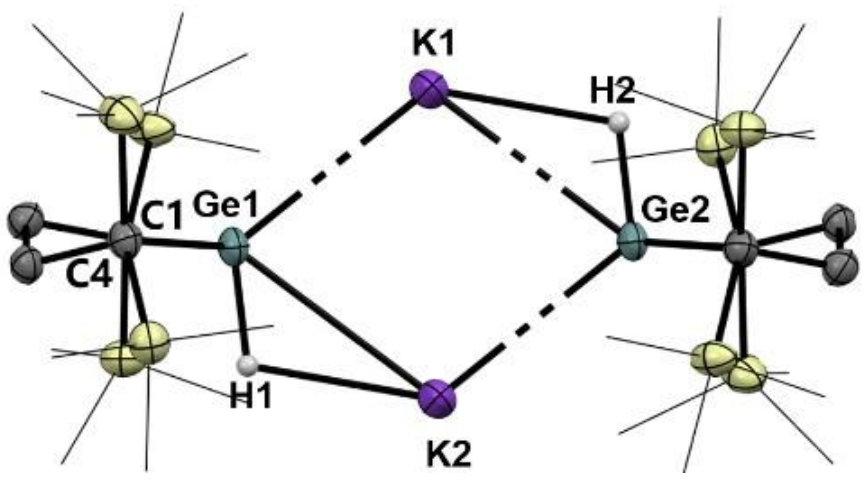

Fig. S31. Molecular structure of compound **4** in the crystal (thermal ellipsoids at 50% probability; all hydrogen atoms are omitted for clarity). Selected bond lengths (Å) and angles (°): Ge1–K1 3.382(8), Ge1–C1 2.077(3), Ge1–H1 1.500(4), K1–H1 2.680(5), K1–Ge1–K2 75.764(1), K1–Ge1–H1, 125.900(1), C1–Ge1–K1 123.390(6), C1–Ge1–C4 91.310(9), C1–Ge1–H1 99.600(1).

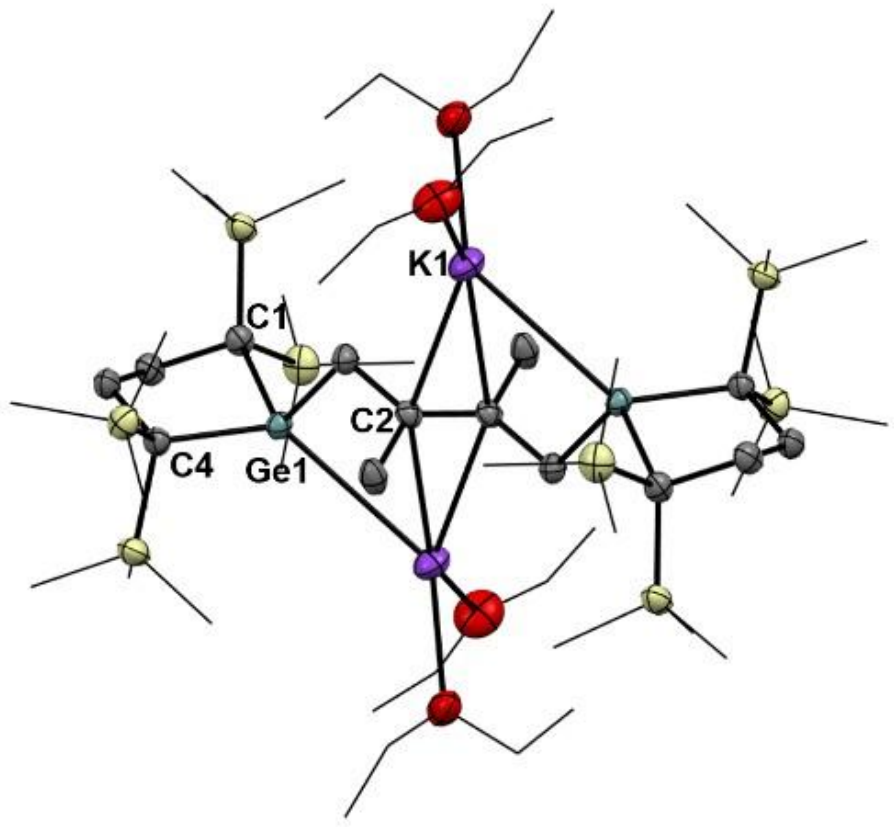

Fig. S32. Molecular structure of compound **5** in the crystal (thermal ellipsoids at 50% probability; all hydrogen atoms are omitted for clarity). Selected bond lengths (Å) and angles (°): Ge1–K1 3.371(5), Ge1–C1 2.105(1), Ge1–C4 2.126(1), K1–C2 3.226(3) , C1–Ge1–C4 109.780(5).

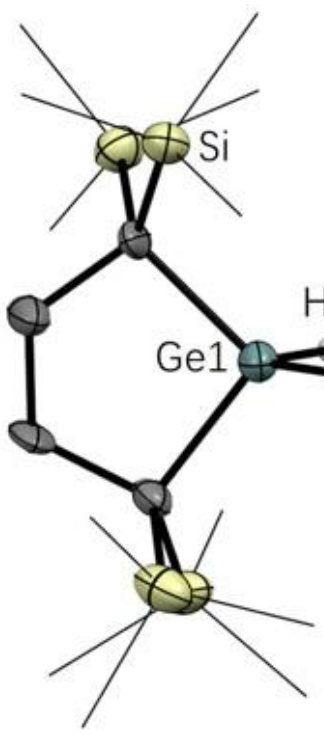

Fig. S33. Molecular structure of compound **6** in the crystal (thermal ellipsoids at 50% probability; all hydrogen atoms are omitted for clarity). Selected bond lengths (Å) and angles (°): Ge1–C1 1.988(8), Ge1–H1 1.520(5), C1–Ge1–C4 96.700(3), C1–Ge1–H1 108.800(2).

Table S3. Selected bond lengths (Å) and angles (°) of compounds **2-5** (experiment data fromXRD, computed data calculated at the BP86-D3(BJ)/def2-TZVP level of theory).

	**2**(exp)	**3**·K(18-C-6) (exp)	**3 (calc)**	**3**·K(18-C-6) (calc)	**4**(exp)	**5**(exp)
Ge1–C1	2.083(4)	2.111(3)	2.093	2.129	2.077(3)	2.105(1)
Ge1–C4	2.062(4)	2.100(3)	2.101	2.127	2.080(2)	2.126(1)
Ge1–K1	3.367(12)	3.557(9)	–	3.435	3.382(8)	3.371(5)
Ge1–H1	–	–	–	–	1.500(4)	–
K1–H1	–	–	–	–	2.680(5)	–
K1–C2	–	–	–	–	3.226(3)	–
C4–Ge1–C1	90.740(15)	89.380(12)	89.167	88.281	91.310(9)	109.780(5)
C1–Ge1–K1	117.350(1)	–	–	–	–	–
C4–Ge1–K1	129.540(1)	–	–	–	–	–
K1–Ge1–K2	83.22(3)		–	–	75.76 (6)	–
K1–Ge1–H1	–	–	–	–	99.600(1)	–
C1–Ge1–H1	–	–	–	–	99.600(1)	–

9. Computational Details

All calculations were carried out by using the Gaussian16 (G16 Revision. A03 version of the Gaussian program)[3] suite of computational programs. Geometry optimization was achieved using UBP86-D3(BJ)[4-5] functional. The standard def2-TZVP[6] basis set was applied for all atom. Frequency calculations were also performed at the same theoretical level to obtain vibrational frequencies and to verify the minimum-energy structure (with no imaginary frequencies). The most stabilized conformation was reported herein, when several different conformations were obtained. Excited states were calculated at the level at wB97XD-D3(BJ)[7]/def2-TZVP. All ground and excited states calculations were performed under the IEFPCM[8] (THF) implicit solvent model. Electron excitation analysis was performed using the wavefunction analysis software Multiwfn (version 3.8)[9] to determine the contributions of each orbital during the excitation process. Orbital isosurface diagrams were visualized using VMD.[10] Cartesian coordinates are provided in Source Data in this paper.

Table S4. TD[IEFPCM(THF)/wB97XD-D3(BJ)/def2-TZVP//BP86-D3(BJ)/def2-TZVP] results for germanium radical anion **3**. Wavelength (λ), oscillator strength (f) and main assignment.a

	λ(nm)	f	Assignment
1	454.64	0.00600	HOMO (β) → LUMO (β) 95.5%

[a] Major orbital transition contributions in excited states.

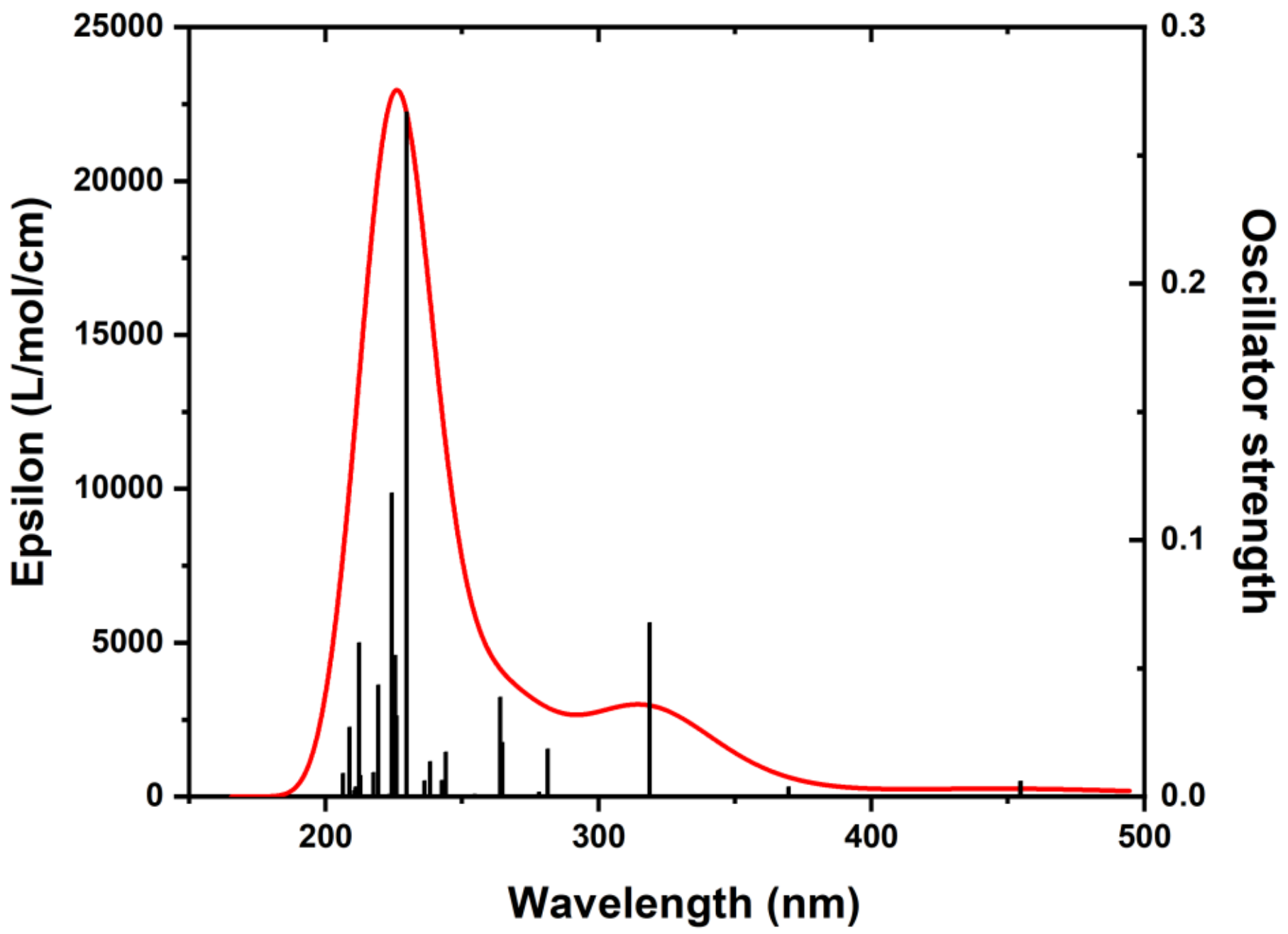

Fig. S34. Calculated UV-vis spectrum of germanium radical anion **3**. in tetrahydrofuran solution (red trace: Epsilon, black trace: Oscillator strength; 30 states calculated at the theory level of TD/IEFPCM(THF)/wB97XD-D3(BJ)/def2-TZVP//BP86-D3(BJ)/def2-TZVP).

Table S5. TD[IEFPCM(THF)/wB97XD-D3(BJ)/def2-TZVP//BP86-D3(BJ)/def2-TZVP] results for **3**·K(18-C-6).. Wavelength (λ), oscillator strength (f) and main assignment.a

	λ(nm)	f	Assignment
1	447.71	0.00510	HOMO (β) → LUMO (β) 87.1%<br>HOMO (β) → LUMO+1 (β) 8.0%

[a] Major orbital transition contributions in excited states.

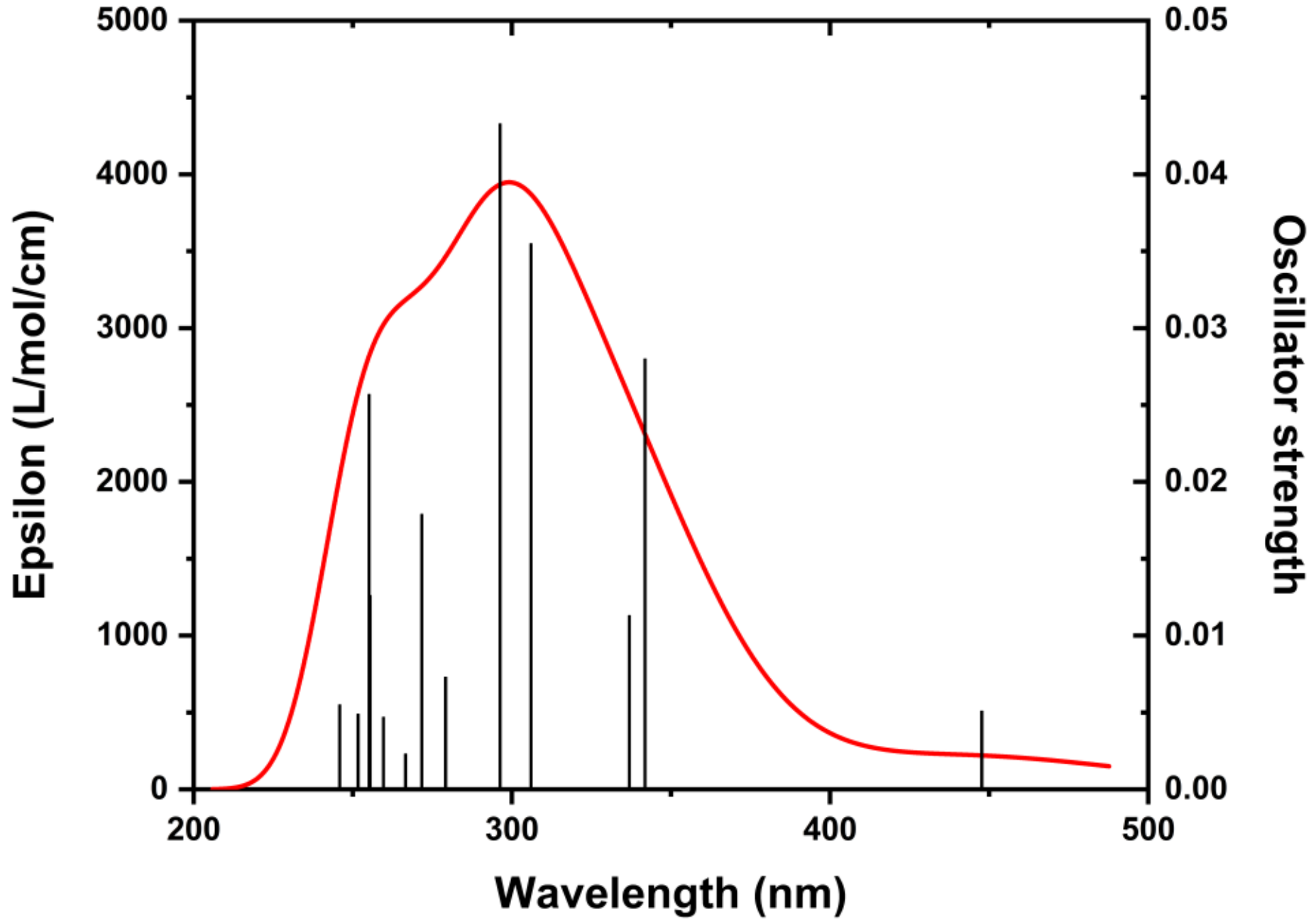

Fig. S35. Calculated UV-vis spectrum of **3**·K(18-C-6) in tetrahydrofuran solution (red trace: Epsilon, black trace: Oscillator strength; 30 states calculated at the theory level of TD/IEFPCM(THF)/wB97XD-D3(BJ)/def2-TZVP//BP86-D3(BJ)/def2-TZVP).

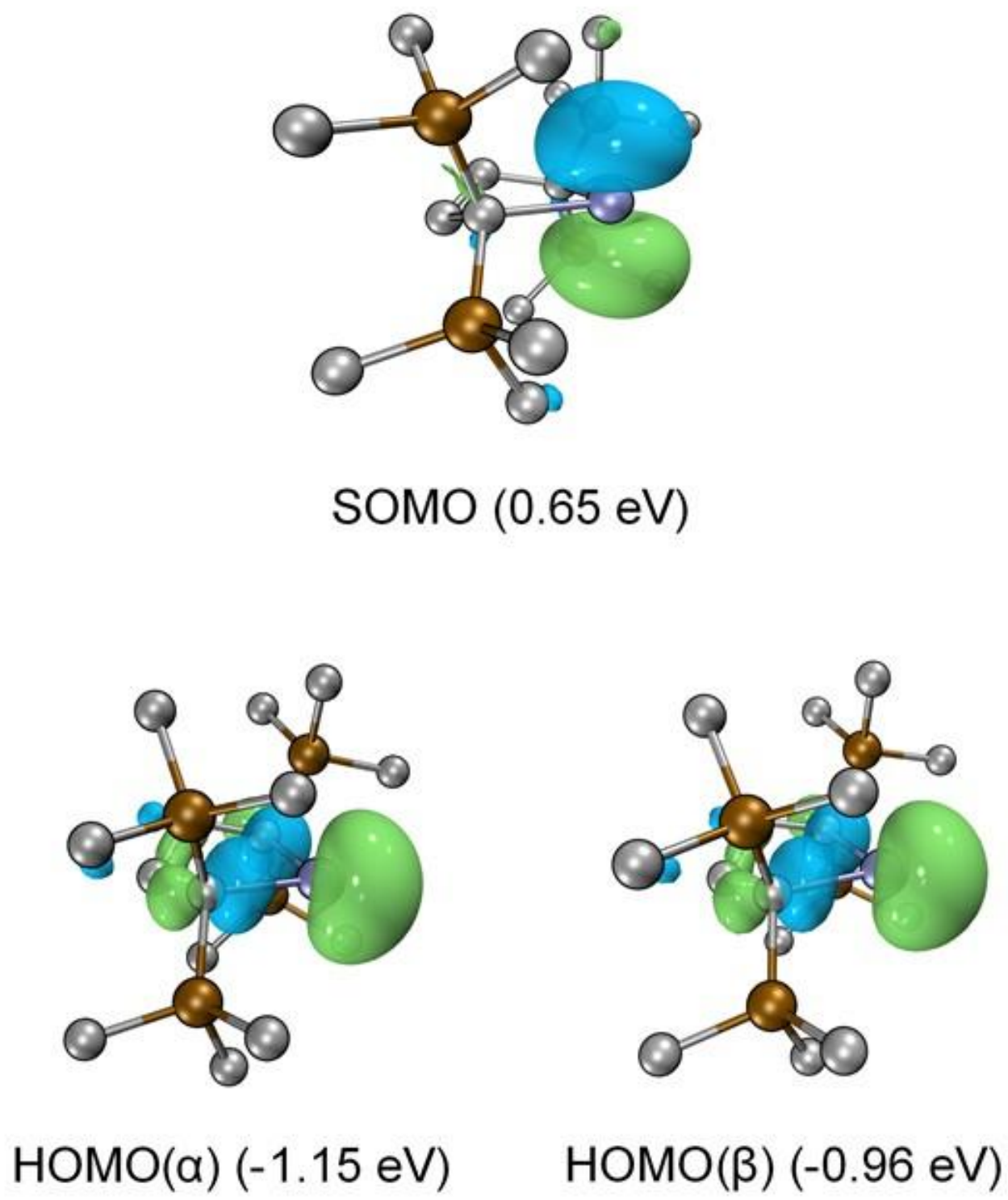

Fig. S36. Selected frontier orbitals of germanium radical anion **3** (isovalue = 0.05; Energies are given in the parentheses).

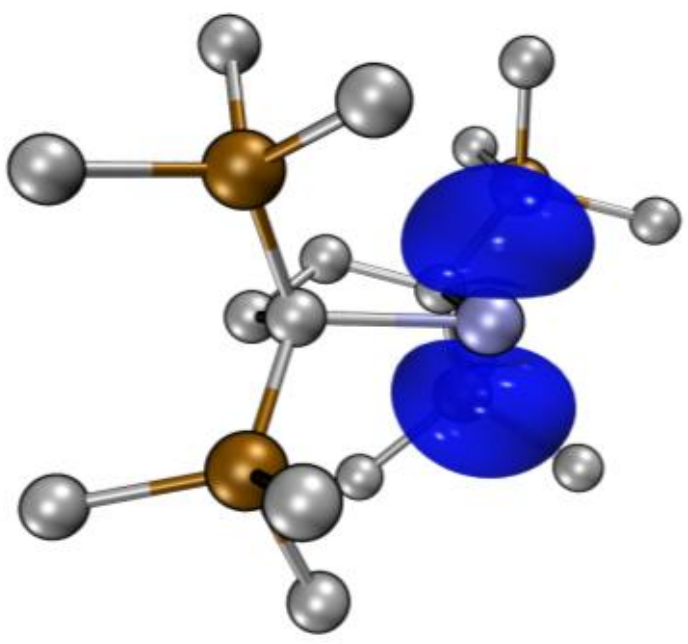

Fig. S37. Spin density of germanium radical anion **3** (isovalue = 0.005).

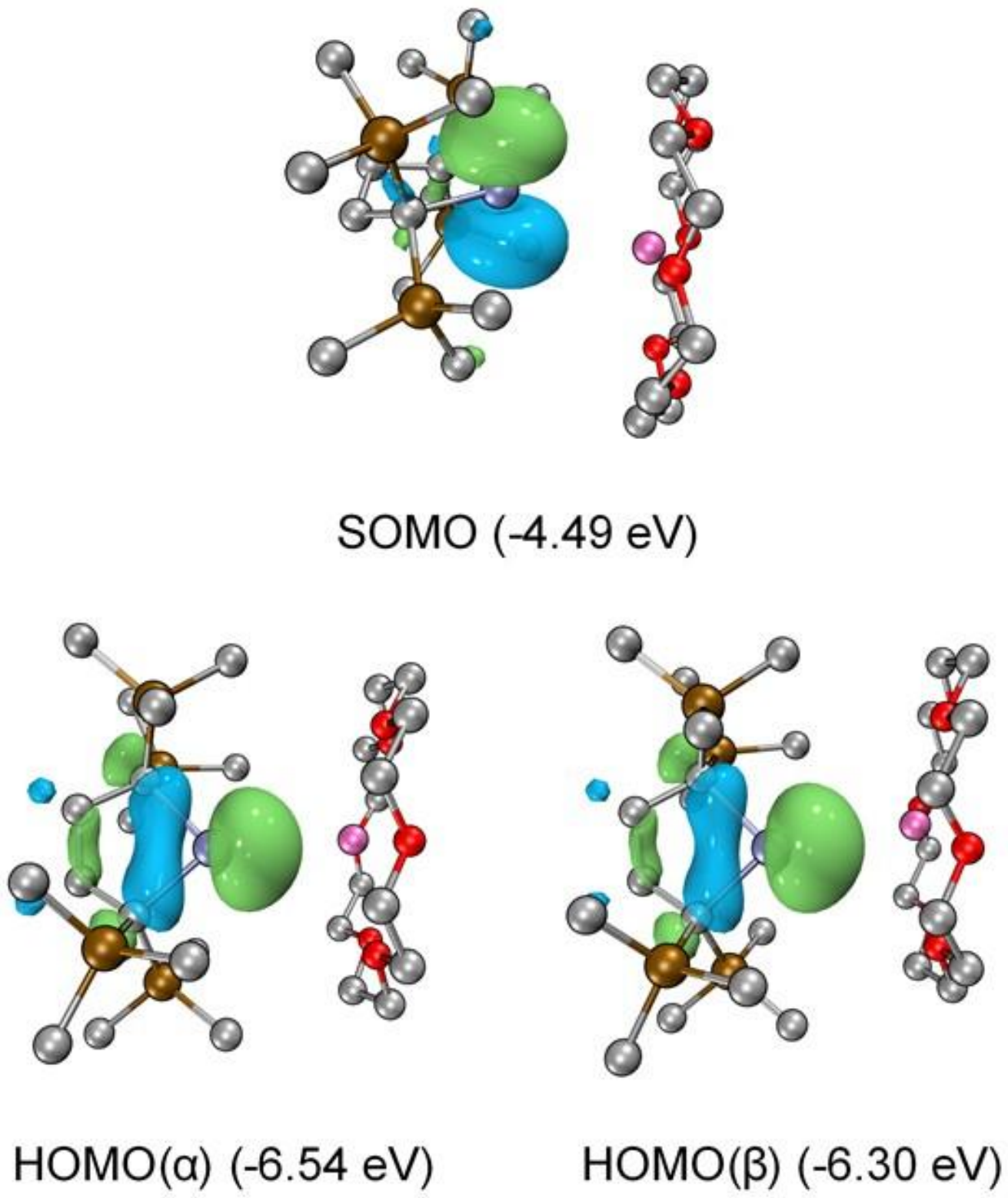

Fig. S38. Selected frontier orbitals of **3**·K(18-C-6) (isovalue = 0.05; Energies are given in the parentheses).

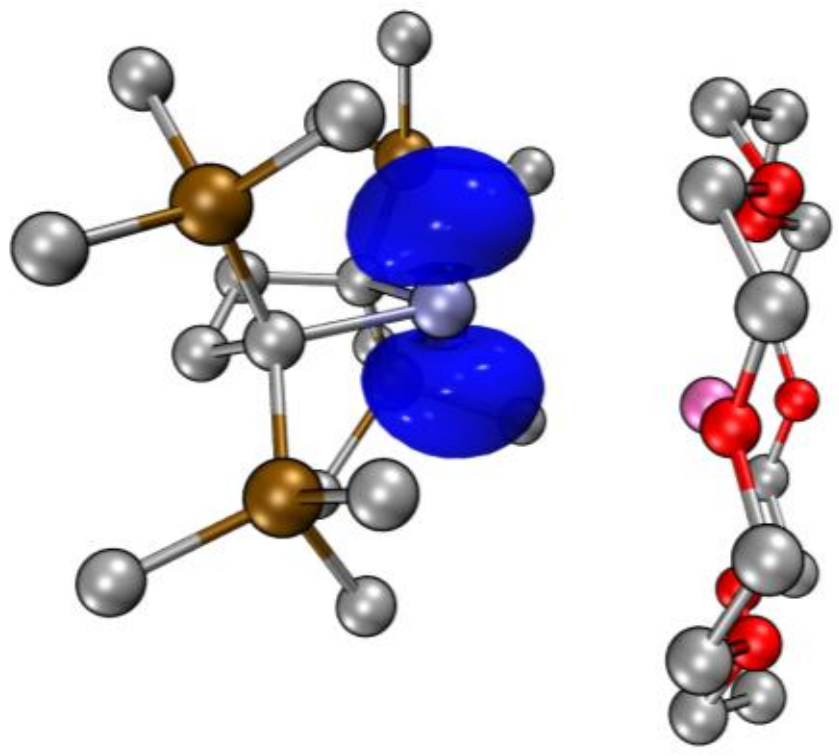

Fig. S39. Spin density of **3**·K(18-C-6) (isovalue = 0.005).

10. Supplementary Reference

1. Kira, M. *et al*. Synthesis and Structure of a Stable Cyclic Dialkylgermylene. *Chem. Lett.* **28**, 263-264(1999).
2. Mitchell. T. N. & Nikonov, G. Hexabutyldistannane. in *Encyclopedia of Reagents for Organic Synthesis*. (John Wiley & Sons, Inc., 2012).
3. Frisch, M.J., Trucks, G.W., Schlegel, H.B., Scuseria, G.E., Robb, M.A., Cheeseman, J.R.; Scalmani, G.; Barone, V.; Petersson, G.A.; Nakatsuji, H.; Li, X.; Caricato, M.; Marenich, A.V.; Bloino, J., Janesko, B.G., Gomperts, R., Mennucci, B., Hratchian, H.P., Ortiz, J.V., Izmaylov, A.F., Sonnenberg, J.L., Williams-Young, D., Ding, F., Lipparini, F., Egidi, F., Goings, J., Peng, B., Petrone, A., Henderson, T., Ranasinghe, D., Zakrzewski, V.G., Gao, J., Rega, N., Zheng, G., Liang, W., Hada, M., Ehara, M., Toyota, K., Fukuda, R., Hasegawa, J., Ishida, M., Nakajima, T., Honda, Y., Kitao, O., Nakai, H., Vreven, T., Throssell, K., Montgomery Jr., J.A., Peralta, J.E., Ogliaro, F., Bearpark, M.J., Heyd, J.J., Brothers, E.N., Kudin, K.N., Staroverov, V.N., Keith, T.A., Kobayashi, R., Normand, J., Raghavachari, K., Rendell, A.P., Burant, J.C., Iyengar, S.S., Tomasi, J., Cossi, M., Millam, J.M., Klene, M., Adamo, C., Cammi, R., Ochterski, J.W., Martin, R.L., Morokuma, K., Farkas, O., Foresman, J.B. & Fox, D.J. *Gaussian 16*, Wallingford CT, 2016.
4. Grimme. S. Accurate description of van der Waals complexes by density functional theory including empirical corrections. *J. Comput. Chem.* **25**, 1463–1473 (2004).
5. Perdew. J. P. Density-functional approximation for the correlation energy of the inhomogeneous electron gas. *Phys. Rev. B*. **33**, 8822–8824 (1986).
6. Weigend. F. & Ahlrichs. R. Balanced basis sets of split valence, triple zeta valence and quadruple zeta valence quality for H to Rn: Design and assessment of accuracy. *Phys. Chem. Chem. Phys.* **7**, 3297–3305 (2005).
7. Jeanmairet. G., Levesque. M. & Borgis. D. Molecular density functional theory of water describing hydrophobicity at short and long length scales. *J. Chem. Phys.* **139**, 154101 (2013).

8. Miertuš. S., Scrocco. E. & Tomasi. J. Electrostatic interaction of a solute with a continuum. A direct utilizaion of AB initio molecular potentials for the prevision of solvent effects. *Chem. Phys.* **55**, 117–129 (1981).
9. Lu. T. & Chen. F. W. Multiwfn: A Multifunctional Wavefunction Analyzer. *J. Comput. Chem.* **33**, 580−592 (2012).
10. Humphrey. W., Dalke. A. & Schulten. K. VMD - Visual Molecular Dynamics. *J. Mol. Graphics.* **14**, 33−38 (1996).
