# Supplementary material for: Isolation and reactivity of an alkyl-substituted germanium(I) radical anion
Source: Nat Commun. 2026 Jun 19;17:8251. doi: 10.1038/s41467-026-74699-1 (PMC13462986; doi:10.1038/s41467-026-74699-1)
Supplement: Supplementary file 2 [file 41467_2026_74699_MOESM2_ESM.pdf]

nature portfolio

Peer Review File

Isolation and Reactivity of an Alkyl-Substituted Germanium(I) Radical Anion

Corresponding Author: Dr Chenting Yan

This file contains all reviewer reports in order by version, followed by all author rebuttals in order by version.

Version 0:

Reviewer comments:

Reviewer #1

(Remarks to the Author)
Reviewer's Report: NCOMMS-26-014639

This is an excellent report detailing the synthesis, isolation, characterisation and reactivity of the second isolable Ge(I) anion and the first bearing only carbon ancillary ligands. The chemistry described within is of very high quality, the compounds are mostly well characterised and it was nice to see how these compounds react.

The chemistry mimics that of previously reported dialkyl silyl radical anion chemistry (ref 46), as well as uncited references of dialkyl silyl radical anions with a crown ether (Inoue, Ichinohe, Sekiguchi, JACS, 2003, 6096; Inoue, Ichinohe, Sekiguchi, Organometallics, 2008, 1358); as such, the comment in the abstract that "this work establishes a new paradigm for synthesizing challenging low-valent main group radical species" is disingenuous.
Despite this slight over-selling of this work, the synthesis of two isolable dialkyl substitute germanium radical anions is noteworthy. The evidence for the radicals is strong, and taken with the reported subsequent reactivity studies of these compounds, it makes this study worthy of publication in Nature Communications.

Queries, Manuscript:
- Is there evidence that the solid-state structure and solution-state structure of compounds 2 are the same?
- The colours reported within for compound 2 is inconsistent when discussing the UV-Vis data (ie "in line with the orange colour, the UV-Vis spectra of 2 and 3…").
- Although the authors mentioned compound 3 is thermally stable, the authors need to specify which state (ie solid state or solution) they are stable in.
- "exhibits exceptional thermal stability in both the solid state and in solution at ambient temperature"
- As usual, please do one more proofread check.

Queries, Experimental:
NOTE – I am confused as to why the experimental for only some of the compounds are in the article, whereas others are in the supporting information. Please check the guidelines for this.
General:
- Wording change: The glassware used (not the used glassware)
- Wording change: All standard chemicals used (not used standard chemicals)
- The synthesis of compounds 1 and 7 points to reference 7; however, this manuscript only describes the synthesis of dialkylsilylene and not compounds 1 and 7. Please amend appropriately.
Compound 2:
- The authors generate radical anions using 2 different strategies. As is the nature of these compounds, traditional characterisation techniques (ie 1H NMR spectroscopy) are not as useful due to the presence of a radical. The "red compound" is consistent with that formed via the two techniques, but there is no evidence that these are the same compounds. The authors need to provide more evidence as to why they believe that Method 1 and 2 resulted in the formation of the same compound. This could be as simple as a unit cell check of isolated crystals or HRMS (ESI) for both compounds.
Compound 3:
- The authors generate radical anions using 2 different strategies. Please see comments for compound 2 and provide more

evidence that the orange solids isolated in all three strategies are the same compound.
UV-Vis spectroscopy:
- The authors need to report the extinction coefficient for compounds 2 and 3.
- I am surprised about the broadness of the UV-Vis spectra as this leads me to wonder if these compounds are stable in solution as the UV-Vis spectra for the germanium radical reported in reference 52 is sharp. Indeed, reference 65 reports that the max for the radical anion (666 nm) is red-shifted compared to by over 200 nm compared to the neutral compounds (410 nm), where as only a very small red-shift is observed in this manuscript. It is noted that it can be challenging to obtain a reliable UV-Vis spectrum of a highly sensitive and highly coloured compound in THF due to the presence of impurities within the THF and the dilute conditions required.
- The UV-Vis spectra needs to be repeated under very rigorous conditions, and if sharper peaks are not found, then an explanation for this should be included.
- A comparison between the calculated and experimental UV-Vis data needs to be included.
- The UV-Vis spectra and the calculated UV-Vis spectra do not match – please increase the wavelength range of the spectra to the THF cutoff (ie 220 nm) to see if greater match is possible. If not, then please provide and explanation for the difference.
Compound 6 experimental:
- State how much water was added.
Compound 4
- The authors need to refer the “crude product” as “crude reaction mixture” as the former implies an isolated compound.
Compound 5:
- Change wording from “was decomposed in acetonitrile” to “decomposed in acetonitrile”
Compound 8-D2
- Is it possible to run a 2H NMR spectrum of this compound to confirm the deuterium signal?

References:
- Reference 65 is for the correction and not the actual manuscript.

Reviewer #2

(Remarks to the Author)
Yan et al. report on a new germanium radical anion that is stable at room temperature and could hence be isolated and fully characterized including single-crystal X-ray diffraction. The findings add to current interest in low-valent main-group chemistry and especially radical compounds hold much potential. The work has been conducted at high level and the results presented in here are convincing.

While radical anions of germanium have been reported before, they are typically unstable at ambient conditions. Using a cyclic ligand environment, the authors were able to stabilize the formal Ge(I) centre to a certain extent thus the compound is stable at room temperature. The radical anion could be isolated and characterized in two forms, i.e., a potassium bridged dimer and a monomer through complexation of 18-crown-6. Both, experiment and theory provide evidence that the spin density is mainly located at germanium. Additional reactivity studies have been conducted and underpin the properties of the radical anion. This is a very nice and timely piece of work. I am sure it will receive broad attention and is hence well suited for the readership or Nature Communications. I fully support acceptance and have only a few minor comments:

The statement “solely by steric protection, without electronic perturbations” appears confusing. Whenever a ligand is used, both steric and electronic effects play a role. Why that should not be the case here?

The reactivity section falls very short. Give the nice results I suggest that the authors add two or three paragraphs to discuss their findings in more detail. Especially compound 4 and 5 deserve this.

Figure 5: using a, b, c, and d is confusing please move the reagents from the caption to the figure and add yields.

IR-spectra of compound 4-6 could be very insightful.

As the compounds are colourful, I suggest measuring the UV-vis data and maybe comparing them with the computations.

Compound 5 could not be fully characterized due to decomposition in acetonitrile. I do not why the instrument runs only with acetonitrile as these instruments can be used with almost every solvent. Hence, an additional technique should be applied like elemental analysis.

The deviation of the HRMS results is in some cases too high. This should be checked and revised.

Reviewer #3

(Remarks to the Author)
In this work, Yan and co-workers report the synthesis, characterization, and reactivity of a cyclic dialkyl Ge(I) radical anion generated via reduction of the corresponding germylene or dichlorogermane precursors. Isolable Ge(I) radical anions remain exceedingly rare, with prior examples essentially limited to the system reported by Hicks et al. (compound II in the manuscript). In this regard, the present study expands the small family of structurally authenticated Ge(I) radical anions.

The reported species is new within the germanium radical anion context. However, it should be noted that the underlying ligand framework has previously been employed to stabilize related main-group radical species, including a persistent dialkylsilylene radical anion reported as early as 2003, as well as isolable phosphinyl radicals and related open-shell systems. Consequently, while the work represents a solid incremental advance, its conceptual novelty appears limited. In my opinion, the study does not fully meet the level of conceptual innovation and general significance typically expected for publication in Nature Communications.

Several issues should also be addressed to strengthen the manuscript:

1. The authors compare radical anion II with alkyl-substituted Ge(I) radical anions and conclude that the latter are electronically non-perturbing. However, the manuscript does not clearly explain how the diamido ligand perturbs the electronic structure of compound II. A more explicit analysis—supported by orbital descriptions, spin-density distributions, or quantitative bonding metrics—would be necessary to substantiate this claim.

2. In compound II, describing the ligand as an N,O-donor may be misleading. Structurally and electronically, the ligand functions more appropriately as a diamido ligand. The terminology should therefore be revised for accuracy and consistency with established conventions.

3. The EPR measurements reveal notable spin–nuclear coupling with silicon nuclei. It would therefore be important to clarify whether the computational analysis identifies any significant orbital interaction or spin delocalization between the silyl substituents and the germanium center. Discussion of relevant molecular orbitals, spin populations, or hyperfine coupling calculations would help rationalize the experimental observations.

Overall, the manuscript presents careful experimental work and valuable characterization data. Addressing the points above would improve clarity and strengthen the mechanistic and electronic interpretations; however, in its current form, the advance appears incremental relative to prior literature.

Version 1:

Reviewer comments:

Reviewer #1

(Remarks to the Author)
I am satisfied with the authors comments and corrections. This is suitable for publication.

Reviewer #2

(Remarks to the Author)
The authors provided a revised version of their initial submission and a very detailed response, which is appreciated. The points raised by the reviewers have been addressed properly and the majority of the reviewers considers the work suitable for publication in Nature Communications. Indeed, there is precedence but there is also interesting reactivity and, in my eyes, enough novelty to warrant publication in Nature Communication. Hence, I recommend publication and have only very few formal comments to be addressed:

Abstract: Please remove “exceptional” in the sentence “This radical anion exhibits exceptional thermal stability in both the solid state and in solution” as it is too much over-selling.

Figure 2a and S1: Please add an arrow that indicates the direction of scanning the voltage.

Figure 2b: I would include the synthetic route starting from cmpd. 7 as this is the most suitable and high-yielding route towards 2.

Figure 3 & 5: Please add “in the solid state” as these are molecular solid-state structures

Figure 5: Please add information on the probability and which atoms you omitted

Reviewer #3

(Remarks to the Author)
The authors have properly addressed all the issues. I support the publication of this manuscript in Nat. Commun.

Open Access This Peer Review File is licensed under a Creative Commons Attribution 4.0 International License, which permits use, sharing, adaptation, distribution and reproduction in any medium or format, as long as you give appropriate credit to the original author(s) and the source, provide a link to the Creative Commons license, and indicate if changes were made.
In cases where reviewers are anonymous, credit should be given to 'Anonymous Referee' and the source.
The images or other third party material in this Peer Review File are included in the article's Creative Commons license, unless indicated otherwise in a credit line to the material. If material is not included in the article's Creative Commons license and your intended use is not permitted by statutory regulation or exceeds the permitted use, you will need to obtain permission directly from the copyright holder.
To view a copy of this license, visit https://creativecommons.org/licenses/by/4.0/

Hangzhou, May 15, 2026

Dear Reviewers,

We sincerely appreciate the detailed feedback and the positive and helpful comments provided by the reviewers on our manuscript. The guidance offered has been instrumental in enhancing the quality and clarity of our work. We have carefully considered the reviewer's comments and have made the necessary revisions to improve the article. The following changes have been made:

Reviewer 1:

General comments: This is an excellent report detailing the synthesis, isolation, characterisation and reactivity of the second isolable Ge(I) anion and the first bearing only carbon ancillary ligands. The chemistry described within is of very high quality, the compounds are mostly well characterised and it was nice to see how these compounds react.

The chemistry mimics that of previously reported dialkyl silyl radical anion chemistry (ref 46), as well as uncited references of dialkyl silyl radical anions with a crown ether (Inoue, Ichinohe, Sekiguchi, JACS, 2003, 6096; Inoue, Ichinohe, Sekiguchi, Organometallics, 2008, 1358); as such, the comment in the abstract that "this work establishes a new paradigm for synthesizing challenging low-valent main group radical species" is disingenuous.

Despite this slight over-selling of this work, the synthesis of two isolable dialkyl substitute germanium radical anions is noteworthy. The evidence for the radicals is strong, and taken with the reported subsequent reactivity studies of these compounds, it makes this study worthy of publication in *Nature Communications*.

Response: We thank the reviewer for the positive evaluation and strong support of this work. We apologize for the oversight of the important precedent by Inoue, Ichinohe, Sekiguchi (*J. Am. Chem. Soc.* **2007**, *129*, 6096; *Organometallics* **2008**, *27*, 1358) on isolable silylene anion radicals. These articles have now been properly cited in the revised manuscript (Refs. 48 and 49). Moreover, the sentence "this work establishes a new paradigm for synthesizing challenging low-valent main group radical species" has been corrected as "This work represents a rare example of an isolable low-valent group 14 radical species". To duly recognize the pioneering work by Prof. Sekiguchi et

al, we have revised the sentence on page 4: "Building upon the precedent of generating silicon radical anions via reduction of dialkylsilylene and tetrakis(di-*tert*-butylmethylsilyl)disilene,[46,48-49] ..."

Comment 1: Is there evidence that the solid-state structure and solution-state structure of compounds **2** are the same?

Response: Thank you for this insightful question. Based on literature precedents and our spectroscopic analysis, we think that compound **2** adopts different structures in the solid state and in solution.

In the solid state, compound **2** exists as a dimer, as established by single-crystal X-ray diffraction analysis. However, in solution, it is most likely present as a monomer confirmed by EPR study. This assignment is supported by a closely related example that an alkali-metal-substituted silyl radical was shown to adopt a dimeric structure in the solid state but dissociate into monomeric species in solution, as evidenced by both crystallographic and EPR studies (S. Inoue, M. Ichinohe, and A. Sekiguchi, Isolable Alkali-Metal-Substituted Silyl Radicals $(^{t}Bu_2MeSi)_2SiM$ (M = Li, Na, K): Electronically and Sterically Accessible Planar Silyl Radicals, *Organometallics*, **2008**, *27*, 1358).

Comment 2: The colours reported within for compound **2** is inconsistent when discussing the UV-Vis data (ie "in line with the orange colour, the UV-Vis spectra of **2** and **3**…").

Response:

We thank the reviewer for this important observation. We agree that the description of the color of compound **2** in relation to the UV–Vis data was potentially misleading.

During the UV–Vis measurements, we noted a clear concentration-dependent color change: while compound **2** appears dark red in the solid state and in concentrated solutions, dilution leads to an orange-yellow solution. To further clarify this behavior, we regrew single crystals of compound **2** from *n*-hexane under slow evaporation conditions to obtain crystals with different thicknesses. We found that thinner crystals appear orange-yellow, whereas thicker crystals display a deeper color. This observation indicates that the perceived color is strongly influenced by optical path length and concentration-dependent absorption, rather than a change in the intrinsic electronic transition. In addition, we have re-measured the UV–Vis spectra under various conditions (in THF, Et_2O and *n*-hexane). The corresponding spectra at different concentrations and in different solvents were presented and discussed in our

responses to the comment 13 related to the UV–Vis data. The description of the color of compounds **2** and **3** has been revised accordingly.

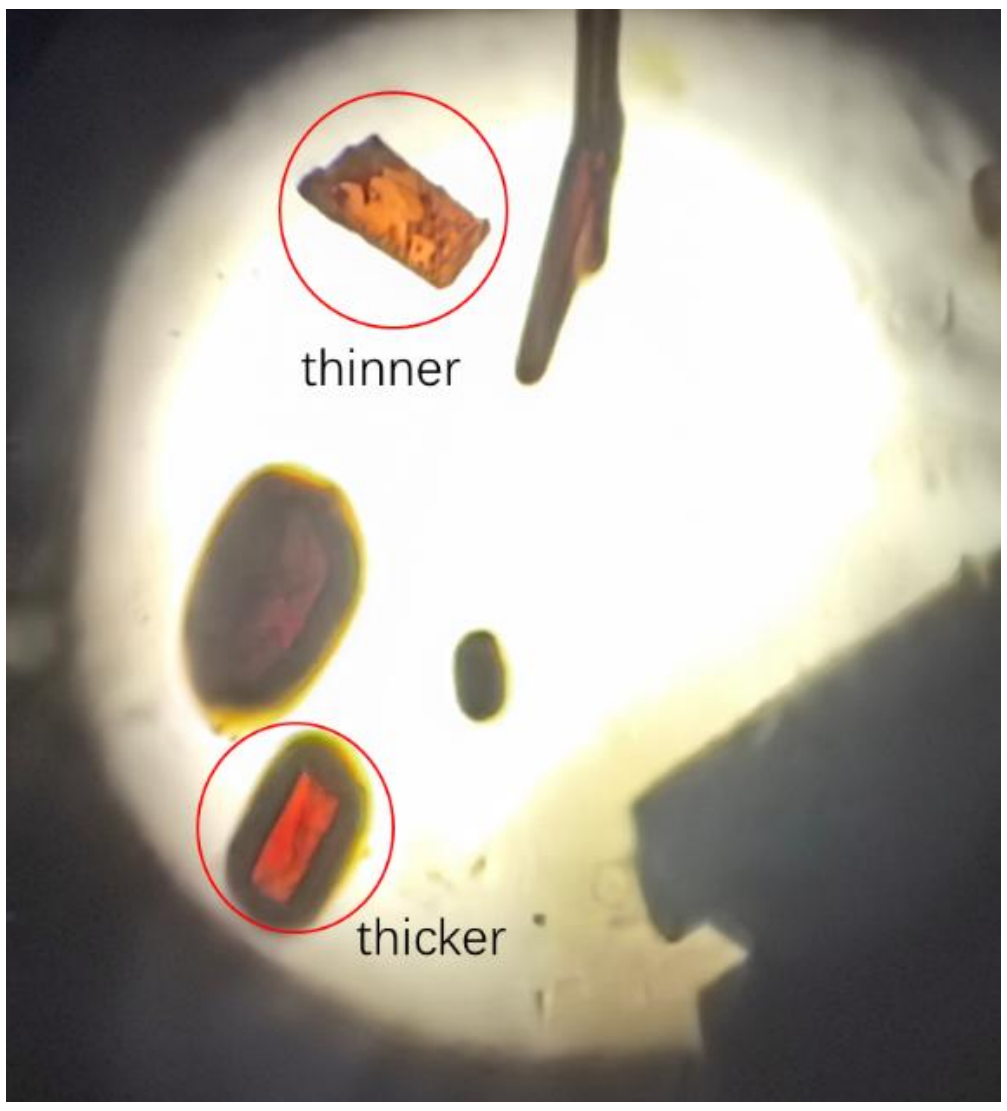

Comment 3: Although the authors mentioned compound **3** is thermally stable, the authors need to specify which state (ie solid state or solution) they are stable in.

Response: We thank the reviewer for this helpful suggestion. Compound **3** is thermally stable under an inert atmosphere in both the solid state and in solution. To clarify this point, we have revised the manuscript as follows:

"Compound **3**·K(18-C-6) is highly sensitive to air and moisture but exhibits remarkable thermal stability under inert conditions in both the solid state and in solution, showing no detectable decomposition for at least one month at room temperature."

Comment 4: "exhibits exceptional thermal stability in both the solid state and in solution at ambient temperature"

Response: We appreciate the reviewer for pointing out the wording error. We have corrected it accordingly.

Comment 5: As usual, please do one more proofread check.

Response: We thank the reviewer for this suggestion. We have carefully proofread the entire manuscript once again to improve clarity, grammar, and overall readability, and have made the necessary revisions throughout the text.

Comment 6: NOTE – I am confused as to why the experimental for only some of the compounds are in the article, whereas others are in the supporting information. Please check the guidelines for this.

Response: We thank the reviewer for pointing out this issue. In the revised manuscript, we have carefully checked the consistency between the main text and the Supporting Information, only the compound **7** was not explicitly indicated in the main text. We have now corrected this by labeling it as "cyclic dichlorogermane precursor 7" in the manuscript, ensuring that all compounds are consistently numbered and clearly referenced in both the main text and the Supporting Information.

Comment 7: Wording change: The glassware used (not the used glassware)

Response: We appreciate the reviewer for pointing out the wording error. We have corrected it accordingly.

Comment 8: Wording change: All standard chemicals used (not used standard chemicals)

Response: We appreciate the reviewer for pointing out the wording error. We have corrected it accordingly.

Comment 9: The synthesis of compounds **1** and **7** points to reference 7; however, this manuscript only describes the synthesis of dialkylsilylene and not compounds **1** and **7.** Please amend appropriately.

Response: Thank you for pointing this out. We have updated the reference to include the reported synthesis of compounds **1** and **7**, and the citation has been amended accordingly. "M., Kira, S., Ishida, T., Iwamoto, M., Ichinohe, C., Kabuto, L., Ignatovich, H., Sakurai, "Synthesis and Structure of a Stable Cyclic Dialkylgermylene" *Chem. Lett*. **1999**, *28*, 263-264."

Comment 10: Compound **2**: The authors generate radical anions using 2 different strategies. As is the nature of these compounds, traditional characterisation techniques (ie ^{1}H NMR spectroscopy) are not as useful due to the presence of a radical. The "red compound" is consistent with that formed via the two techniques, but there is no evidence that these are the same compounds. The authors need to provide more evidence as to why they believe that Method 1 and 2 resulted in the formation of the same compound. This could be as simple as a unit cell check of isolated crystals or HRMS (ESI) for both compounds.

Response: We thank the reviewer for this insightful comment. To verify whether Method 1 and Method 2 yield the same compound, we synthesized compound **2** via Method 2 and subsequently grew single crystals under identical conditions to those used in Method 1. After obtaining suitable crystals, we checked the unit cell, which showed identical crystallographic parameters to those obtained from Method 1. This confirms that both methods afford the same compound 2.

Compound	**2**	
	Method 1	Method 2
a, Å	10.8448(6)	10.87
b, Å	11.3293(6)	11.35
c, Å	13.4356(8)	13.49
α, deg	105.810(2)	105.81
β, deg	105.661(2)	105.63
γ, deg	100.719(2)	100.74
V, A^3	1468.10(14)	1481
Crystal syst	Triclinic	Triclinic
Space group	P-1	P-1

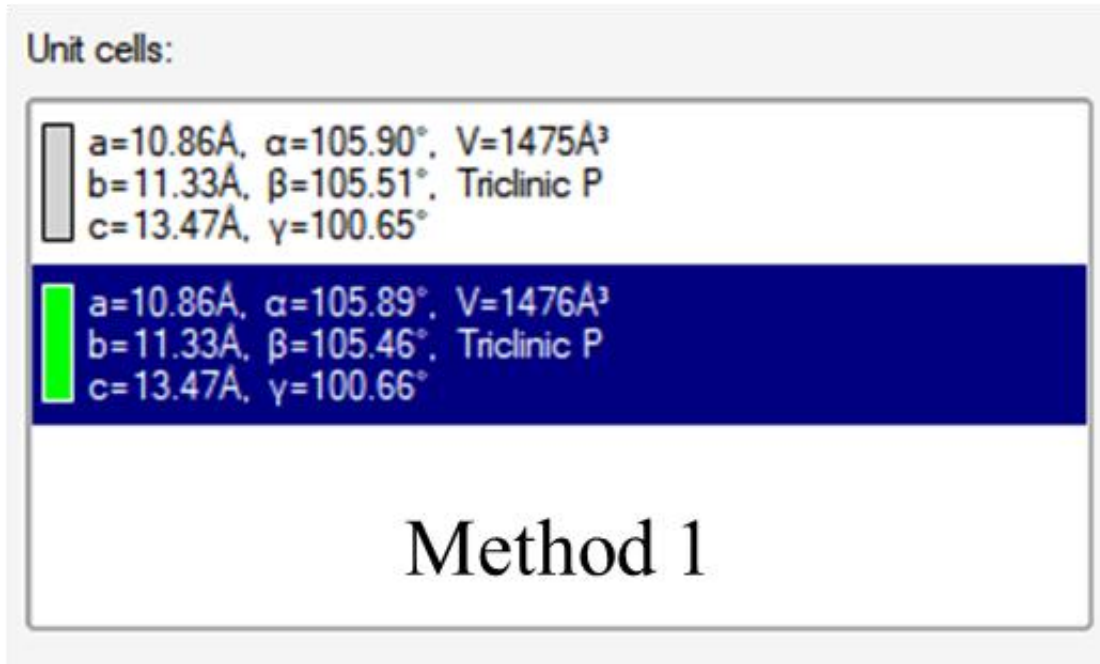

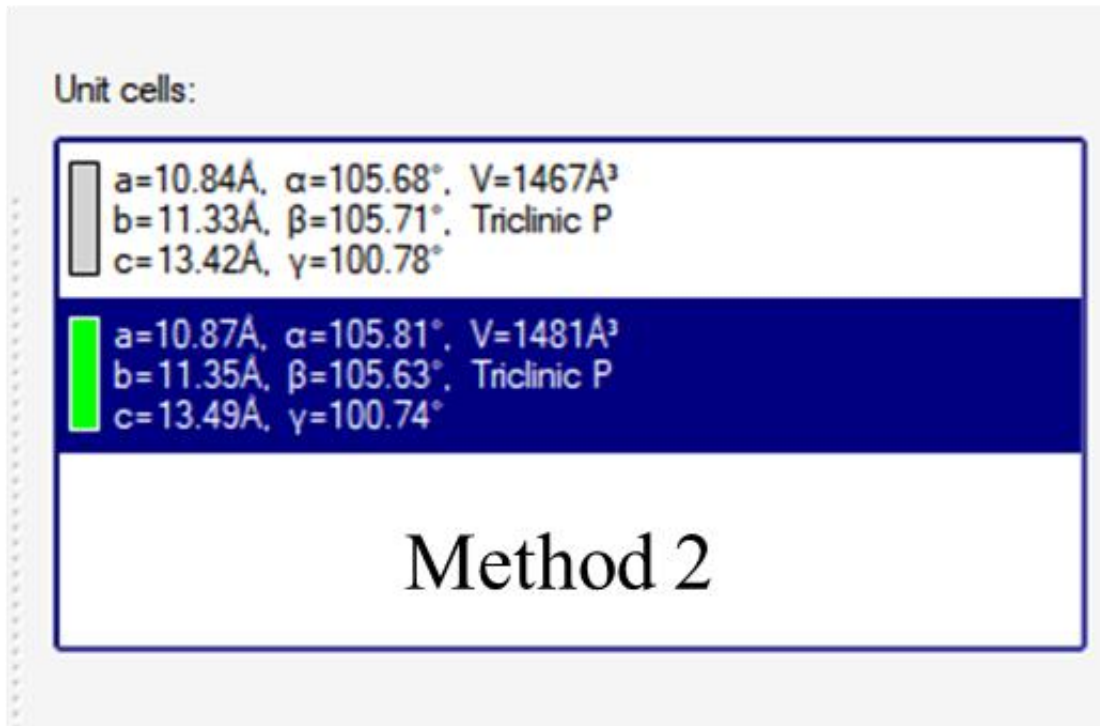

Comment 11: Compound **3**: The authors generate radical anions using **2** different strategies. Please see comments for compound **2** and provide more evidence that the orange solids isolated in all three strategies are the same compound.

Response: We thank the reviewer for this insightful comment. To address this concern, we synthesized compound **3** via Method 2 and Method 3, and subsequently grew single crystals under the same conditions as those used in Method 1. After obtaining suitable crystals, unit cell determinations revealed that the crystals from Method 2 and Method 3 differ from those obtained via Method 1. Specifically, the crystal from Method 1 crystallizes in a triclinic P space group, whereas those from Method 2 and Method 3 crystallize in a monoclinic C space group.

Despite these differences in crystallographic symmetry, preliminary analysis based on fast-scan diffraction data indicates that the structures still correspond to Compound **3**

(see the crystal structures below). Therefore, these results demonstrate that Methods 2 and 3 also afford Compound **3**, albeit in different crystal forms.

Compound	**3**·K(18-C-6)		
	Method 1	Method 2	Method 3
a, Å	11.0048(4)	22.1379(8)	22.17
b, Å	12.0426(6)	10.9847(4)	10.99
c, Å	33.1989(17)	49.560(2)	49.60
α, deg	82.180(2)	90.00	90.00
β, deg	87.994(2)	92.573(2)	92.61
γ, deg	66.680(2)	90.00	90.00
V, A^3	4001.9(3)	12039.7(8)	12077
Crystal syst	Triclinic	Monoclinic	Monoclinic
Space group	*P*-1	*C*2/*c*	*C*2/*c*

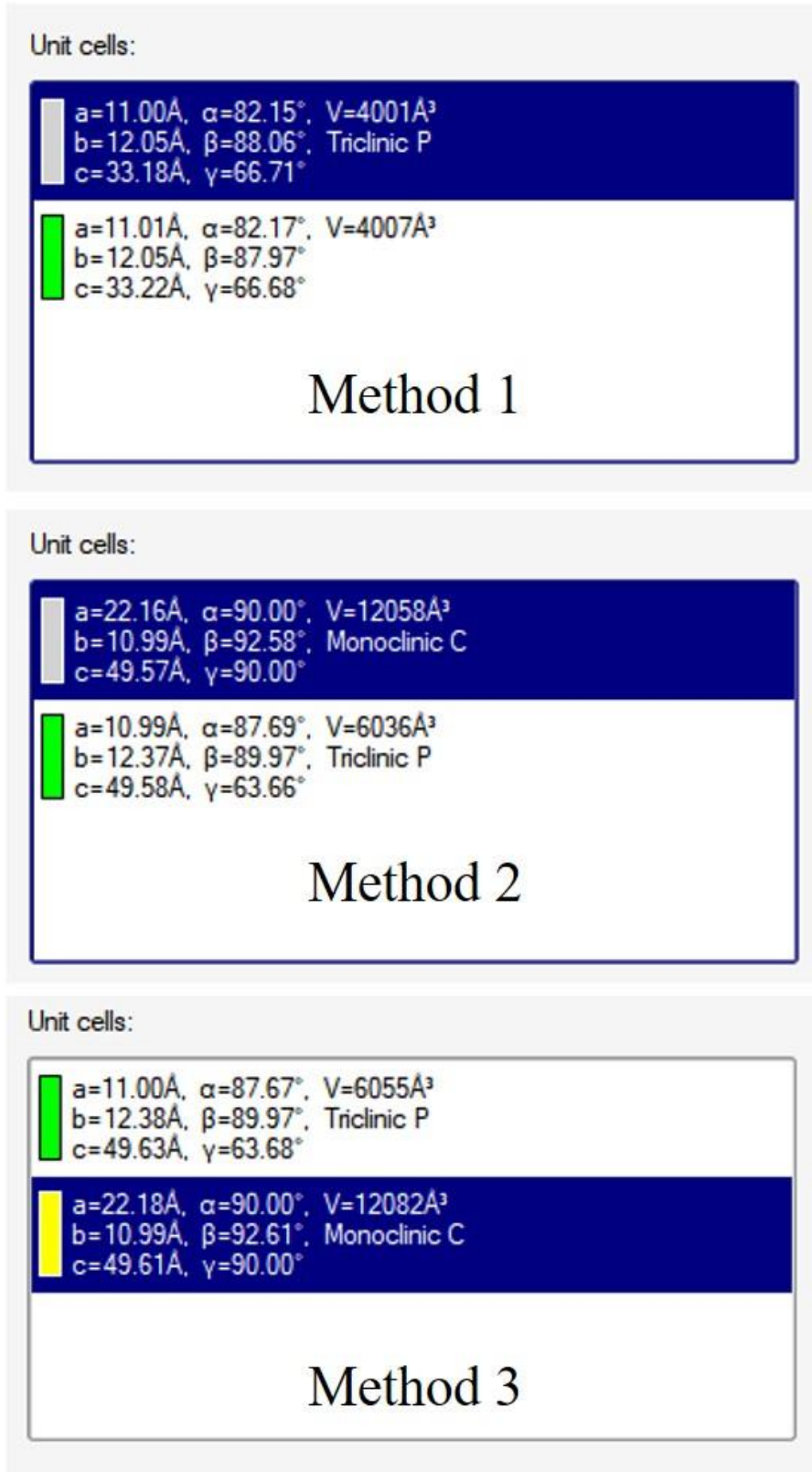

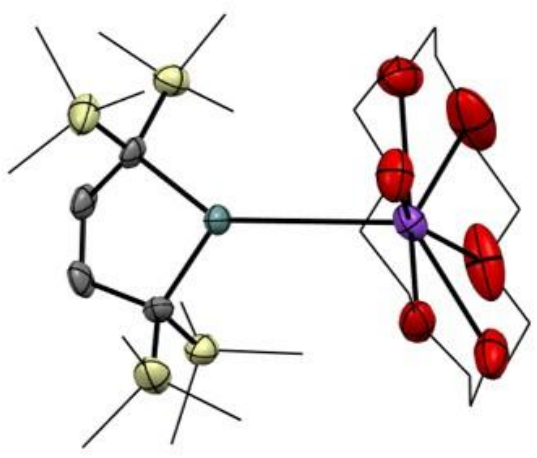
Crystal structure of Method 2

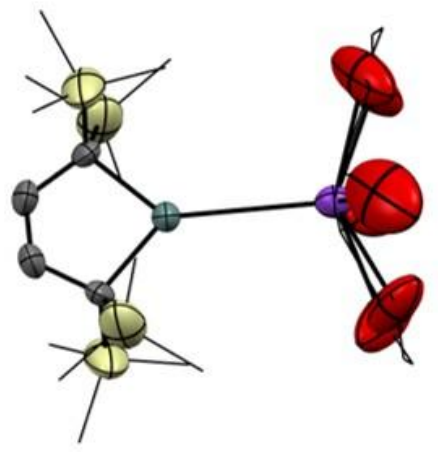
Crystal structure of Method 3

Comment 12: UV-Vis spectroscopy: The authors need to report the extinction coefficient for compounds **2** and **3**.

Response: We thank the reviewer for this helpful suggestion. The molar extinction coefficients (ε) for compounds **2** and **3** have now been determined based on UV–Vis measurements using a 1 cm path length cuvette, according to the Beer–Lambert law.

The calculated values are as follows:

Compound **2**: $\varepsilon = 6.25 \times 10^3$ L·mol^{-1}·cm^{-1} (λ_{max} = 464 nm, c = 1.0×10^{-4} M)

Compound **3**: $\varepsilon = 6.73 \times 10^2$ L·mol^{-1}·cm^{-1} (λ_{max} = 477 nm, c = 1.0×10^{-3} M)

These values have now been included in the revised manuscript.

Comment 13: I am surprised about the broadness of the UV-Vis spectra as this leads me to wonder if these compounds are stable in solution as the UV-Vis spectra for the

germanium radical reported in reference 52 is sharp. Indeed, reference 65 reports that the λmax for the radical anion (666 nm) is red-shifted compared to by over 200 nm compared to the neutral compounds (410 nm), where as only a very small red-shift is observed in this manuscript. It is noted that it can be challenging to obtain a reliable UV-Vis spectrum of a highly sensitive and highly coloured compound in THF due to the presence of impurities within the THF and the dilute conditions required.

Response: We thank the reviewer for this insightful comment regarding the broad UV–Vis features and the comparison with literature precedents.

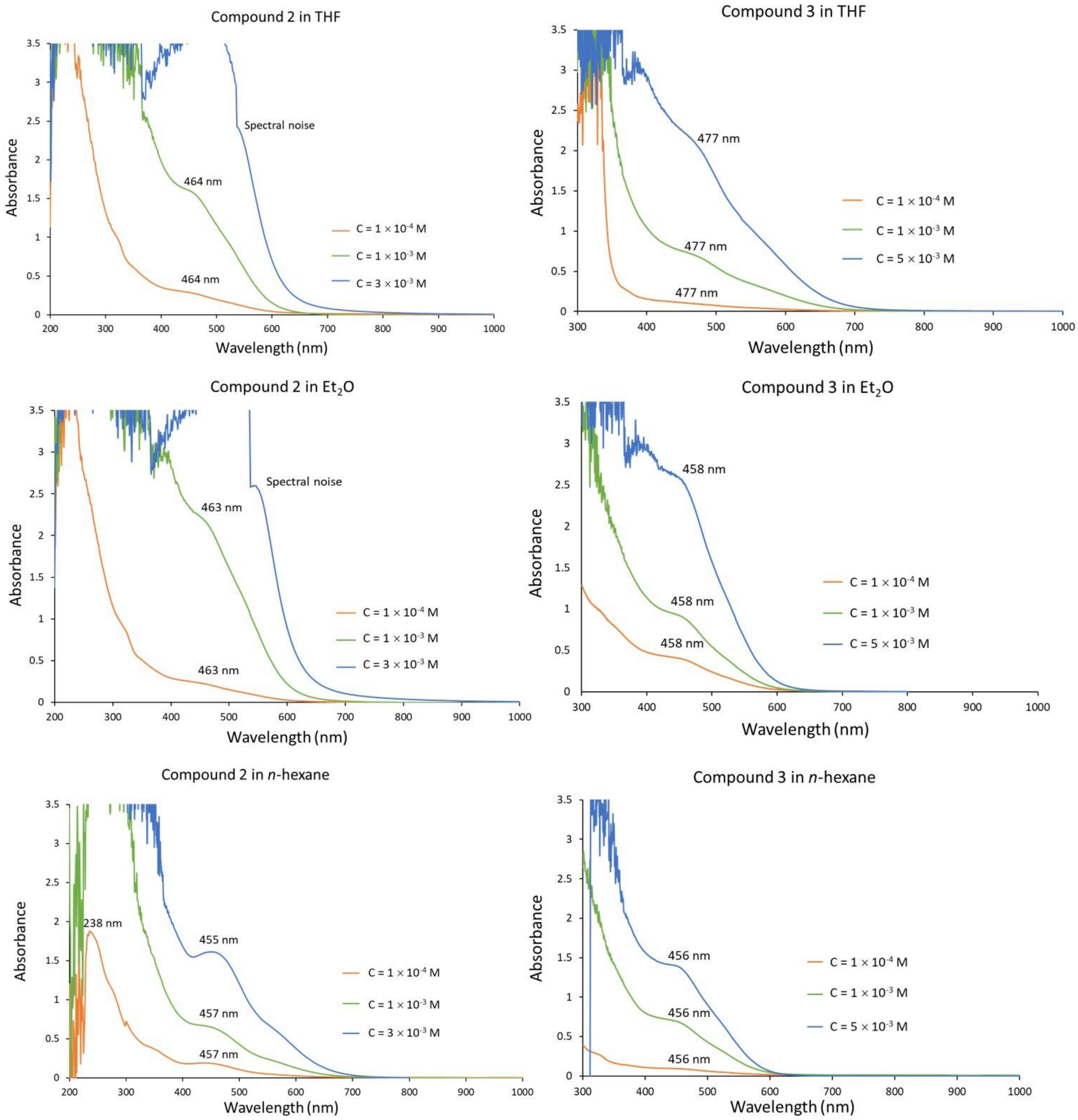

To address this point, we have carried out carefully controlled UV–Vis measurements of compounds **2** and **3** under different concentrations and in different solvents (THF,

Et_2O and *n*-hexane), using thoroughly degassed and dried solvents to exclude decomposition.

The results show that at relatively high concentration (3×10^{-3} M), no distinct absorption band is observed in the long-wavelength region for compound **2** in THF or Et_2O; only minor spectral noise are present. Even in *n*-hexane at comparable concentration, only an absorption around 455 nm is observed. At lower concentrations, well-defined absorption maxima are consistently observed at 464 nm (THF), 463 nm (Et_2O), and 457 nm (*n*-hexane) for compound **2**. An analogous concentration-dependent behavior is also observed for compound **3**

In addition, TD-DFT calculations consistently predict that the maximum absorption bands of compound **3** lie at approximately 450 nm with weak intensity, in good agreement with the experimental data (Figures S34 and S35).Taken together, these results demonstrate that the absence of a strong red-shifted absorption in our system is intrinsic to the electronic structure of these isolated radical anions, rather than a consequence of instability. The Supporting Information have been revised accordingly to include the new UV–Vis data.

Comment 14: The UV-Vis spectra needs to be repeated under very rigorous conditions, and if sharper peaks are not found, then an explanation for this should be included.

Response: please see the above comment.

Comment 15: A comparison between the calculated and experimental UV-Vis data needs to be included.

Response: We thank the reviewer for this helpful suggestion. the calculated and experimental UV-Vis data have been included in the manuscript and the supporting information accordingly

Comment 16: The UV-Vis spectra and the calculated UV-Vis spectra do not match – please increase the wavelength range of the spectra to the THF cutoff (ie 220 nm) to see if greater match is possible. If not, then please provide and explanation for the difference.

Response: We thank the reviewer for this helpful suggestion. As shown in the revised UV-Vis spectra, compound **2** in THF exhibits significant spectral noise below 300 nm, even at a relatively low concentration (1.0×10^{-4} M), which prevents reliable

assignment of absorption features in this region. In contrast, when measured in *n*-hexane at the same concentration, a distinct absorption band is observed at 238 nm. Compound **3** shows a similar behavior: at low concentrations and across different solvents, the region below 300 nm is dominated by noise, with no well-defined absorption bands that can be confidently assigned. Consequently, extending the measurement down to the THF cutoff (220 nm) does not provide additional meaningful spectral information. Nevertheless, the absorption band observed for compound **2** in *n*-hexane at 238 nm is in agreement with the calculated value (226 nm). Overall, the theoretical results are consistent with the experimental data.

Comment 17: Compound 6 experimental: State how much water was added.

Response: We thank the reviewer for this helpful suggestion. We added 10 equivalents of water to compound **2** to generate compound **6**. This information has now been clarified in the main text (Figure 5) as well as in the Supporting Information (Section 6.4).

Comment 18: Compound 4 : The authors need to refer the "crude product" as "crude reaction mixture" as the former implies an isolated compound.

Response: We thank the reviewer for this helpful comment. We have revised the wording from "crude product" to "crude reaction mixture" in the supporting information to ensure accuracy and clarity.

Comment 19 : Compound 5: Change wording from "was decomposed in acetonitrile" to "decomposed in acetonitrile"

Response: Corrected

Comment 20 : Compound **8**-D2 : Is it possible to run a 2H NMR spectrum of this compound to confirm the deuterium signal?

Response: We thank the reviewer for this helpful suggestion. To confirm the presence of deuterium, we synthesized compound **2** and grew suitable crystals for reaction with D_2O. The resulting sample was first analyzed by ^{1}H NMR and subsequently by ^{2}H NMR using the same NMR tube. As shown in the spectra, the ^{2}H NMR signal at 4.63 ppm is assigned to the Ge–D deuterium resonance.

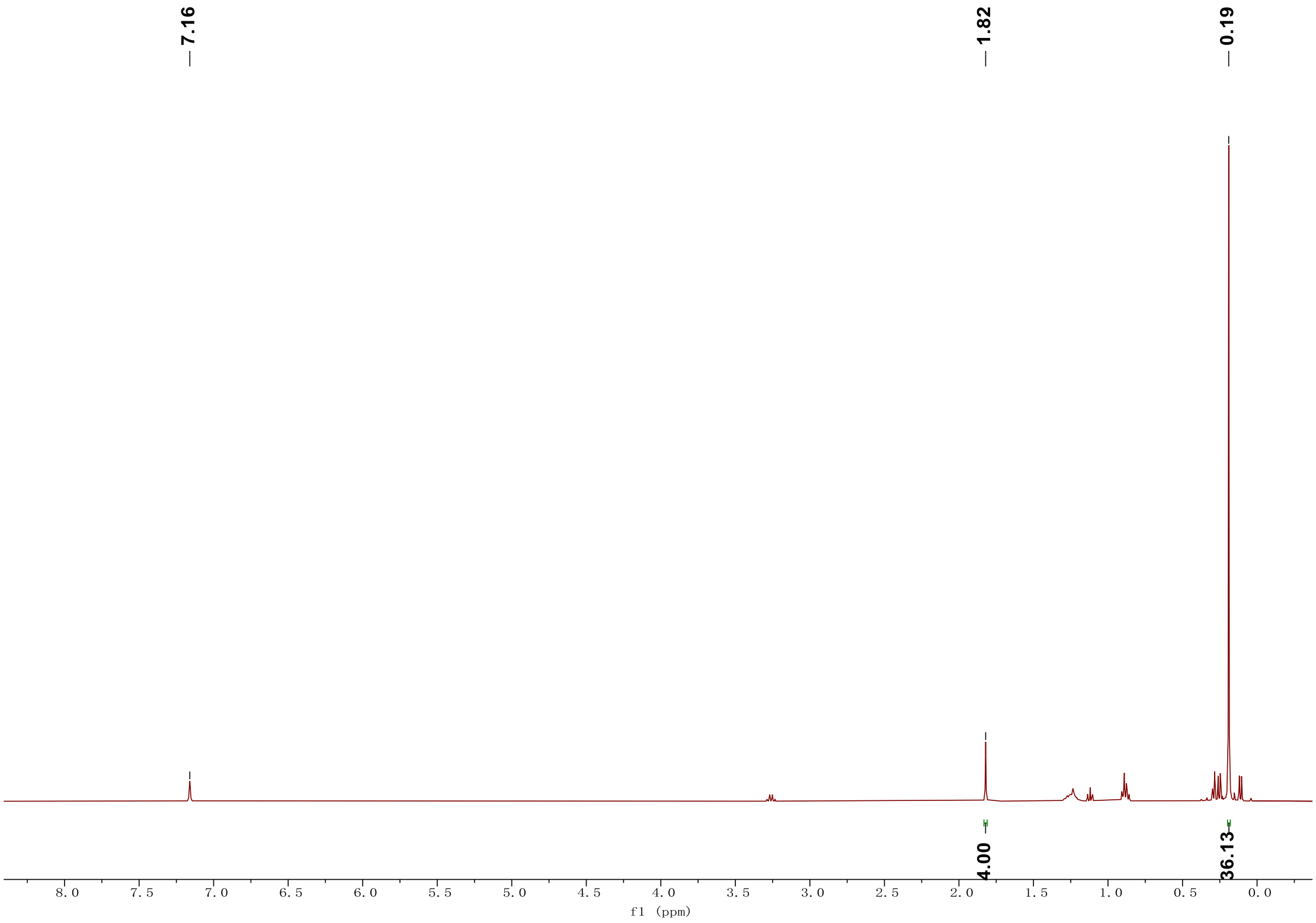

^{1}H NMR (400 MHz, benzene-d_6) spectrum of compound **8**.

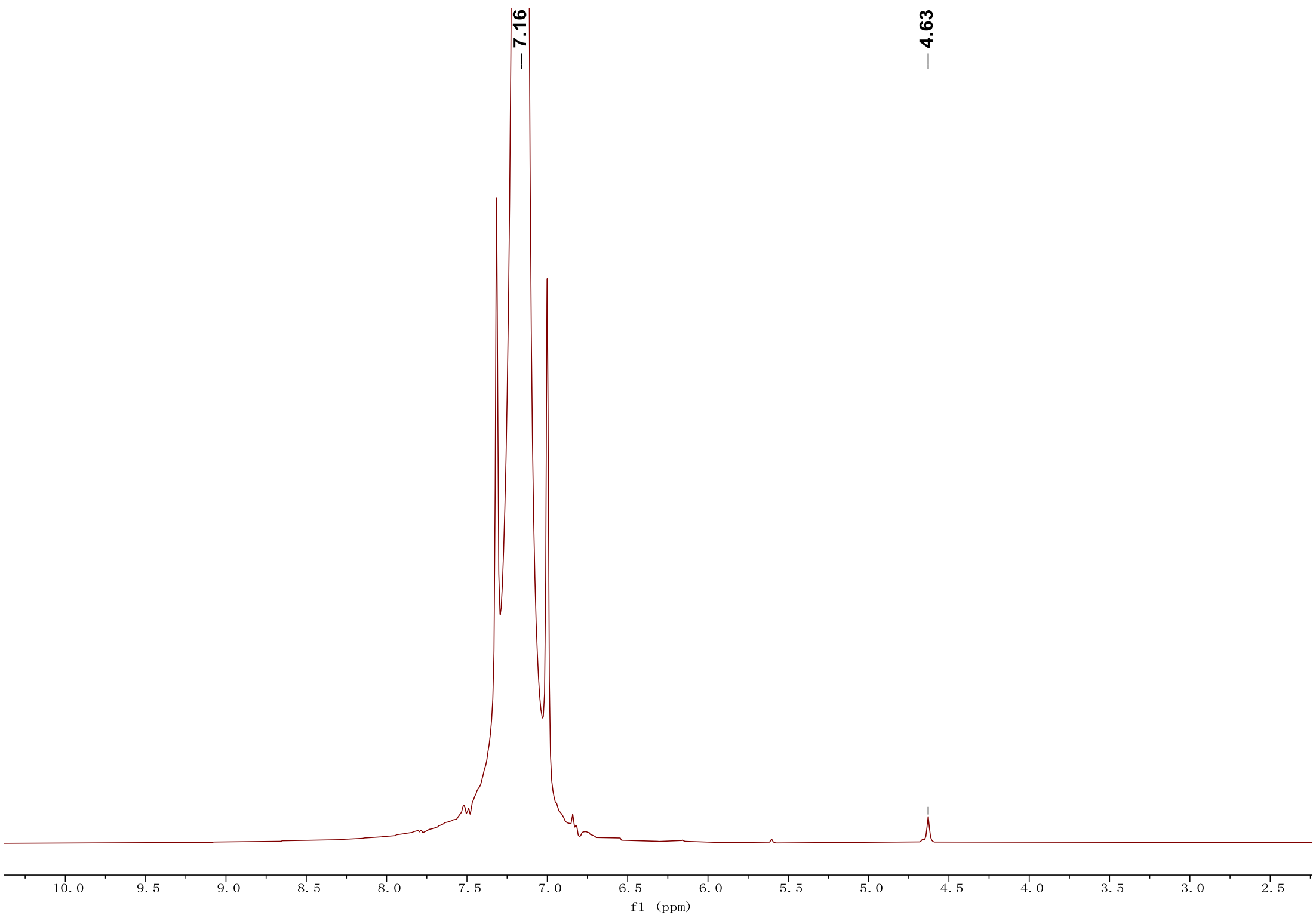

^{2}H NMR (77 MHz, benzene-d_6) spectrum of compound **8**.

Comment 21 : References: Reference 65 is for the correction and not the actual manuscript.

Response: We thank the reviewer for noting this issue. Reference 65 was mistakenly cited as the correction notice rather than the original article. We have now replaced it with the correct reference to the original manuscript. "67. M. P. Egorov, O. M. Nefedov, T.-S. Lin, P. P. Gaspar, "Germylene and Stannylene Anion Radicals: Generation and Electronic Structure" *Organometallics* **1995**, *14*, 1539-1541."

Reviewer 2:

General comments: Yan et al. report on a new germanium radical anion that is stable at room temperature and could hence be isolated and fully characterized including single-crystal X-ray diffraction. The findings add to current interest in low-valent main-group chemistry and especially radical compounds hold much potential. The work has been conducted at high level and the results presented in here are convincing. While radical anions of germanium have been reported before, they are typically unstable at ambient conditions. Using a cyclic ligand environment, the authors were able to stabilize the formal Ge(I) centre to a certain extent thus the compound is stable at room temperature. The radical anion could be isolated and characterized in two forms, i.e., a potassium bridged dimer and a monomer through complexation of 18-crown-6. Both, experiment and theory provide evidence that the spin density is mainly located at germanium. Additional reactivity studies have been conducted and underpin the properties of the radical anion. This is a very nice and timely piece of work. I am sure it will receive broad attention and is hence well suited for the readership or Nature Communications. I fully support acceptance and have only a few minor comments:

Response: We greatly appreciate the reviewer for the positive assessment of our work. We have carefully revised the manuscript in accordance with your professional and constructive comments, and our detailed responses to each of your suggestions are provided below.

Comment 1 : The statement "solely by steric protection, without electronic perturbations" appears confusing. Whenever a ligand is used, both steric and electronic effects play a role. Why that should not be the case here?

Response: We thank the reviewer for this important comment. We agree that both steric and electronic effects are generally involved when ligands are employed. In our case, the design primarily emphasizes steric protection while minimizing electronic

perturbation. Specifically, dialkyl-substituted ligands are typically sterically dominated and among the least electronically perturbed. In “The First Isolable Dialkylsilylene” (*J. Am. Chem. Soc.* **1999**, *121*, 9722–9723), the alkyl-substituted ligand is described as a helmet-like bidentate ligand, and the silylene is well protected sterically from dimerization while being least perturbed electronically. To avoid ambiguity, we have revised the wording accordingly. The description has been clarified in the manuscript as: Herein, we report the successful isolation and full characterization of an alkyl-substituted germanium(I) radical anion predominantly stabilized by steric protection with minimal electronic perturbation.

In addition, we have also deleted “solely” in the summary section and revised the sentence as follows: In summary, we have successfully isolated and fully characterized the alkyl-substituted Ge(I) radical anion, which is an unprecedented example of an electronically unbiased main group open-shell species stabilized by σ-donating alkyl substituents.

Comment 2 : The reactivity section falls very short. Give the nice results I suggest that the authors add two or three paragraphs to discuss their findings in more detail. Especially compound 4 and 5 deserve this.

Response: We thank the reviewer for this valuable suggestion. In response, we have expanded the discussion of the reactivity section, with additional description provided for compounds **4** and **5**. The revised text was incorporated in the main text:

Comment 3 : Figure 5: using a, b, c, and d is confusing please move the reagents from the caption to the figure and add yields.

Response: We thank the reviewer for this helpful suggestion. We have revised Figure 5 accordingly by moving the reagents from the caption into the figure and adding the corresponding yields, which improves the clarity and readability of the scheme.

Comment 4 : IR-spectra of compound 4-6 could be very insightful.

Response: We thank the reviewer for this valuable suggestion. We have performed IR measurements for compounds **4–6**, and the corresponding spectra have been included in the Supporting Information.

Comment 5 : As the compounds are colourful, I suggest measuring the UV-vis data and maybe comparing them with the computations.

Response: We thank the reviewer for this valuable suggestion. We have carefully measured the UV-vis spectra of **2** and **3**, all the spectra were add to SI (Figs S2-S8). The radical anion **2** exhibits a weak absorption peak at 464 nm in THF, while **3**·K(18-C-6) exhibits a weak absorption peak at 477 nm. The calculated results for **3** (454 nm) is in agreement with the experimental data (Figure S34). The computatational results have been included in the supporting information.

Comment 6 : Compound 5 could not be fully characterized due to decomposition in acetonitrile. I do not why the instrument runs only with acetonitrile as these instruments can be used with almost every solvent. Hence, an additional technique should be applied like elemental analysis.

Response: We sincerely thank the reviewer for this valuable comment. Given the unsatisfactory results, we have therefore re-measured all compounds by using THF and successfully obtained the corresponding data. All updated HRMS results have been included and highlighted in the Supporting Information.

Comment 7 : The deviation of the HRMS results is in some cases too high. This should be checked and revised.

Response: We thank the reviewer for this helpful comment. As noted above, all compounds have been re-measured by HRMS using THF as the solvent to improve data quality. The results have been carefully verified, and the deviations have been significantly reduced. All revised HRMS data have been updated in the Supporting Information.

Reviewer 3 :

General comments: In this work, Yan and co-workers report the synthesis, characterization, and reactivity of a cyclic dialkyl Ge(I) radical anion generated via reduction of the corresponding germylene or dichlorogermane precursors. Isolable Ge(I) radical anions remain exceedingly rare, with prior examples essentially limited to the system reported by Hicks et al. (compound II in the manuscript). In this regard, the present study expands the small family of structurally authenticated Ge(I) radical anions. The reported species is new within the germanium radical anion context. However, it should be noted that the underlying ligand framework has previously been employed to stabilize related main-group radical species, including a persistent dialkylsilylene radical anion reported as early as 2003, as well as isolable phosphinyl radicals and related open-shell systems. Consequently, while the work represents a

solid incremental advance, its conceptual novelty appears limited. In my opinion, the study does not fully meet the level of conceptual innovation and general significance typically expected for publication in Nature Communications. Overall, the manuscript presents careful experimental work and valuable characterization data. Addressing the points above would improve clarity and strengthen the mechanistic and electronic interpretations; however, in its current form, the advance appears incremental relative to prior literature.

Response: We sincerely thank the reviewer for the thoughtful evaluation and for recognizing the careful experimental work and valuable characterization presented in this study. We appreciate the reviewer's comments regarding the perceived incremental nature of the advance and would like to clarify several important points concerning both the rarity and the significance of the present system.

First, isolable Ge(I) radical anions remain exceedingly rare. To the best of our knowledge, only two such species have been reported to date, and structurally authenticated examples are essentially limited to a single precedent (*Angew. Chem. Int. Ed.* **2022**, 61, e202201248). Therefore, the successful isolation and full structural characterization of a new Ge(I) radical anion already represents an important expansion of this highly limited class of compounds.

Second, although related ligand frameworks have been employed in stabilizing other main-group radicals, key distinctions should be emphasized. For example, the previously reported dialkylsilylene radical anion (*J. Am. Chem. Soc.* **2003**, *125*, 3212-3213) was generated in situ and characterized primarily by EPR spectroscopy without isolation, purification and structural confirmation. Moreover, no subsequent reactivity studies were reported. In contrast, our work provides an isolable and crystallographically characterized Ge(I) radical anion, and systematic investigations of its reactivity, thereby offering a more complete understanding of such open-shell low-valent main group radicals.

Third, we believe that the present work contributes to a broader and actively developing context of germanium redox chemistry and main-group catalysis. Recent studies have demonstrated that low-valent germanium compounds can participate in redox cycles (Ge(II)/Ge(III)/Ge(IV) or Ge(II)/Ge(IV)), enabling bond activation and catalytic transformations (*J. Am. Chem. Soc.* **2025**, 147, 36752−36762). These processes frequently involve single-electron transfer steps and radical intermediates,

highlighting the importance of well-defined germanium-centered radical species for mechanistic understanding. Similarly, low-valent germanium compunds have been shown to catalytic activate inert C(sp^3)–F bonds (*J. Am. Chem. Soc.* **2026**, *148*, 11176−11186), further underscoring the relevance of understanding their electronic structures and reactive intermediates. In this context, isolable Ge-centered radical anions might provide valuable insights for the germanium-mediated redox catalysis.

Taken together, we believe that this work represents more than an incremental advance, as it expands the extremely limited family of structurally characterized Ge(I) radical anions, demonstrates that dialkyl-substituted ligands can effectively stabilize such species. Importantly, it might provide direct experimental insight into their reactivity as well as their potential roles as key intermediates in main group redox catalysis.

Given the growing interest in main group catalysis and the recognized importance of the isolation and characteration of highly reactive radical intermediates, we believe that our findings offer timely and relevant insights that will stimulate further developments in the field. Therefore, we believe that the present work aligns well with the aims and scope of *Nature Communications*.

Comment 1 : The authors compare radical anion II with alkyl-substituted Ge(I) radical anions and conclude that the latter are electronically non-perturbing. However, the manuscript does not clearly explain how the diamido ligand perturbs the electronic structure of compound II. A more explicit analysis—supported by orbital descriptions, spin-density distributions, or quantitative bonding metrics—would be necessary to substantiate this claim.

Response: We thank the reviewer for this important comment. We agree that our original discussion did not sufficiently clarify how the diamido ligand perturbs the electronic structure relative to dialkyl-substituted systems.

Previous studies have shown that nitrogen-containing ligands such as *β*-diketiminate and other *N*-heterocyclic frameworks, are often not completely electronically innocent and can participate in the electronic structure of low-valent germanium radicals through spin delocalization. For example, in a *β*-diketiminate germanium(I) radical, although the SOMO is described as an almost pure Ge-centered p-orbital, EPR/ENDOR and DFT analyses indicate partial spin delocalization onto the ligand backbone, highlighting that the *β*-diketiminate ligand is not fully redox innocent (*J. Am. Chem. Soc.* **2011**, *133*,

10074–10077). Moreover, a later systematic study demonstrated that changing the substituents on the *β*-diketiminate framework can switch the electronic structure from a Ge-centered p-type radical to a ligand-centered π-type radical, with the SOMO located on the NC_3N backbone and spin density mainly distributed over N/C atoms rather than Ge (*Inorg. Chem.* **2019**, *58*, 5688−5694). In addition, an acyclic Ge(I) radical, using a combination of an amido ligand and a cyclic(alkyl)-(amino)carbene (cAAC) show significant resonance contributions with the unpaired electron localized on the cAAC ligand . These results collectively indicate that *N*-based ligands can introduce notable electronic perturbation.

Improtantly, although the SOMO of compound II is described as an almost exclusive unhybridized p-orbital centered on Ge, the diamido ligand is not electronically irrelevant. The increased Ge⋯O separation was explicitly attributed to an antibonding interaction between the oxygen lone pair and the unpaired electron at the Ge(I) center. In addition, small but non-zero $^{14}N/^{1}H$ hyperfine couplings show that a minor amount of spin density is still detected on the ligand. Thus, compound II is predominantly Ge-centered, but its N/O-containing diamido framework still modulates the electronic structure through donor interactions and limited spin delocalization

In contrast, dialkyl-substituted ligands are generally considered as typically sterically dominated and lack low-lying π-systems, thereby minimizing electronic interaction with the central atom. Such ligands are sterically dominated and in the seminal work “The First Isolable Dialkylsilylene” (*J. Am. Chem. Soc.* **1999**, *121*, 9722–9723), the dialkyl-substituted ligand is described as a sterically protecting “helmet-like” framework that stabilizes the reactive center while being among the least electronically perturbing.

In our case, DFT calculations show that the SOMO is predominantly composed of the Ge 4*p* orbital, and spin density analysis confirms that the unpaired electron is essentially localized on the germanium center. Therefore, compared to diamido-supported systems, our ligand environment leads to a significantly reduced degree of electronic perturbation. We have revised the manuscript accordingly to clarify this distinction.

Comment 2 : In compound II, describing the ligand as an N,O-donor may be misleading. Structurally and electronically, the ligand functions more appropriately as a diamido ligand. The terminology should therefore be revised for accuracy and consistency with established conventions.

Response: We thank the reviewer for this insightful comment. We agree that describing the ligand as an N,O-donor may be misleading. Accordingly, we have revised the terminology in the main text and now refer to it as a diamido ligand for greater structural and electronic accuracy.

Comment 3 : The EPR measurements reveal notable spin–nuclear coupling with silicon nuclei. It would therefore be important to clarify whether the computational analysis identifies any significant orbital interaction or spin delocalization between the silyl substituents and the germanium center. Discussion of relevant molecular orbitals, spin populations, or hyperfine coupling calculations would help rationalize the experimental observations.

Response: We thank the reviewer for this insightful comment. We have carefully discussed the frontier molecular orbital and spin population of **3** in our revised manuscript (pages 7 and 8). Moreover, the calculated electron-spin g-factor and hyperfine coupling constants for **3**·K(18-C-6) are g = 2.0129, $a(^{29}Si)$ = 13.24 G, $a(^{29}Si)$ = 11.12 G, $a(^{73}Ge)$ = 4.78 G, which are in agreement with the experimental results (g = 2.0127, $a(^{29}Si)$ = 12.87 G, $a(^{29}Si)$ = 10.39 G, $a(^{73}Ge)$ = 5.08 G.). The corresponding results have been added to the SI.

With best wishes

Chenting Yan

Hangzhou, June 5, 2026

Dear Reviewer #2,

We sincerely appreciate the detailed feedback and the positive and helpful comments provided by the reviewer #2 on our manuscript. The guidance offered has been instrumental in enhancing the quality and clarity of our work. We have carefully considered the reviewer's comments and have made the necessary revisions to improve the article. The following changes have been made:

Reviewer 2:

General comments: The authors provided a revised version of their initial submission and a very detailed response, which is appreciated. The points raised by the reviewers have been addressed properly and the majority of the reviewers considers the work suitable for publication in Nature Communications. Indeed, there is precedence but there is also interesting reactivity and, in my eyes, enough novelty to warrant publication in Nature Communication. Hence, I recommend publication and have only very few formal comments to be addressed.

Response: We thank the reviewer for the positive evaluation and strong support of this work. We have carefully revised the manuscript in accordance with your professional and constructive comments, and our detailed responses to each of your suggestions are provided below.

Comment 1: Abstract: Please remove "exceptional" in the sentence "This radical anion exhibits exceptional thermal stability in both the solid state and in solution" as it is too much over-selling.

Response: Thank you for this valuable suggestion. Following your comment, we have removed the word "exceptional" from the relevant sentence in the abstract accordingly in the revised manuscript.

Comment 2: Figure 2a and S1: Please add an arrow that indicates the direction of scanning the voltage.

Response: We thank the reviewer for the helpful suggestion. We have supplemented arrows to clearly mark the voltage scanning direction in Figure 2a and Figure S1 in the revised version of the manuscript and supporting information.

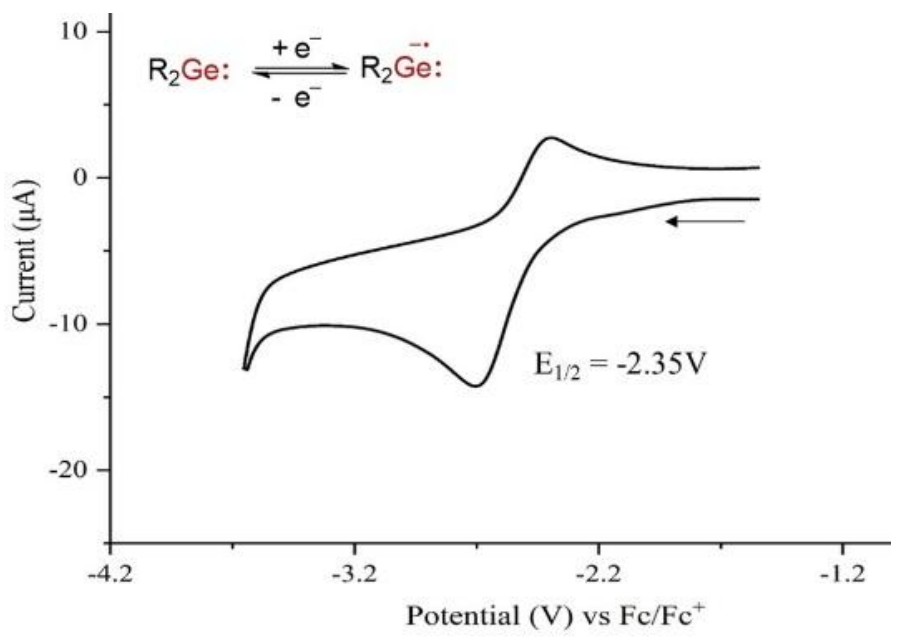

Figure 2. a) Cyclic voltammogram of **1** at 0.1 V s^{-1} scan rate in THF / 0.1 M [n-Bu_4N][PF_6].

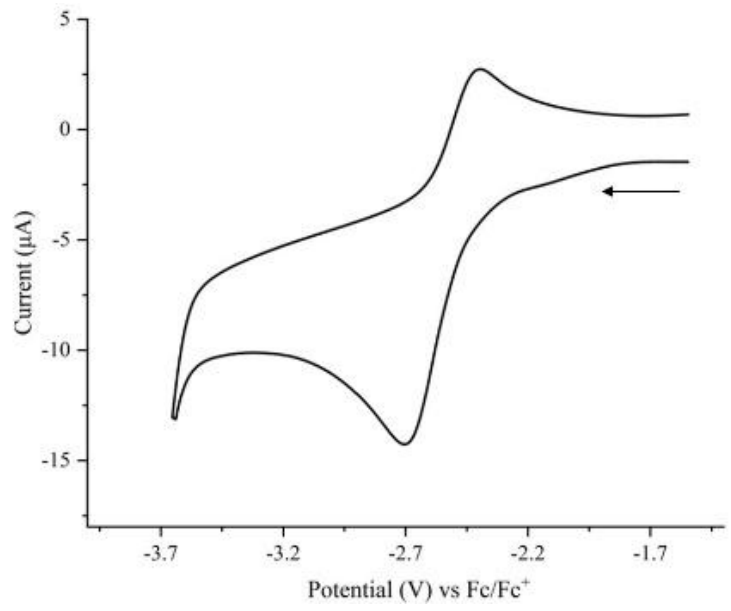

Fig. S1. Cyclic voltammogram of germylene **1** in THF / 0.1 M [n-Bu_4N][PF_6].

Comment 3: Figure 2b: I would include the synthetic route starting from cmpd. 7 as this is the most suitable and high-yielding route towards 2.

Response: We appreciate the reviewer's constructive advice. The synthetic route starting from compound **7** has been incorporated into Figure 2b in the revised manuscript as suggested.

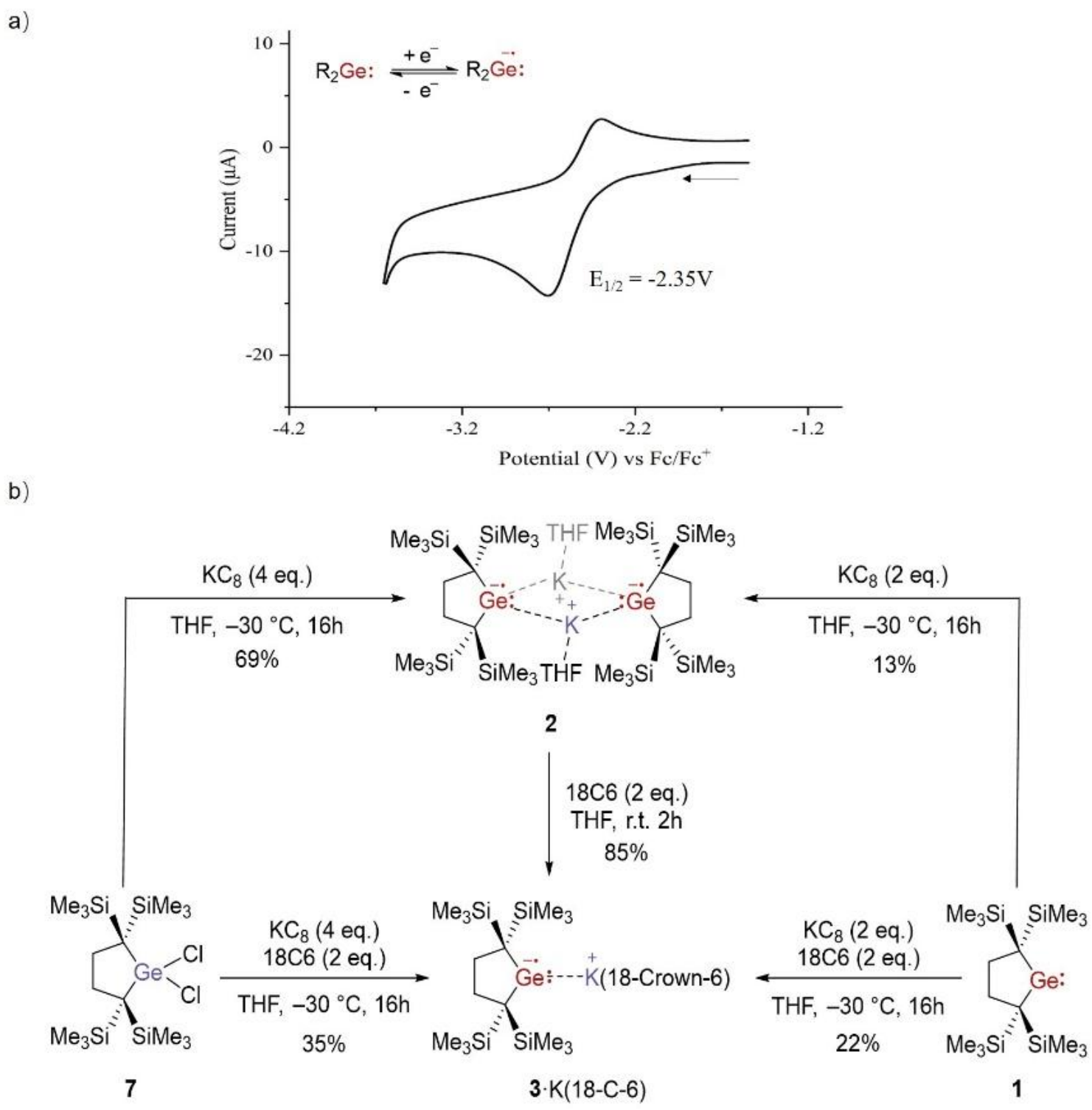

Figure 2. a) Cyclic voltammogram of **1** at 0.1 V s^{-1} scan rate in THF / 0.1 M [n-Bu_4N][PF_6]. b) Synthesis of germanium radical anions **2** and **3**·K(18-C-6).

Comment 4: Figure 3 & 5: Please add “in the solid state” as these are molecular solid-state structures

Response: We are grateful for the reviewer’s careful reminder. The phrase “in the solid state” has been supplemented in the Figure 3 and Figure 5 in the revised manuscript as recommended.

Comment 5: Figure 5: Please add information on the probability and which atoms you omitted.

Response: Thanks for the comment. Details on probability and omitted atoms have been added to Figure 5.

With best wishes

Chenting Yan
